# Room-Temperature-Stable Magnesium Electride via Ni(II) Reduction

**DOI:** 10.1021/jacs.2c01807

**Published:** 2022-07-13

**Authors:** Craig S. Day, Cuong Dat Do, Carlota Odena, Jordi Benet-Buchholz, Liang Xu, Cina Foroutan-Nejad, Kathrin H. Hopmann, Ruben Martin

**Affiliations:** † Institute of Chemical Research of Catalonia (ICIQ), The Barcelona Institute of Science and Technology, Av. Països Catalans 16, 43007 Tarragona, Spain; ‡ Departament de Química Analítica i Química Orgànica, Universitat Rovira i Virgili, c/Marcel·lí Domingo, 1, 43007 Tarragona, Spain; ^§^Hylleraas Center for Quantum Molecular Sciences and ^∥^Department of Chemistry, UiT The Arctic University of Norway, N-9037 Tromsø, Norway; ⊥ Institute of Organic Chemistry, Polish Academy of Sciences, Kasprzaka 44/52, 01-224 Warsaw, Poland; # ICREA, Passeig Lluís Companys, 23, 08010 Barcelona, Spain

## Abstract

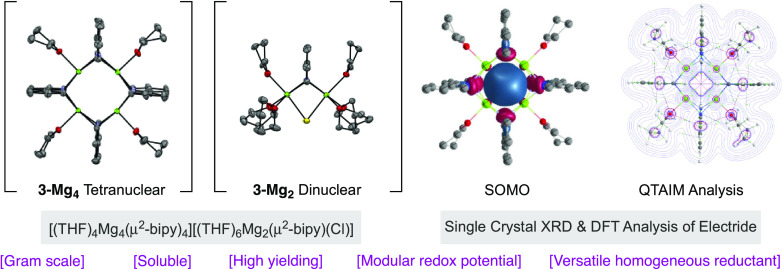

Herein, we report the synthesis of highly reduced bipyridyl magnesium complexes and the first example of a stable organic magnesium electride supported by quantum mechanical computations and X-ray diffraction. These complexes serve as unconventional homogeneous reductants due to their high solubility, modular redox potentials, and formation of insoluble, non-coordinating byproducts. The applicability of these reductants is showcased by accessing low-valent (bipy)_2_Ni(0) species that are challenging to access otherwise.

## Introduction

**Scheme 1:**
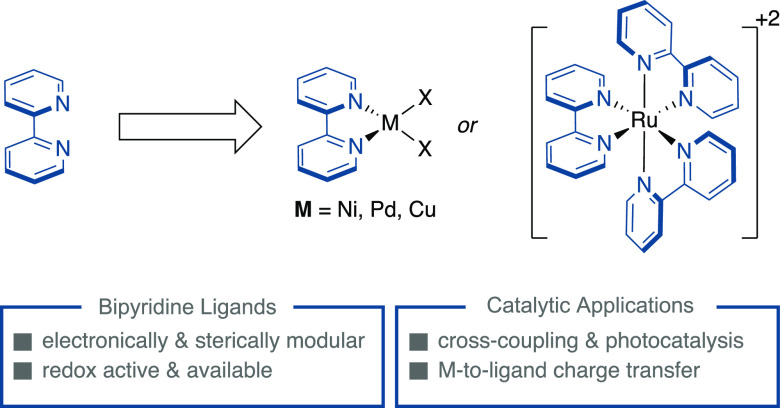
Inherent Interest of Bipyridine Scaffolds

Bipyridine ligands have historic and prosperous relationships with main group, transition metal, and materials chemistry.^1^ Cited as “the most widely used ligand”,^2^ the popularity of bipyridines is ascribed to their robust synthesis, tunable steric and electronic properties, modular σ-donation of the nitrogen atoms, and π–accepting molecular orbitals. In addition, the redox non-innocent character of the bipyridyl core^3^ and the involvement of metal-to-ligand charge transfer events have enabled new catalytic redox transformations of utmost synthetic relevance for our chemical portfolio (Scheme 1).^4^

**Scheme 2:**
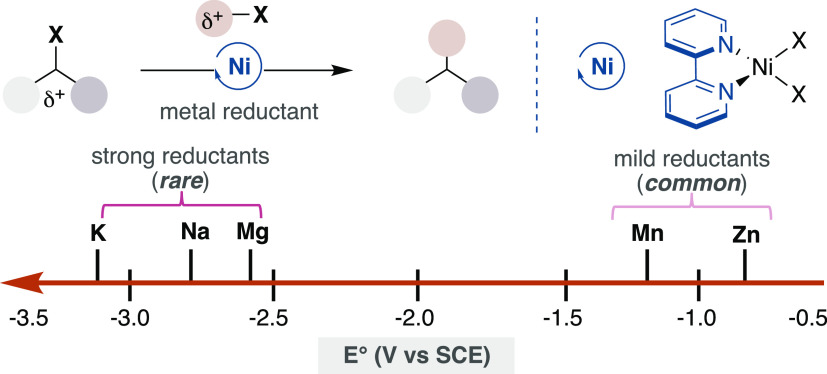
Reductant Compatibility in Nickel Catalysis

While significant efforts have been made in characterizing ligand parameters within the context of Ni-catalyzed reactions,^5−8^ the elucidation of the fundamental reactivity, speciation, and redox non-innocence of bipyridine ligands still remains the subject of considerable debate compared to their redox-innocent PR_3_ and NHC analogues.^9−11^ This is particularly the case for Ni-catalyzed reductive coupling reactions involving redox manifolds where bipyridine ligands play a critical, yet not fully understood role in both reactivity and selectivity.^12^ A close inspection into the literature data reveals an intriguing threshold in the reductants that are compatible in Ni-catalyzed reductive cross-coupling reactions (Scheme 2).^12^ While milder Mn or Zn reductants have become routine in these processes, the utilization of stronger reductants such as Mg has only found echo in Ni-catalyzed reactions supported by redox-innocent NHC or PR_3_ ligands.^13^ Prompted by the mechanistic ambiguity surrounded by the use of heterogeneous metal reductants and the perception that single electron transfer might be turnover limiting in these processes,^14^ we anticipated that further investigations might uncover opportunities to explore inaccessible chemical spaces while leading to new knowledge in the Ni-catalyzed reductive coupling arena. Herein, we describe our efforts toward this goal.

## Results and Discussion

We began our investigations by monitoring the stoichiometric reduction of (**L1**)NiCl_2_**1** (**L1** = 2,2′-bipyridine) with Mg (Mg^2+/0^ = −2.61 V vs SCE) in THF-*d*_8_ with additional ligands to stabilize unsaturated nickel species that might be generated upon reduction. Interestingly, upfield signals (δ = 6.7–4.1 ppm) were observed by ^1^H NMR spectroscopy after 4 h, which we tentatively attributed to reduced, anionic [(bipy)_2_Ni]^−^ entities. Such speciation is consistent with cyclic voltammetry studies performed by ourselves^15^ and Bartak,^16^ where reduction of (bipy)_2_Ni (**2**) occurs at ca −2.0 V to afford reduced (bipy)Ni species.^17^ Workup of the reaction afforded a temperature-stable purple powder, which upon crystallization from THF/pentane at −36 °C led to the unambiguous characterization of an intriguing tetranuclear/dinuclear **3-Mg**_**4**_**/3-Mg**_**2**_ couple [(THF)_4_Mg_4_(μ^2^-bipy)_4_][(THF)_6_Mg_2_(μ^2^-bipy)(Cl)] **3**, which may be considered as two neutral entities or an ion pair. The identity of **3** was confirmed by X-ray crystallography, and reproducibly solved 16 times.^15^ Interestingly, the ^1^H NMR signals of **3** overlayed to those observed *in situ* upon stoichiometric reduction of (bipy)NiCl_2_**1** with Mg, indicating that our tentative assignment of an anionic [(bipy)_2_Ni]^−^ complex was incorrect, with nickel likely forming unligated Ni(0) that deposits as nickel black, which is filtered off upon workup.^18^ This observation gains credence considering the poor adoption of strong reductants such as magnesium in nickel-catalyzed reactions using redox-active ligands.^12^ While [(bipy^–1^)(bipy)Ni] may initially form, we anticipate that the electron-rich bipy^1–^ would be a very poor ligand for electron-rich Ni(0) and readily dissociate, resulting in decomposition that ultimately results in the formation of Ni black.

**Figure 1:**
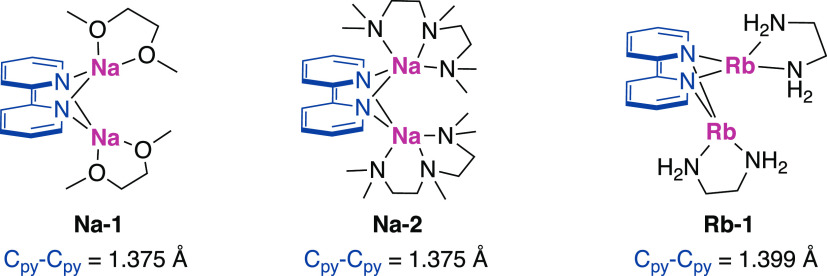
Alkali and Alkali Earth Metal–Bipy Complexes.^19,20^

Comparison of the interpyridyl C_py_–C_py_ bonds of **3** with bipyridine radical anions (bipy^1–^, 1.429 Å)^20^ or free ligand (bipy, 1.494 Å)^21^ reveals that **3** contains particularly contracted C_py_–C_py_ linkages ranging from 1.369(5) to 1.382(5) Å (avg 1.376 Å). These interpyridyl C_py_–C_py_ bond lengths of **3** are comparable to bipy^2–^ complexes of alkali metals^19,20^ (Figure 1) with bond lengths similar to [bipy^2–^][Na^+^(dme)]_2_**Na-1**, [bipy^2–^][Na^+^(pmdta)]_2_**Na-2** and its [bipy^2–^][Rb^+^(en)]_2_**Rb-1** analogue. This drastic contraction reports directly on the localization of the electron density, suggesting electron occupation of the π*-antibonding orbital and increased bonding character between the in-phase C_py_–C_py_ bonds, which results in an antiaromatic bipyridine ring system on the basis of the ring current analysis (ring current strength: −2.6 n.A.T^–1^; see computational methods in the Supporting Information).^19,20,22,23^ The rare, symmetric binding mode of bipyridine ligands in **3** indicates that two bonding interactions are available from the σ-N p orbital lone pair and highest occupied molecular orbital (HOMO) π* orbital to magnesium, further advocating the notion that a bipy^2–^ entity is generated upon reduction of the bipyridyl core. The symmetric macrostructure and unique bond angles found in **3**, with Mg–bipy–Mg bond angles of 103° for the edges in **3-Mg**_**4**_ and 109° for the binuclear moiety **3-Mg**_**2**_ are among the few examples bearing symmetrically bridging μ^2^-bipy ligands such as (**Na-1** and **Na-2**) or trinuclear species (Yb(μ^2^-bipy)(THF)_2_)_3_^22^ with no similar tetranuclear structures reported before. While no Mg–bipy^2–^ complexes have been reported to compare the Mg–N bond distances of **3** to which range from 2.189(3) to 2.244(3) Å (avg 2.216 Å), a comparison between bipy^2–^ alkali metals **Na-1**, **Na-2**, and **Rb-1** reveals that **3** contains the shortest M–N bond, followed by **Na-1** (2.37 and 2.40 Å). We suspect the short N–Mg bond is due to the absence of chelated electron-donating ligands such as dme, en, or pmdta, which results in the Mg center of **3** being more Lewis-acidic, thus shortening the Mg–bipy^2–^ bond.

**Figure 2:**
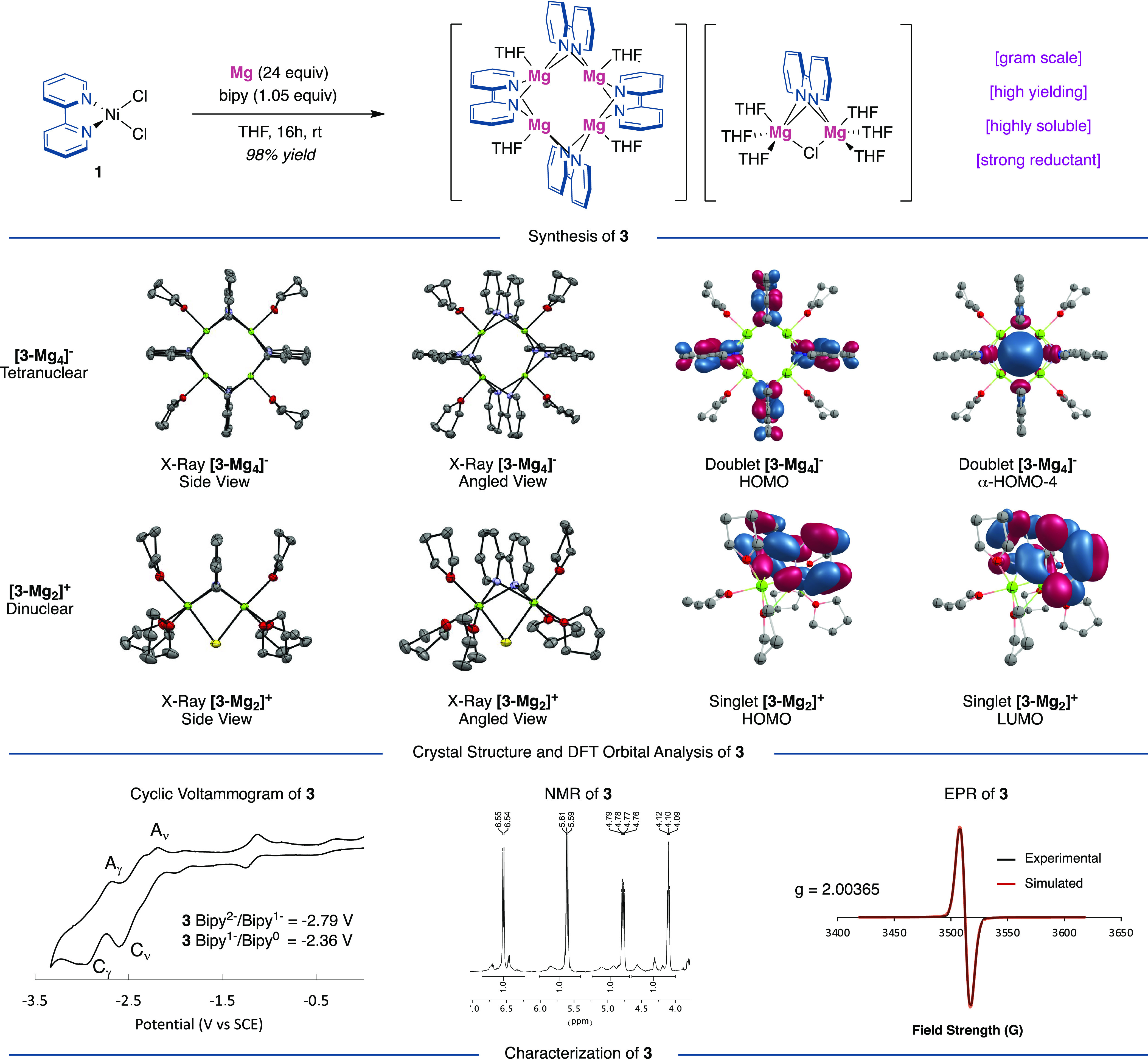
Synthesis, crystal structure, characterization, and DFT orbital analysis of **3-Mg_4_** and **3-Mg**_**2**_. X-ray structures are shown with thermal ellipsoids drawn at the 50% probability level (see the Supporting Information for details and labeled structures). Selected distances (Å): C_py_–C_py_; C5B–C6B 1.374(5), C5A–C6A 1.382(5) Example Mg–Mg distance; Mg1B–Mg2B 2.8246(16). HOMO, lowest unoccupied molecular orbital (LUMO), and singly occupied molecular orbital (SOMO) (HOMO-4) orbitals for **3-Mg**_**4**_ and **3-Mg**_**2**_ (PBE0-D3BJ/6-31+G(d,p)[IEFPCM:THF]). Cyclic voltammogram performed in THF with 0.1 M NBu_4_PF_6_.^1^H NMR (400 MHz) in THF-*d*_8_. X-band EPR spectrum (THF, 293 K).

UV–vis absorption and IR data corroborated the designation of a bipyridine dianion in **3**.^15^ In addition, variable-temperature, DOSY & EXSY ^1^H NMR spectroscopic experiments suggested that the multiple ^1^H NMR signals observed at room temperature (Figure 2, bottom) originate from fluxional lower-order and higher-order aggregates that readily interchange, and coalesced to the major signals upon warming (ca. 55 °C). Quantitative X-band electron paramagnetic resonance (EPR) measurements in tetrahydrofuran (THF) of **3** also revealed the presence of a single electron at *g* = 2.00365 (Figure 2, bottom right), which was consistent with analysis by Evan’s method, which determined an effective magnetic moment (μ_eff_) of 1.80 μB. Taking all of these observations into consideration, we initially assumed the electronic structure of **3** as a neutral **3-Mg**_**4**_ and radical **3-Mg**_**2**_ unit with mixed valency Mg(I)–Cl and Mg(II) centers. However, density functional theory (DFT, PBE0-D3BJ/6-31+G(d,p)[IEFPCM:THF])^15^ calculations showed that the optimized geometries of the neutral **3-Mg**_**4**_**/3-Mg**_**2**_ couple deviated significantly from the experimental X-ray structure. Further computation revealed that an ion pair of singlet [**3-Mg**_**2**_]^**+**^ and radical [**3-Mg**_**4**_]^−^ is 11.7 kcal/mol more stable than the neutral structure and exhibits bond distances closely matching our experimental data.

The [**3-Mg**_**2**_]^**+**^/[**3-Mg**_**4**_]^−^ ion-pair formation likely originates from the significant instability of Mg(I) atoms,^24^ which rapidly lose an electron to form the [**3-Mg**_**4**_]^−^**/[3-Mg**_**2**_]^**+**^ couple. Quantum theory of atoms in molecules (QTAIM) analysis^25^ shows high atomic charges (1.75 and 1.68 for [**3-Mg**_**2**_]^**+**^ and [**3-Mg**_**4**_]^−^, respectively) and QTAIM localization indices λ(Mg) close to 10, suggesting that all Mg atoms in **3** are Mg(II).^26^ It is worth noting that for an electropositive atom, the magnitude of the QTAIM localization index represents the “formal charge after ionic approximation” that is allocating all shared electrons to the more electronegative atoms, which is consistent with the IUPAC definition of oxidation state. While **3** contains short Mg–Mg distances that average 2.8533(17) Å, comparable to those reported in Jones’s Mg(I)–Mg(I) dimer of 2.8457(8) Å,^27^ no chemical bond between Mg(II) atoms is expected as they possess no valence electron to form a bond. This observation was supported by QTAIM analysis on [**3-Mg**_**4**_]^−^.^15^ QTAIM analysis does not recover any (3,-1) critical point between the magnesium nuclei. Nevertheless, the absence or presence of (3,-1) critical points does not imply the absence or presence of chemical bonds.^28,29^ The delocalization index—a QTAIM-based direct measure of covalency—was computed between magnesium atoms to be merely 0.005, thus confirming the absence of Mg–Mg bonding. For comparison, the computed delocalization index between metals in a recently synthesized Th_3_ complex with **2e**–**3c** bonding is computed to be 0.245.^30,31^ Therefore, we attribute the short Mg–Mg distances to the bipyridine ligands templating the Mg atoms.^32^

**Figure 3:**
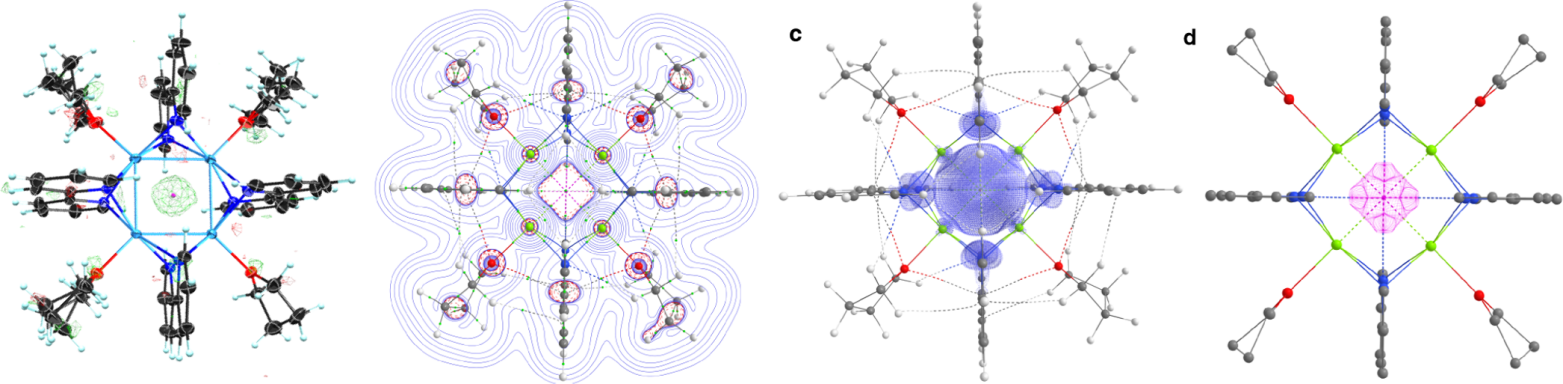
Topology of the electron density of [**3-Mg**_**4**_]^−^ containing an electride. a: Ortep-plot drawing (50%) of the X-ray structure showing residual electron density localized in the center of [**3-Mg**_**4**_]^−^, b: QTAIM contour plots of the Laplacian of the electron density, c: spin density with an isosurface of 0.05 atomic units (au, 1 au = 1 electron), and d: the atomic basin of the non-nuclear attractor that coincides with the free electron of the electride enclosed by interatomic zero-flux surfaces. The atomic basin of the non-nuclear attractor is oblong, along the C_4_ axis of the molecule; see Figures S41–S43 for side views.

With all Mg centers and bipy ligands of **3** assigned to Mg(II) and bipy^2–^, respectively, we wondered where the radical electron of [**3-Mg**_**4**_]^−^ was located. Reevaluating the EPR data of **3** (Figure 2) reveals no observed ^14^N or ^25^Mg hyperfine coupling, which suggests that the electron is not located on either the bipyridine ligands or magnesium atoms in **3**.^33^ Interestingly, the crystallographic data showed a residual electron density of 1.9 e/Å^3^ at the center of the **3-Mg**_**4**_ core (Figure 3).^34^ While the residual electron density from X-ray crystallography is not quantitatively determined, this observation was used as a qualitative guide to more closely inspect the center of the **3-Mg**_**4**_ core. While one may suggest that a hydride might be located in the center of [**3-Mg**_**4**_]^−^, our EPR experiments are inconsistent with a diamagnetic hydride formulation, which reveal a signal that does not contain any hyperfine coupling.^35^ Furthermore, quantitative EPR analysis of **3** supported the presence of a single electron, thus arguing against the presence of paramagnetic impurities. Given that a hydride might necessarily arise from THF as a hydrogen atom donor, we repeated the synthesis of **3** in THF-*d*_8_ as a source to form a deuteride instead of a hydride. A close inspection into the ^1^H NMR spectra of **3** obtained in THF or THF-*d*_8_ showed the exact same signals, thus indirectly arguing against **3** containing a hydride. Furthermore, a potential hydride should contain a proton that has no coupling with other protons as it is isolated in the center of the complex. We did not find such a proton in the ^1^H NMR spectra. Thus, we conclude that [**3-Mg**_**4**_]^−^ is unlikely to be a hydride; instead, the residual electron density observed in the middle of the cavity suggests the intriguing possibility of [**3-Mg**_**4**_]^−^ being an electride. Electrides are materials that hold a free electron in a cavity formed by cations.^25,36^ Inorganic electrides such as [Ca_24_Al_28_O_64_]^4+^4e^–^ have been shown to be room-temperature-stable and possess intriguing electronic properties such as high conductivity, and have even demonstrated applications as aqueous compatible reductants.^36−38^ However, to the best of our knowledge, only eight organic electrides have been synthesized, of which only one is room-temperature-stable.^39,40^ Remarkably, QTAIM reveals the presence of a non-nuclear attractor (NNA) with a charge of -0.48 in the center of [**3-Mg**_**4**_]^−^, thus strongly advocating the notion that the latter is an electride.^41^ The electride electron is topologically encaged by the interaction of 6,6′-hydrogen atoms of the bipyridine core within a capsule of an approximate length of 0.4 nm (Figure 3), similar to previously known organic electrides.^32^ The NNA appears only in the α-electron density and coincides with both the maximum spin density and the orbital HOMO-4, which we identify to be the SOMO of [**3-Mg**_**4**_]^−^, thus indicating SOMO–HOMO inversion.^15,42^ Evidence in favor of the true electride nature of [**3-Mg**_**4**_]^−^ is the negligible electron delocalization between the NNA and the nitrogen or magnesium atoms that is less than 0.05. A low delocalization index between the NNA and the surrounding atoms signifies the dominance of electrostatic interactions between the free electron and the positive Mg(II) centers akin to the ionic compounds.^43^

Taking all of these observations into consideration, we believe our available X-ray, EPR, DFT, and QTAIM data provide compelling evidence that [**3-Mg**_**4**_]^−^ is a room-temperature-stable electride composed of [**3-Mg**_**4**_] with a genuine free electron captured at its center. An inspection into the literature data indicates that many organic electrides have taken inspiration from pioneering work by Dye,^40,44^ which have found tremendous success by reacting chelating oxygen or nitrogen-donor ligands such as aza-crown ethers with alkali metals to access a range of organic electrides such as K^+^(cryptand[2.2.2])e^–^, Cs^+^(18-crown-6)_2_e^–^ or Na^+^(TriPip222)e^–^.^45^ However, these alkali cation–nitrogen systems form relatively weak bonds, implying that most known organic electrides decompose at or below room temperature.^32^ In sharp contrast, [**3-Mg**_**4**_]^−^ has strong Mg(II)–(bipy^2–^) linkages and four Mg(II)–*e*^–^ interactions that stabilize the structure. To the best of our knowledge, [**3-Mg**_**4**_]^−^ is the first experimentally characterized Mg electride^46^ as well as the first known example employing bipyridine as a stabilizing ligand in an electride core.^36^ The alternative possibility of [**3-Mg**_**4**_]^−^ existing as an aromatic tetranuclear core was also raised in analogy with the aforementioned Th_3_ complex.^30,31^ However, we believe the absence of Mg–Mg bonds and the fact that the molecule has only one electron does not follow the 4n + 2 Hückel rule, which argue against a possible aromaticity of [**3-Mg**_**4**_]^−^.

In light of these results, we believe **3** complements existing reports of bipyridine dianions with highly reducing alkali metals,^19,20^ while offering (a) access to a new room-temperature-stable electride, (b) new considerations into the redox chemistry of bipyridine ligands, (c) potential new applications of **3** as a mild, homogeneous reductant, and (d) the formation of macrostructures that group 1 analogues are not suited to.^47^ In addition, the identification of **3** from the direct reduction of bipyridine-ligated nickel species demonstrates their susceptibility to participate in electron transfer processes. This observation is particularly important, tacitly suggesting that care should be taken when generalizing existing reactivity found in the Ni-catalyzed arena, particularly within the context of catalytic reductive couplings that utilize either strong metallic reductants or homogeneous photocatalysts.^3,4,12^

**Figure 4:**
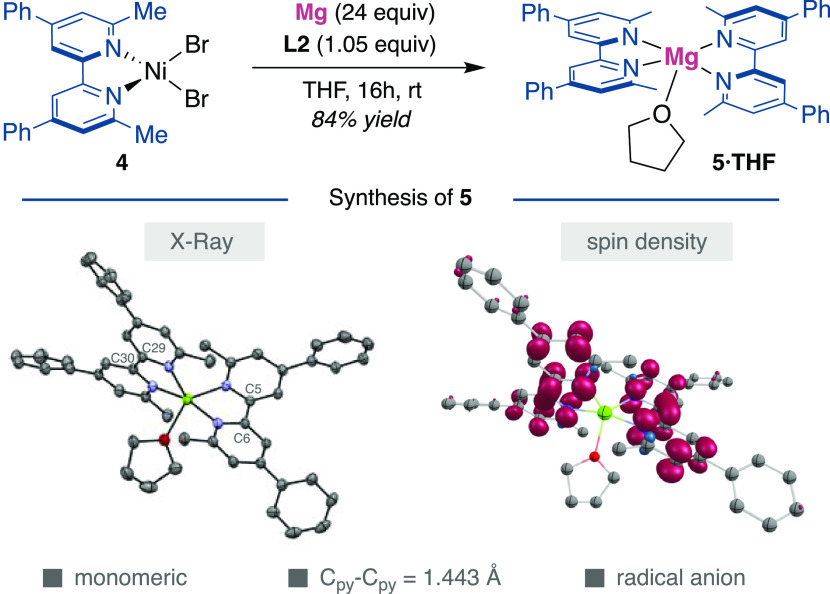
Reduction of sterically encumbered ligand. Synthesis, DFT spin density, and X-ray structure of **5** with thermal ellipsoids drawn at the 50% probability level. Selected distances (Å) C_py_–C_py_; C5–C6 1.443(3), C29–C30 1.442(3).

Taking into consideration the influence exerted by sterically encumbered 2,2-bipyridine ligands on reactivity,^48^ we turned our attention to investigating the generality of accessing reduced polypyridine-Mg species other than **3**, as it might pave the way for future synthetic applications. To this end, an otherwise similar route to that shown for **3** was followed with more sterically encumbered **L2** (6,6′-dimethyl-4,4′-diphenyl-2,2′-bipyridine), using (**L2**)NiBr_2_ (**4**) as a precursor (Figure 4). Gratifyingly, we were able to isolate a moisture- and oxygen-sensitive black powder, which was unequivocally characterized by X-ray diffraction as the monomeric structure **5·THF**. The divergent structure of bis-ligated, monomeric magnesium complex **5**, compared to **3**, reinforces the modularity exerted by polypyridine ligands, the generality of ligand reduction, and the unique reactivity of **3** to stabilize a free electron within its molecular structure. A comparison of the C_py_–C_py_ bond length of **L2**(49) and **5·THF** reveals a small contraction in the latter (1.496(3) vs 1.443(3) Å), suggesting that each of the two bipyridine ligands in **5** bears one electron as a radical anion, bound to a Mg(II) center. This interpretation gains credence by observing an EPR signal at *g* = 2.00296, with DFT calculations supporting a biradical electronic state, with one unpaired electron on each bipyridine unit (Figure 4). While the preferred electronic state for **5** is a triplet, our calculations indicate that the open-shell singlet is only slightly higher in energy,^15^ suggesting that **5** may behave as a spin crossover complex.

The rapid, reliable, and ease of synthesis of **3** and **5**, together with the wide range of redox potentials that could be accessed by fine-tuning the substituents on the bipyridyl core augurs well for their utilization as homogeneous reductants.^50^ Aimed at unraveling the potential of these complexes, we benchmarked their ease of handling and tunable reactivity by accessing low-valent (bipy)_2_Ni(0) complexes, compounds of utmost mechanistic relevance in Ni-catalyzed reactions.^3,4,12,51^ Unlike their *ortho*-substituted 2,2′-bipyridyl analogues,^52^ the synthesis of (bipy)_2_Ni(0) (**2**) requires challenging experimental setups such as metal vapor synthesis,^16^ or heterogeneous reductants such as Li metal,^5^ which suffer from competing overreduction, poor scalability, and irreproducibility.^27,50^ While one might argue that the means to access (bipy)_2_Ni(0) does not offer a clear advantage compared to the utilization of Li metal, we believe that the use of **3** may offer preparative advantages such as the ability to be stored in a glovebox, being a readily weighed powder, and being highly soluble in common organic solvents such as THF. In addition, the utilization of **3** likely forms insoluble Mg salts post oxidation, which would all aid in reaction setup and workup. Attempting to access **2** from ligand exchange or the use of other less reducing heterogeneous reductants were unsuccessful with traces, if any, of **2** being observed by exposing Ni(COD)_2_ to bipy^53^ or by reaction of **1** with Mn, Zn, or Mg (Scheme 4, left).^15^ Such lack of reactivity can tentatively be attributed to both slow electron transfer rates^14^ and the deleterious impact that inorganic salts formed by post-reduction (MCl_2_; M = Mn, Zn, Mg) might have on the reaction outcome.

**Scheme 3:**
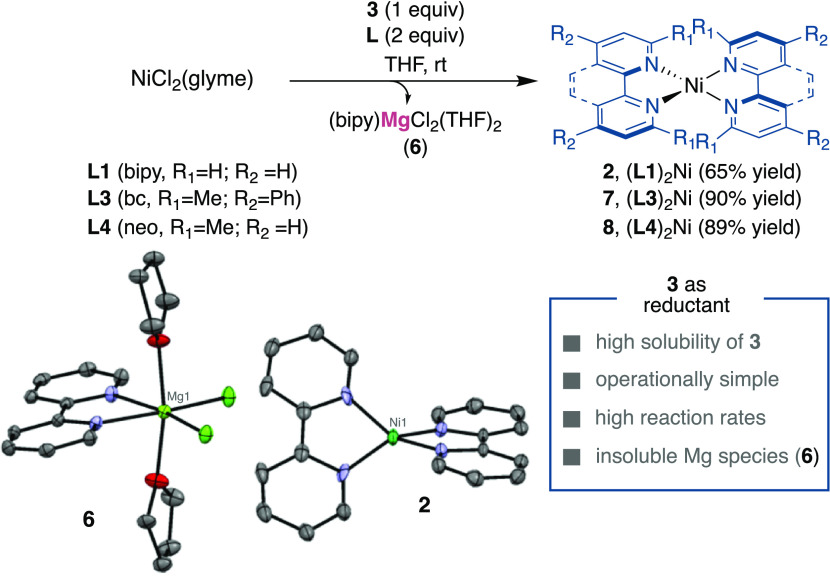
Homoleptic Ni(0) Polypyridine Complexes

**Scheme 4:**
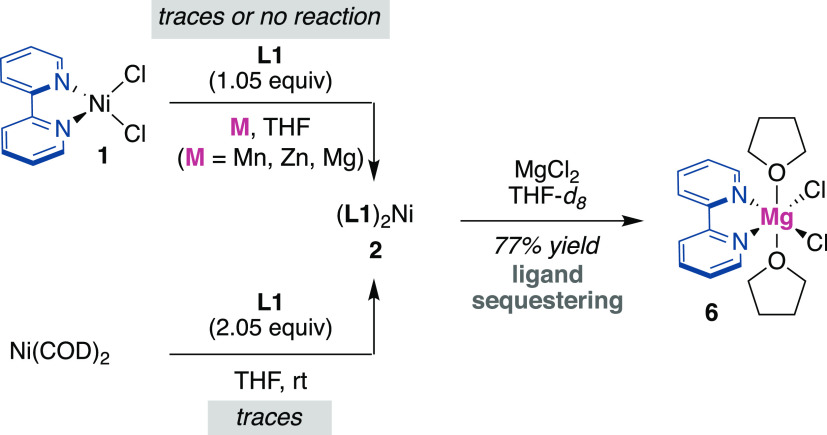
Failed Routes toward **2** and Ligand Sequestering

Gratifyingly, the utilization of **3** as homogeneous reductant cleanly delivered (bipy)_2_Ni(0) in 65% yield after 1 h reaction time, together with the formation of insoluble (bipy)MgCl_2_(THF)_2_ (**6**), the structure of which was confirmed by X-ray diffraction (Scheme 3).^15,54^ It is worth noting that this reaction proceeds in <1 h, thus representing an added value compared to the utilization of heterogeneous Li metal to access (bipy)_2_Ni that requires 24 h. Notably, ligands other than bipy could be employed with equal ease, as **7** or **8** was easily within reach from NiCl_2_(glyme) and **L3** or **L4** (**L3** = bathocuproine, **L4** = neocuproine) with **3**.^55^ Our hypothesis that MgCl_2_ salts formed using heterogeneous Mg as a reductant would be deleterious was indirectly confirmed by reacting **2** with MgCl_2_, leading to rapid decomposition of the former and formation of **6** (Scheme 4, right). These findings highlight the importance of forming highly coordinated and insoluble Mg complexes of type **6** en route to low-valent Ni(0) complexes, thus avoiding parasitic ligand sequestering events.

## Conclusions

In summary, we have synthesized and isolated unorthodox Mg complexes that do not only represent the first example of group 2 metal reduction to bipyridine dianions and an unprecedented room-temperature-stable electride stabilized by neighboring magnesium cores but also offer new opportunities for accessing elusive metal intermediates that were otherwise inaccessible by operationally simple techniques. We have additionally demonstrated the importance of ligand sequestering events in decomposition pathways, and solutions to overcome these limitations.

## Supporting Information Available

Cartesian coordinates for all structures included in the paper (XYZ)
Experimental procedures and spectral, crystallographic, and computational data (PDF)

## Supplementary Material

ja2c01807_si_001.xyz
