# Supplementary material for: Room-Temperature-Stable Magnesium Electride via Ni(II) Reduction
Source: J Am Chem Soc. 2022 Jul 13;144(29):13109–17. doi: 10.1021/jacs.2c01807 (PMC9345648; doi:10.1021/jacs.2c01807)
Supplement: Supplementary file 2 — ja2c01807_si_003.pdf [file ja2c01807_si_003.pdf]

Supporting Information

A Room Temperature-Stable Magnesium Electride via Ni(II) Reduction

Craig S. Day[†¶], Cuong Dat Do[#¤&], Carlota Odena[†¶&], Jordi Benet-Buchholz[†], Liang Xu[†], Cina Foroutan-Nejad[*$], Kathrin H. Hopmann[*¤] and Ruben Martin[*†§]

[†] Institute of Chemical Research of Catalonia (ICIQ), The Barcelona Institute of Science and Technology, Av. Països Catalans 16, 43007 Tarragona, Spain

[¶] Departament de Química Analítica i Química Orgànica, Universitat Rovira i Virgili, c/Marcel·lí Domingo, 1, 43007 Tarragona, Spain

[#] Hylleraas Center for Quantum Molecular Sciences and [¤]Department of Chemistry, UiT The Arctic University of Norway, N-9037 Tromsø, Norway

[$] Institute of Organic Chemistry, Polish Academy of Sciences, Kasprzaka 44/52, 01-224, Warsaw, Poland.

[§] ICREA, Passeig Lluís Companys, 23, 08010 Barcelona, Spain
Corresponding authors: rmartinromo@iciq.es, cforoutan-nejad@icho.edu.pl, kathrin.hopmann@uit.no

Table of Contents

S1. General Considerations **2**
S2. Synthesis and Characterization of Complexes **3**
S3. Stochiometric Reactions **5**
Synthesis of (bipy)$_2$Ni 2 with 3 .... *5*
Synthesis of (bipy)$_2$Ni 2 from KC_8 Reduction *6*
Synthesis of (bipy)Ni(COD) from Ni(COD)$_2$ *7*
Attempted Synthesis of (bipy)$_2$Ni 2 with Mg, Mn, or Zn *8*
Synthesis of (Bathocuproine)$_2$Ni *9*
Synthesis of (Neocuproine)$_2$Ni *9*
Direct Reduction of Bipyridine, Neocuproine, and Bathocuproine With Magnesium *9*
In-Situ Synthesis of (Bathocuproine)$_2$Ni *10*
Reaction of (bipy)$_2$Ni 2 with $MgCl_2$ *11*
Independent formation of (bipy)$MgCl_2$ *12*
Pinacol coupling of benzaldehyde with 3 .... *13*
Synthesis of 3 with THF-d_8 .... *14*
Independent formation of radical anion (bipy)Mg *15*
S4. IR, UV-VIS, and Cyclic Voltammetry **16**
Infrared Spectroscopy of 3 and 5 .... *16*
UV-VIS Spectroscopy of 3 and 5 .... *17*
Cyclic Voltammetry of 1, 3 and 5 .... *18*
S5. NMR and EPR Spectra of Synthesized Complexes **20**
S6. Crystallographic Data **26**
S7. Computational details **35**
S8. References **49**

S1. General Considerations

Solvents. Reactions were carried out under N_2 in a glovebox or on a Schlenk line, in solvents (THF, Et_2O, toluene) that had been dried and degassed using an Innovative Technologies solvent purification system, then stored under N_2 over 4 Å molecular sieves for at least 16 h prior to use. Pentane was degassed by bubbling with N_2 and stored under N_2 over 4 Å molecular sieves for at least 16 h prior to use. C_6D_6, C_7D_8, CD_2Cl_2, THF-d_8, (Eurisotop) were freeze/pump/thaw degassed (4x) and likewise stored under N_2 over 4 Å molecular sieves for at least 16 h prior to use.

Reagents. Bipyridine and bathocuproine were purchased from Fluorochem. Magnesium powder (99 ≥%) and $MgCl_2$ (98 ≥%) were purchased from Sigma-Aldrich. Magnesium powder (99.8 %) was purchased from Strem Chemicals. Trimethoxylbenzene (TMB) was purchased from TCI Chemicals. Complexes (bipy)$NiCl_2$,[1] (Bathocuproine)$NiCl_2$,[2] (Bathocuproine)$_2$Ni[2] and ligand 6,6'-dimethyl-4,4'-diphenyl-2,2'-bipyridine[3] were synthesized according to literature procedures.

Analytical methods. Flash chromatography was performed with Sigma Aldrich technical grade silica gel 60 (230-400 mesh). Thin layer chromatography was carried out using Merck TLC Silica gel 60 F254. NMR spectra were recorded on Bruker Avance Ultrashield 300, 400, or 500 MHz spectrometers, with chemical shifts reported in parts per million (ppm) and coupling constants, *J*, reported in hertz. Quantitative NMR experiments were performed with d1 set to 10s (^{1}H). Gas chromatographic analyses were performed on an Agilent 6890N gas chromatograph with an FID detector. Continuous wave (CW) X-band EPR spectra were obtained using a Bruker EMX Micro X-band spectrometer using a Bruker ER 1164 HS resonator. Spectra were simulated using SpinFit within Xenon. The samples were cooled to 77 K in a Suprasil finger dewar (Wilmad-LabGlass) filled with liquid nitrogen. The spectral data were collected with the following spectrometer settings: microwave power = 0.56mW; centre field = 3250 G, sweep width = 2500 G, sweep time = 35.07 s, modulation frequency = 100 KHz, modulation amplitude = 10 G, power attenuation= 25 dB, time constant = 20.48 ms. Simulations, g values, and frequencies are provided alongside the characterization data of the complexes. IR spectra were obtained with a Bruker FT-IR Alpha spectrometer. Elemental analysis and powder diffraction were unsuccessful in characterizing new Mg complexes in this work due to the reactivity and highly reduced nature of the species synthesized.

S2. Synthesis and Characterization of Complexes

Synthesis of $[(THF)_4Mg_4(\mu^2\text{-bipy})_4][(THF)_6Mg_2(\mu^2\text{-bipy})(Cl)]$ 3

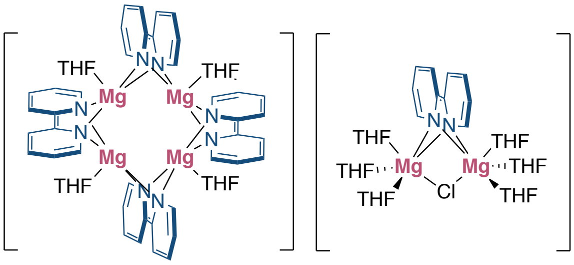

3-Mg₄ Tetranuclear **3-Mg₂** Dinuclear

In the glovebox, (bipy)$NiCl_2$ (1.028 g, 3.62 mmol) was added to a 100 mL Schlenk flask with bipyridine (0.596 g, 3.82 mmol) and Mg powder (2.115g, 87.02 mmol). A stir bar was added, and the Schlenk flask was charged with 30 mL of THF turning the teal powder to a teal suspension and let stir overnight. After 16 hours the purple solution was filtered through a celite plug with a black solid and unreacted magnesium being filtered off and a purple solution collected. The solvent was then removed to afford a purple solid and washed with pentane (15 mL x 3) to give $[(THF)_4Mg_4(\mu^2\text{-bipy})_4][(THF)_6Mg_2(\mu^2\text{-bipy})(Cl)]$ **3** (2.457 g, 98 % yield) as a purple powder.

Note: Yield. Based on bipyridine. **Mg source.** We found that using Mg powder (99 ≥%) was important for faster reaction times (those noted) instead of Mg powder (99.8 %). **Stir rate.** A fast-stirring velocity was found to improve reaction times and mixing of the heterogeneous reductant. In the case of a slower reaction times/reduction, the reactions could be left longer with no loss in yield. **Incomplete reduction.** Syntheses conducted with shorter reaction times (ca. 4 h) showed incomplete reduction to **3** and crystals of $Mg(bipy)_2(THF)_2$ (See Fig 31 for X-Ray structure) formed. We were unable to isolate this structure from **3** under these short reaction times, but analogous reports from the reduction of bipyridine to the radical anion directly with magnesium are described.[4] **Stoichiometry of Magnesium.** Likely lower stoichiometries of magnesium can be used if longer reaction times are employed. However, for a convenient and reproducible procedure an excess of magnesium is used. **Temperature.** While performing variable temperature ^{1}H NMR experiments (from rt to 55 °C – Figure S17) no decomposition was observed. **Stability.** While **3** shows prolonged stability as a solid stored in the glovebox at room temperature, the complex reacts readily with oxygen or moisture so it must be handled under inert conditions.

^{1}H NMR (400 MHz, THF-d_8): δ 6.55 (d, J = 6.8 Hz, 1H), 5.60 (d, J = 9.4 Hz, 1H), 4.77 (dd, J = 9.4, 5.2 Hz, 1H), 4.10 (t, J = 5.9 Hz, 1H). **^{13}C NMR** (101 MHz, THF-d_8): δ 149.3, 148.7, 148.6, 124.5, 124.4, 123.3, 119.7, 119.4, 119.0, 114.7, 114.3, 97.8, 97.4.

EPR = 2.00365 g (strong)

Evan's Method, μ_{eff} = 1.80 µB

UV-VIS (nm) = 915, 813, 725, 530, 501, 383, 373

Key IR Stretches = 1557 cm^{-1} (C=C and C=N), 954 cm^{-1} (ring deformation stretch)

See Figure S10 for full IR Spectra

(6,6'-dimethyl-4,4'-diphenyl-2,2'-bipyridine)$NiBr_2$ 4

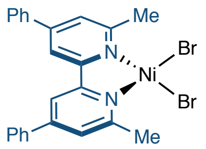

In the glovebox, $NiBr_2$diglyme (308 mg, 0.87 mmol) and 6,6'-dimethyl-4,4'-diphenyl-2,2'-bipyridine (301 mg, 0.90 mmol, 1.025 equiv.) were added to a 10 mL vial. A stir bar was added and charged with 6 mL of THF and left to stir overnight. After 16 h pentane (5 mL) was added to the suspension and then was filtered. The solid was washed with pentane (1 mL x 3) to afford (6,6'-dimethyl-4,4'-diphenyl-2,2'-bipyridine)$NiBr_2$ **4** as a pink powder (472 mg, 97 % yield).

^{1}H NMR (400 MHz, CD_2Cl_2): δ 74.3 (br s, 2H), 60.5 (br s, 2H), 20.2 (br s, 7H).

EA Calcd. C, 51.95; H, 3.63; N, 5.05; Found: C, 48.89; H, 3.83; N, 4.73 The deviation from the calculated EA values might be due to incomplete sample combustion and the formation of Ni black on the glass reaction tube during EA analysis. Alternatively, deviation may arise from contaminant during shipping or residual solvent or ligand.

Mg(6,6'-dimethyl-4,4'-diphenyl-2,2'-bipyridine)$_2$(THF) 5

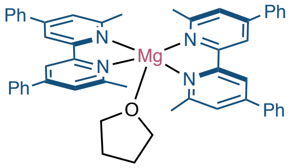

In the glovebox, (6,6'-dimethyl-4,4'-diphenyl-2,2'-bipyridine)$NiBr_2$ (145 mg, 0.26 mmol) was added to a 10 mL vial with 6,6'-dimethyl-4,4'-diphenyl-2,2'-bipyridine (93 mg, 0.28 mmol) and Mg powder (153 mg, 6.29 mmol). A stir bar was added, and the vial was charged with 4 mL of THF turning the powder into a suspension and let stir overnight. After 16 h the solution was filtered through a celite plug with a black solid being filtered off and a dark brown solution was collected. The solvent was removed to afford a black solid and washed with pentane (4 mL x 4) to give Mg(6,6'-dimethyl-4,4'-diphenyl-2,2'-bipyridine)$_2$(THF) **5** (169 mg, 84 % yield) as a black powder.

^{1}H NMR (500 MHz, THF-d_8, weak): δ 7.42 (d, J = 7.8 Hz, 5H), 7.23 (d, J = 7.9 Hz, 5H), 7.14 (t, J = 7.5 Hz, 7H), 7.05 (t, J = 7.6 Hz, 6H), 6.96 (t, J = 7.4 Hz, 3H), 6.81 (t, J = 7.3 Hz, 3H), 6.53 (s, 3H), 6.28 (s, 3H), 5.06 (s, 3H), 4.80 (s, 4H). The NMR samples were prepared from a saturated solution of **5** in THF-d_8 to improve signal intensity. However, due to the mainly paramagnetic nature of **5** and the resulting low intensity of the NMR signals obtained, no ^{13}C NMR signals were observed.

EPR = 2.00296 g (weak)

Evan's Method, μ_{eff} = 3.06 µB

UV-VIS (nm) = 486, 406, 372

Note: Samples were unstable under UV-VIS conditions, where after one set of scans decomposition was observed (see Figure S11).

Key IR Stretches = 1573 cm^{-1} (C=C and C=N), 992 cm^{-1} (ring deformation stretch)

See Figure S10 for full IR Spectra.

S3. Stochiometric Reactions

Synthesis of $(bipy)_2Ni$ 2 with 3

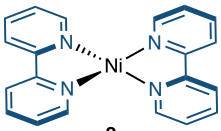

2

In the glovebox, $NiCl_2$(glyme) (64 mg, 0.29 mmol) and bipyridine (91 mg, 0.58 mmol) were added to a 12 mL vial. A stir bar was added, and the vial was charged with 2 mL of THF forming a teal suspension. After 10 minutes, a solution of **3** (89 mg, 0.58 mmol) in 2 mL THF was added dropwise. After 55 minutes the green suspension was filtered through a celite plug with a black solid being filtered off and a green solution collected. The solvent was removed to afford a green solid and washed with pentane (0.5 mL x 2) to give $(bipy)_2Ni$ **2** (70 mg, 65 % yield) as a green powder.

^{1}H NMR (400 MHz, C_6D_6): δ 10.20 (d, J = 5.7 Hz, 2H), 7.72 (ddd, J = 8.1, 6.8, 1.4 Hz, 2H), 7.24 (d, J = 8.5 Hz, 4H). **^{13}C NMR** (101 MHz, C_6D_6): δ 149.4, 140.4, 125.0, 121.1, 120.2.

Notes: Stoichiometry of **3** is determined by overall MW/# of reducing electrons (i.e 1683,29/11 = 153.0 g/mol per reducing equivalent) multiplied by 2 as two electrons are required to reduce Ni(II) to Ni(0). Crystals of **6** (see Figure S30) were formed from the crude reaction mixture. **Literature syntheses**. The syntheses of $(bipy)_2Ni$ **2** have been reported from metal-vapor synthesis,[5] lithium metal reduction[6] and through in situ or bulk electrochemical reductions.[1] A common theme in the synthesis of this complex are challenging experimental setups. Use of heterogeneous Li or KC_8 reductants (see below for synthesis using KC_8) can result in surface-retained over-reduction which led to irreproducible results that suffer from poor scalability. Furthermore, reductions with Li cannot be performed in typical N_2 atmosphere gloveboxes due to the exothermic reaction of Li with N_2 and its low molecular weight complicates its use at small scales. In the reported lithium metal reduction, 14 mg of lithium metal are weighed to perform a stochiometric reduction at 1 mmol scale. The heterogeneous nature also results in longer reaction times with poor control over reaction rates. Alternatively, less well-defined procedures have been described from $K_4[Ni(CN)_4]$,[7] $Ni(COD)_2$,[8] and $(bipy)NiEt_2$.[9] In our hands, synthesis from $Ni(COD)_2$ were unsuccessful forming (bipy)Ni(COD) (see synthesis below) and $(bipy)NiEt_2$ resulted in no reaction, likely contributes to the general lack of literature reports implementing $(bipy)_2Ni$. Commonly, literature investigations of bipy-Ni complexes rely on in situ generation of (bipy)Ni(COD) from $Ni(COD)_2$. These investigations however generate a mixture of species[10] between $Ni(COD)_2$, (bipy)Ni(COD)[11] and $(bipy)_2Ni$, where the interactions of COD may complicate the desired outcome. This mixture of speciation also complicates isolation of $(bipy)_2Ni$ in pure form from $Ni(COD)_2$. **NMR Characterization.** To our knowledge only ^{1}H NMR characterization has been provided by Bartak[5] which is similar to those reported above. Fuller characterization is now reported with both ^{1}H and ^{13}C NMR data and corresponding spectra, in addition to unambiguous assignment by single crystal XRD.

Synthesis of $(bipy)_2Ni$ 2 from KC_8 Reduction

In the glovebox, $(bipy)NiCl_2$ (25.2 mg, 0.09 mmol), bipyridine (13.9 mg, 0.09 mmol), and KC_8 (13.5 mg, 0.10 mmol) were added to a 12 mL vial. A stir bar was added, and the vial was charged with 2 mL of THF turning the teal powder to a teal suspension. The initial dark pink solution gradually turned dark green over few minutes. (**Note.** Two identical reactions were carried out to be analyzed in different reaction times.) After 55 min and 3 hours, the green suspension was filtered through a celite plug with a black solid being filtered off and a green solution collected. The solvent was removed to afford a green solid and washed with pentane (0.15 mL x 2). **Note.** Other experimental setups by adding KC_8 as a dropwise slurry in THF were unsuccessful.

- <u>After 55 min:</u> ^1H NMR analysis (C_6D_6) identified a mixture of $(bipy)_2Ni$ **2** and unreacted bipyridine (54:46 ratio – see Figure S1).
- <u>After 3 h:</u> $(bipy)_2Ni$ **2** (crude; 13.0 mg, 39 % yield – 10:1 ratio $(bipy)_2Ni$:free bipy) was obtained as a green powder. Significant spectral broadening is observed due to free ligand in equilibria with $(bipy)_2Ni$ (see Figure S1).

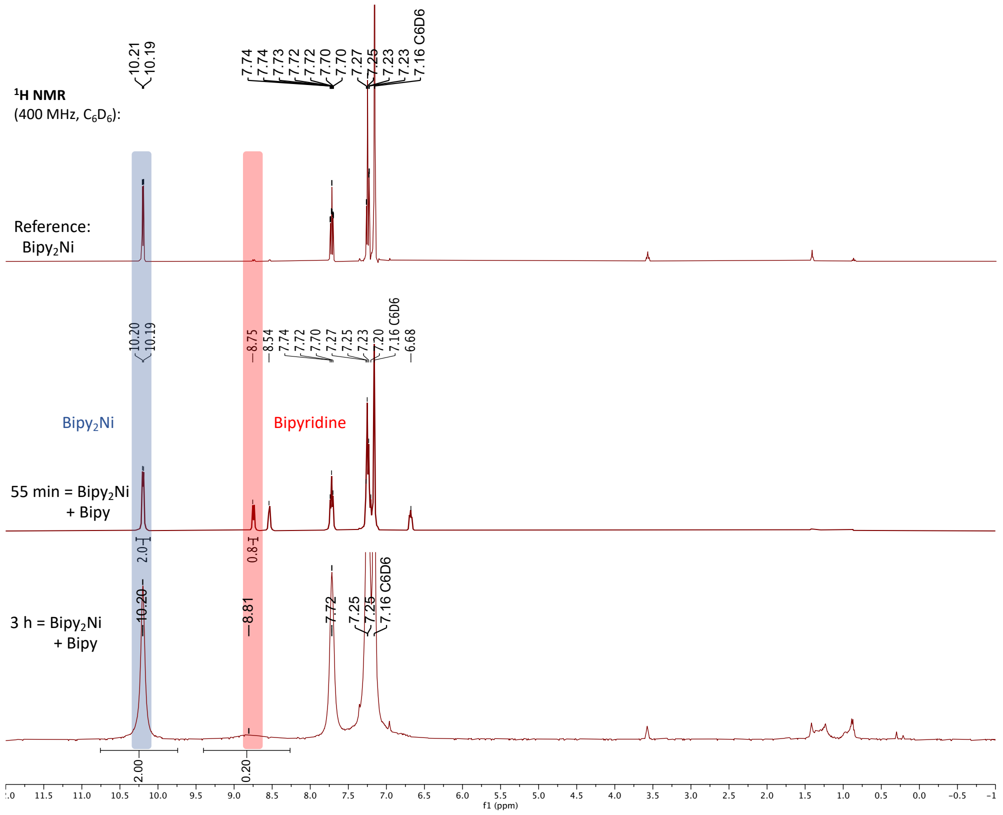

Figure S1. ^1H spectra (C_6D_6, 400 MHz) reducing $(bipy)NiCl_2$ with KC_8 (1.1 equiv.). After 55 min, a mixture of $(bipy)_2Ni$ **2** (blue) and unreacted bipyridine (red) is observed (54:46 ratio).

Synthesis of (bipy)Ni(COD) from $Ni(COD)_2$

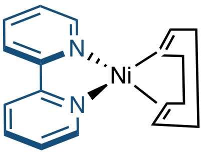

In the glovebox, $Ni(COD)_2$ (81 mg, 0.29 mmol) and bipyridine (96 mg, 0.62 mmol 2.1 equiv) were added to a 12 mL vial. A stir bar was added, and the vial was charged with 8 mL of pentane. After 16 h, the solution was filtered through a celite plug and the solvent was removed to afford a purple solid. The solid was washed with pentane (0.5 mL x 2) to give (bipy)Ni(COD) (75 mg, 92 % yield) as a purple powder with minor impurities of $(bipy)_2Ni$ **2** and COD.

^{1}H NMR (500 MHz, C_6D_6): δ 10.15 (d, J = 5.8 Hz, 2H), 7.29 (d, J = 3.9 Hz, 4H), 7.00 (d, J = 3.2 Hz, 2H), 3.92 (d, J = 3.4 Hz, 4H), 3.01 – 2.72 (m, 4H), 1.95 (d, J = 8.3 Hz, 4H). The spectroscopic data correspond to those previously reported in the literature.[11]

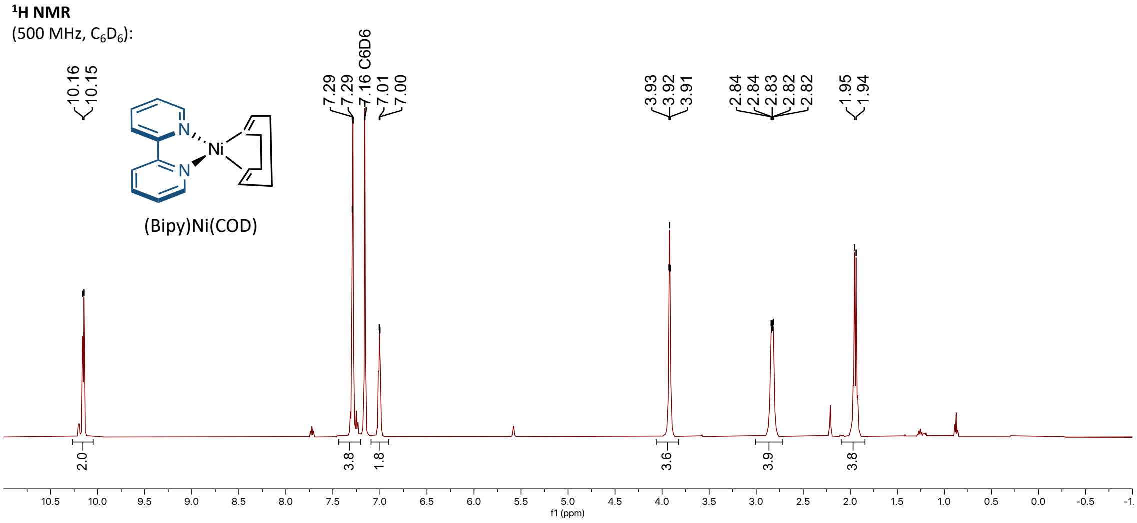

Figure S2. ^{1}H spectrum (C_6D_6, 500 MHz) of (bipy)Ni(COD) from $Ni(COD)_2$. bipy = bipyridine, COD = cyclooctadiene

Attempted Synthesis of $(bipy)_2Ni$ 2 with Mg, Mn, or Zn

In the glovebox, $(bipy)NiCl_2$ (25.2 mg, 0.09 mmol), bipyridine (13.9 mg, 0.09 mmol), and Mg powder (2.2 mg, 0.09 mmol) were added to a 12 mL vial. A stir bar was added, and the vial was charged with 2 mL of THF turning the teal powder to a teal suspension. The reaction remained a teal suspension that gradually began to darken over 7 days. After 7 days, an aliquot was taken from the suspension, filtered through a celite plug to remove unreacted $(bipy)NiCl_2$ and the solvent was removed. ^{1}H NMR analysis (C_6D_6) identified minor amounts of $(bipy)_2Ni$ **2**, and mainly unreacted bipyridine (15:85 ratio - below).

Note. Analogous Reactions with Mn or Zn afford no signals for $(bipy)_2Ni$.

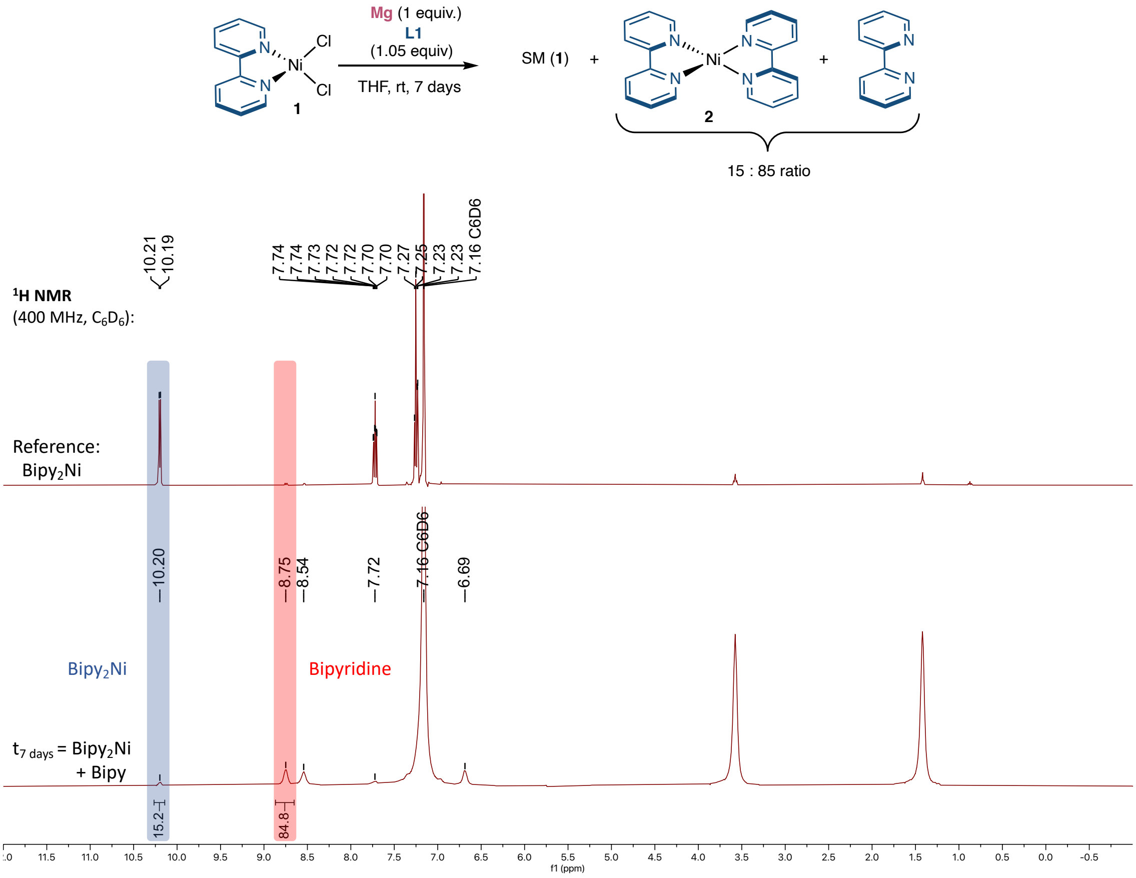

Figure S3. ^{1}H spectra (C_6D_6, 400 MHz) reducing $bipyNiCl_2$ with Mg powder (1 equiv.). After 7 days, very minor quantities of $(bipy)_2Ni$ **2** (blue) formed with largely unreacted bipyridine (red) remaining (15:85 ratio).

Synthesis of (Bathocuproine)$_2$Ni 7 with 3

In the glovebox, $NiCl_2$(glyme) (68 mg, 0.31 mmol) and bathocuproine (223 mg, 0.62 mmol) were added to a 12 mL vial. A stir bar was added, and the vial was charged with 4 mL of THF turning the powder to a suspension. After 5 minutes, a solution of **3** (95 mg, 0.62 mmol) in 4 mL THF was added dropwise. After 55 minutes the purple solution was filtered through a celite plug with a black solid being filtered off and a purple solution collected. The solvent was removed to afford a purple solid and washed with pentane (1 mL x 2) to give (Bathocuproine)$_2$Ni **7** (216 mg, 90 % yield) as a purple powder. The spectroscopic data correspond to those previously reported and validated by independent synthesis using literature procedures from Ni(COD)$_2$.[2]

^{1}H NMR (500 MHz, C_6D_6) δ 7.96 (s, 2H), 7.86 (s, 2H), 7.67 (dd, *J* = 8.2, 1.3 Hz, 4H), 7.43 – 7.33 (m, 2H), 7.24 – 7.17 (m, 4H, slight overlap with C_6D_6), 2.49 (s, 6H).
^{1}H NMR (400 MHz, THF-d_8): δ 8.11 (s, 2H), 7.90 – 7.70 (m, 6H), 7.62 – 7.48 (m, 2H), 7.40 (t, *J* = 7.6 Hz, 4H), 2.45 (s, 6H).

Synthesis of (Neocuproine)$_2$Ni 8 with 3

In the glovebox, $NiCl_2$(glyme) (68 mg, 0.31 mmol) and neocuproine (129 mg, 0.62 mmol) were added to a 12 mL vial. A stir bar was added, and the vial was charged with 4 mL of THF turning the powder to a suspension. After 5 minutes, a solution of **3** (95 mg, 0.62 mmol) in 4 mL THF was added dropwise. After 55 minutes the brown solution was filtered through a celite plug with a black solid being filtered off and a brown solution collected. The solvent was removed to afford a brown solid and washed with pentane (1 mL x 2) to give (neocuproine)$_2$Ni **8** (131 mg, 89 % yield) as a brown powder. The spectroscopic data correspond to those previously reported and validated by independent synthesis using literature procedures from Ni(COD)$_2$.[12]

^{1}H NMR (500 MHz, C_6D_6) δ 9.00 (d, *J* = 7.3 Hz, 2H), 7.68 (d, *J* = 7.3 Hz, 2H), 7.32 (s, 2H), 2.24 (s, 6H).

Direct Reduction of Bipyridine, Neocuproine, and Bathocuproine With Magnesium

An alternative synthesis to highly reduced polypyridine dianions could be envisioned via the direct reduction of the free ligand with magnesium. Attempts at the direct reduction of ligands bipyridine, neocuproine and bathocuproine with magnesium powder were attempted following the procedure for cluster **3**, however, none of the syntheses afforded the highly reduced ligand dianions of magnesium in high yields.

In-Situ Synthesis of (Bathocuproine)$_2$Ni 7

In the glovebox, bathocuproine (6.5 mg, 0.02 mmol) and trimethoxybenzene (1.8 mg, internal standard) were dissolved in 0.8 mL THF-d_8 in a 3 mL vial. The solution was then added to a J. Young NMR tube, and the initial integration ratio of bathocuproine and TMB was measured (below - t_0). The solution was brought back into the glovebox and added to a stirred solution of (bathocuproine)NiCl$_2$ (8.6 mg, 0.02 mmol) in 0.5 mL THF-d_8. **5** (16.6 mg, 0.02 mmol) was then added and a rapid color change to red was observed. After 30 minutes the solution was transferred into a J. Young NMR tube and analyzed by ^{1}H NMR spectroscopy (t_1 – Bc$_2$Ni, 27 % yield).

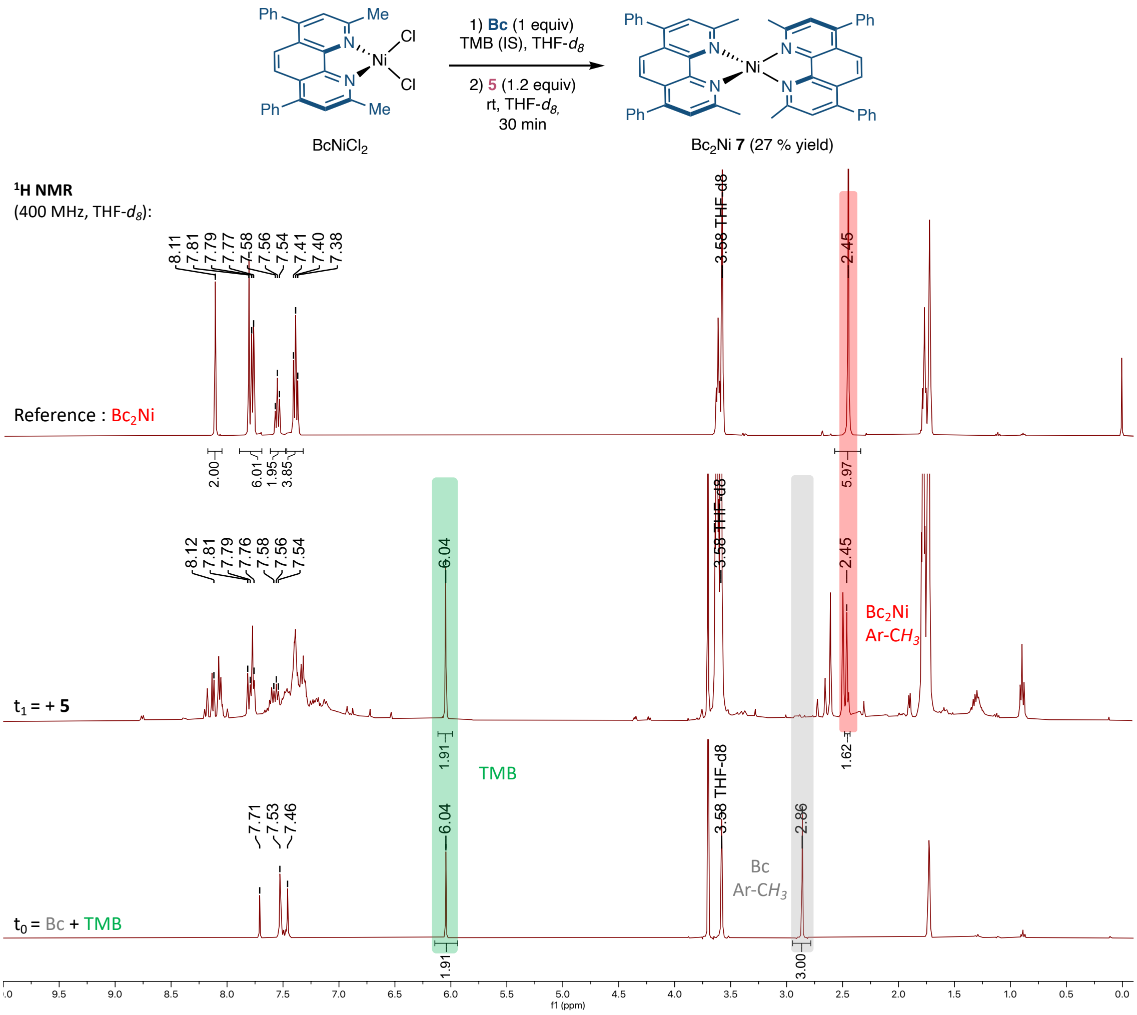

Figure S4. ^{1}H spectra (THF-d_8, 400 MHz) reducing (Bc)NiCl$_2$ to (Bc)$_2$Ni **7** (red) quantified from initial integration of bathocuproine (grey) using internal standard TMB (green).

Reaction of (bipy)$_2$Ni 2 with MgCl$_2$

In the glovebox, (bipy)$_2$Ni (9.8 mg, 0.03 mmol) and trimethoxybenzene (2.1 mg) were dissolved in 1 mL THF-d_8 in a 3 mL vial. The solution was then added to a J. Young NMR tube, and the initial integration ratio of (bipy)$_2$Ni and TMB was measured (below - t_0). The solution was brought back into the glovebox and added to a stirred suspension of MgCl$_2$ (5.5 mg, 0.06 mmol) in 0.5 mL THF-d_8. After 1 h a suspension was formed and filtered through a pipette plug of celite where a black solid was filtered of and the filtrate was collected into a J. Young NMR tube and measured by quantitative ^{1}H NMR (t_1 – only TMB remaining in filtrate). The filtrate was brought back into the glovebox and added to a 3 mL vial where the solvent was removed. DMF-d_7 was added to the precipitate noted earlier and passed through the same celite plug dissolving the previously filtered solid into the vial of TMB and transfer to a J. Young NMR tube and measured by quantitative ^{1}H NMR (t_2 – ratio of (bipy)MgCl$_2$(THF)$_2$ **6** formed as a precipitate and TMB measured, 77 % yield).

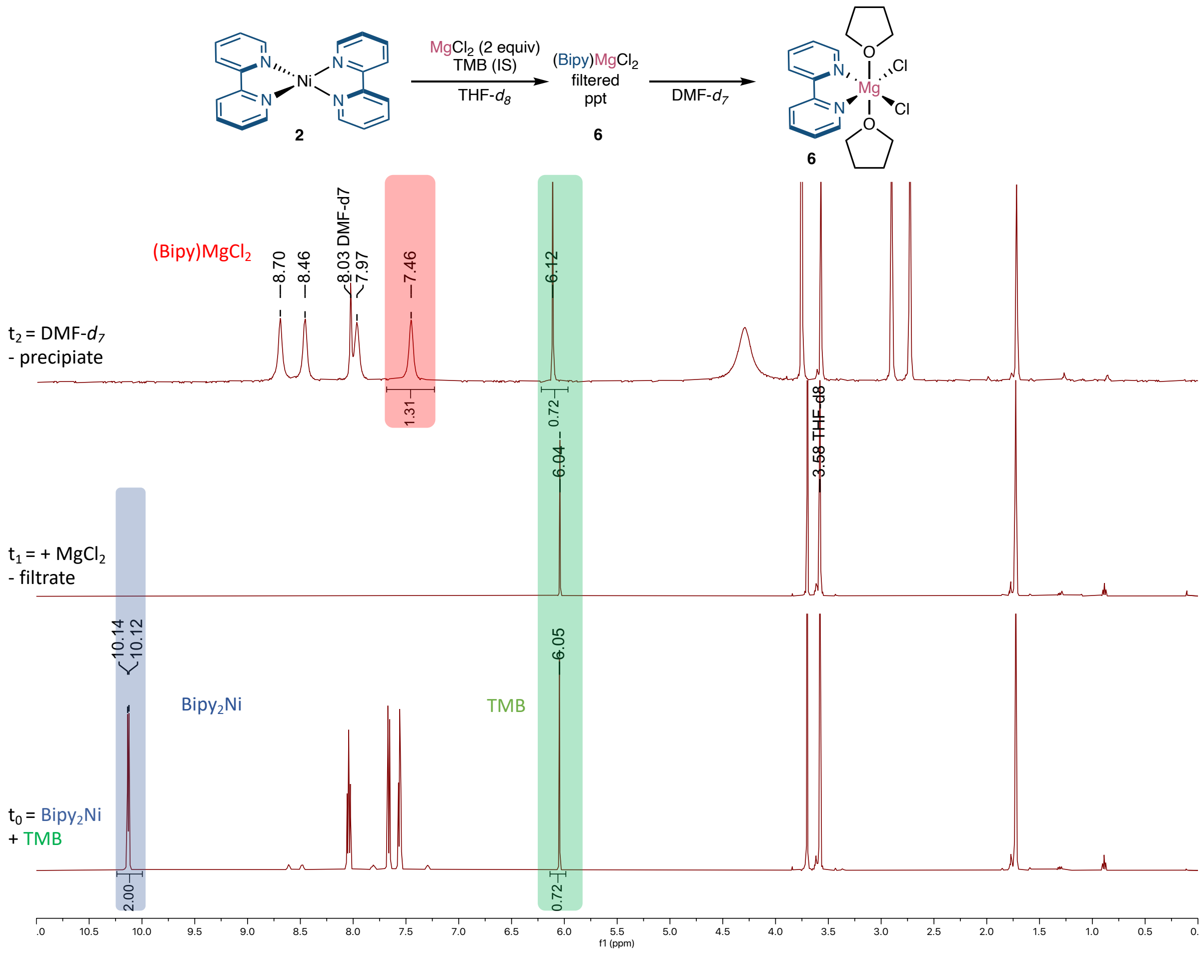

Figure S5. ^{1}H spectra (THF-d_8, 400 MHz and DMF-d_7) of MgCl$_2$ undergoing ligand scavenging of (bipy)$_2$Ni **2** (blue) forming (bipy)MgCl$_2$(THF)$_2$ **6** (red) using internal standard TMB (green).

Independent formation of (bipy)$MgCl_2$

$MgCl_2$ (5.8 mg, 0.06 mmol) and bipyridine (9.6 mg, 0.06 mmol) were added to a 4 mL vial with 1 mL DMF-d_7. The solids were then sonicated until the solution was homogeneous (ca. 5 minutes) and then analyzed by ^{1}H and ^{13}C NMR spectroscopy.

^{1}H NMR (400 MHz, DMF-d_7): δ 8.70 (br s, 2H), 8.45 (br s, 2H), 7.99 (br s, 2H), 7.47 (br s, 2H).

^{13}C NMR (101 MHz, DMF-d_7): δ 156.6, 138.2, 125.2, 121.5.

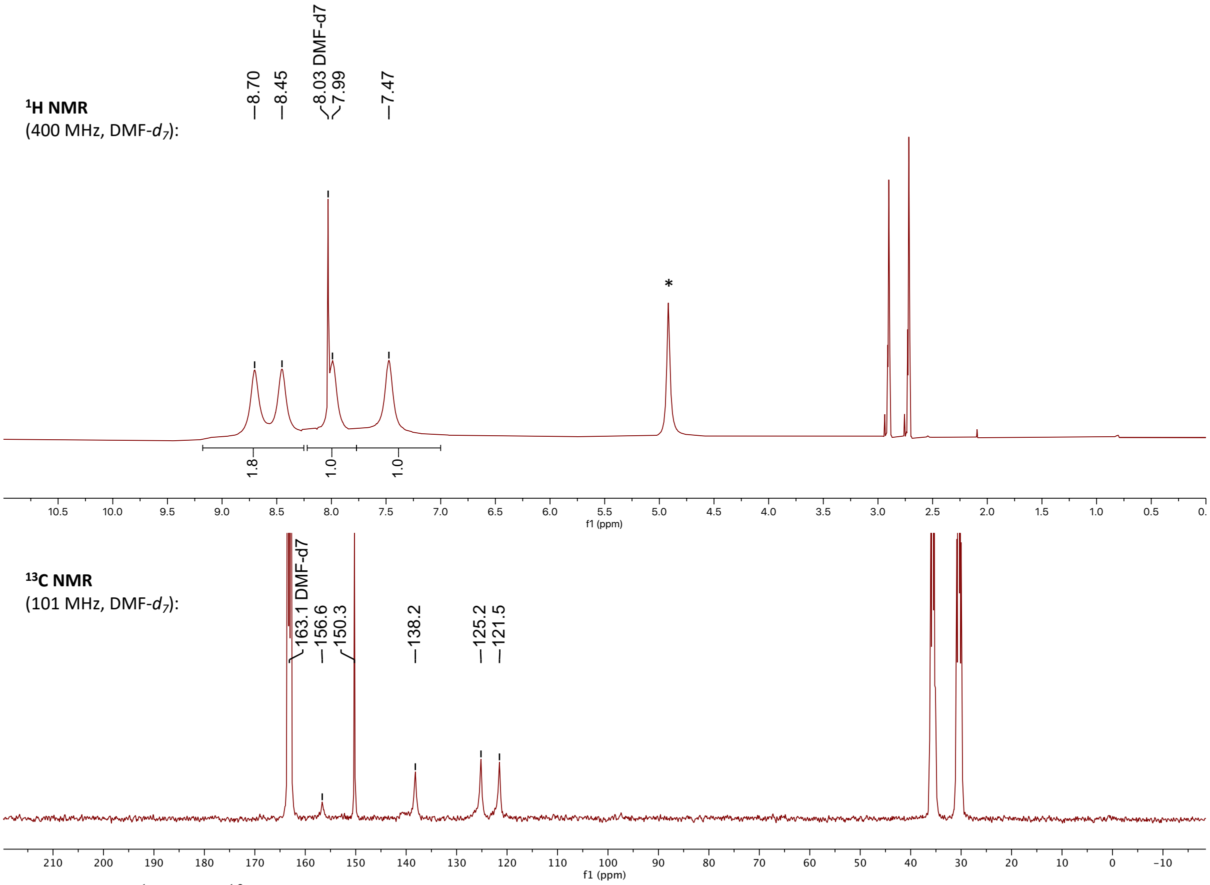

Figure S6. ^{1}H and ^{13}C spectra (DMF-d_7, 400/101 MHz) of (bipy)$MgCl_2$. *water

Pinacol coupling of benzaldehyde with 3

In the glovebox, **3** (8.4 mg, 0.5 mmol) and benzaldehyde (10.2 mg, 0.10 mmol) were added to a 12 mL vial. A stir bar was added, the vial was charged with 4 mL of THF and the reaction mixture was stirred at room temperature for two hours. After this time, the vial was taken out of the glovebox and trimethoxybenzene (6.5 mg, 0.04 mmol) was added as internal standard. Then, the reaction was quenched with aqueous HCl (1 M, 10 mL) and extracted with diethyl ether (15 mL x 3), organic phase was washed with saturated aqueous $NaHCO_3$ solution (10 mL) and brine (10 mL), dried over $MgSO_4$ and filtered. Solvent was removed under vacuum and the reaction mixture was solubilized in $CDCl_3$ and analysed by q^{1}H NMR spectroscopy which determined 1,2-diphenylethane-1,2-diol had formed in 94 % yield. The spectroscopic data corresponds to those previously reported in the literature.[13]

Note: The conversion of benzaldehyde was checked by TLC after one hour at room temperature showing no benzaldehyde remained. The reaction was left stirring 1 hour longer to ensure full conversion.

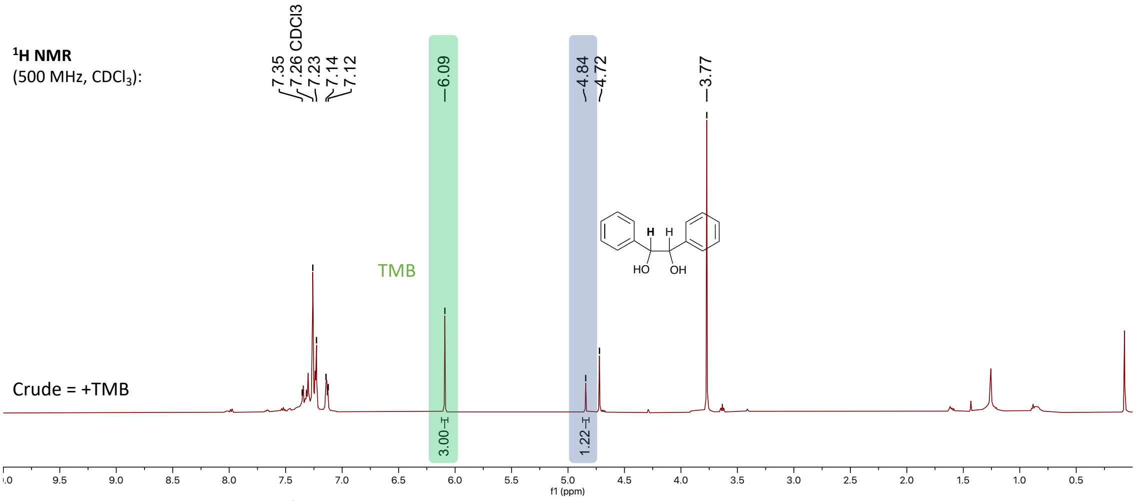

Figure S7. Quantitative ^{1}H NMR ($CDCl_3$, 500 MHz) spectrum of the crude reaction mixture upon workup with TMB added.

Synthesis of 3 with THF-d_8

In the glovebox, (bipy)$NiCl_2$ (34 mg, 0.12 mmol) was added to a 4 mL vial with bipyridine (20 mg, 0.13 mmol) and Mg powder (71 mg, 2.92 mmol). A stir bar was added, and the vial was charged with 1.5 mL of THF-d_8 turning the teal powder to a teal suspension and let stir overnight. After 16 hours the purple solution was filtered through a celite plug with a black solid and unreacted magnesium being filtered off and a purple solution collected. The solution was transferred to a J-young NMR tube and analyzed by ^{1}H and ^{2}H NMR spectroscopy. The signals observed in the synthesis using THF match those in THF-d_8 supporting they are the same species except for the difference in deuterated THF. Furthermore, there are no signals upfield ^{2}H NMR signals that would correspond to a deuteride. Minor differences in peak intensities corresponds to differences in the ^{1}H NMR sample concentration due to fluxionality as observed in the VT and EXSY ^{1}H NMR experiments.

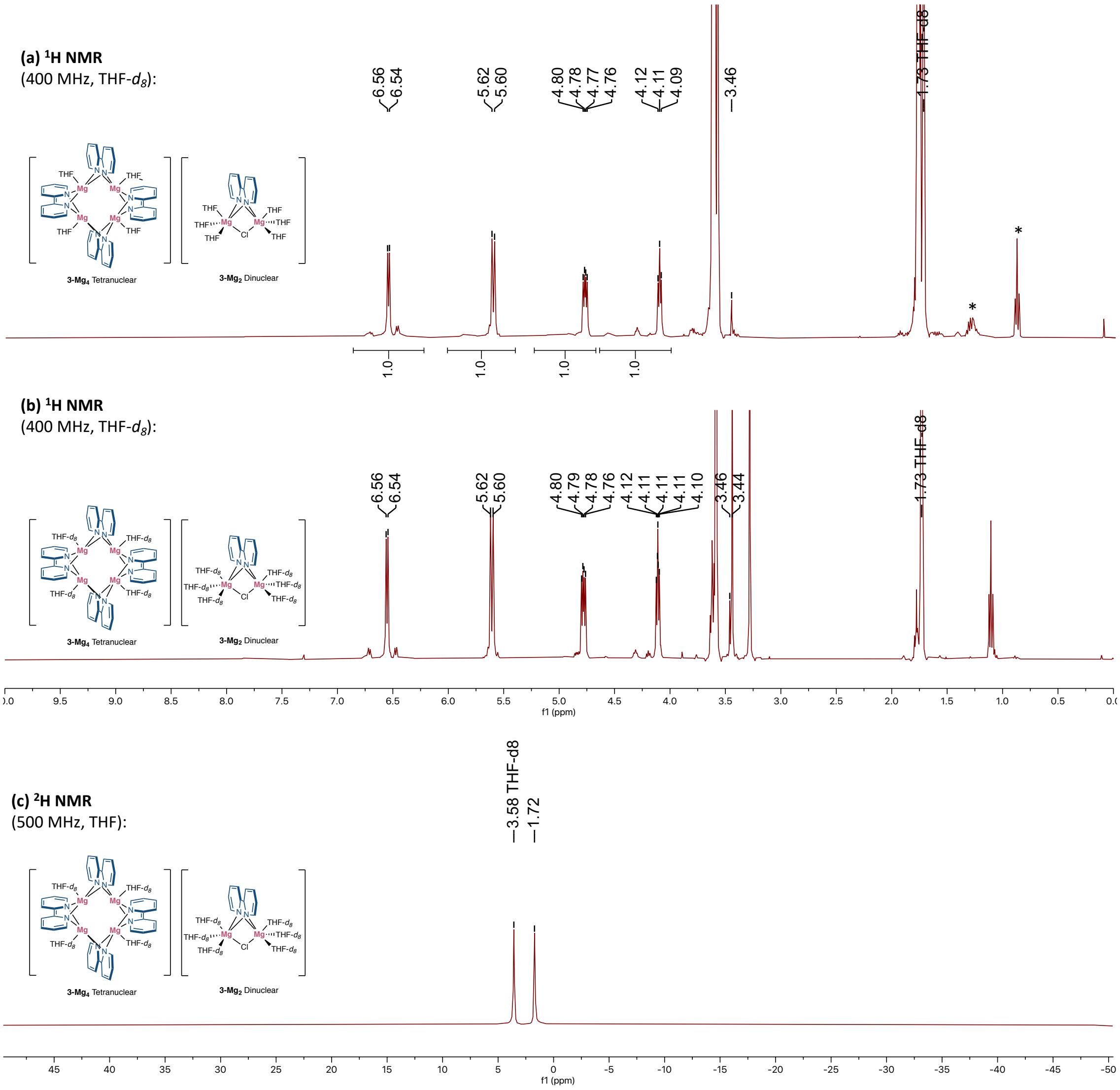

Figure S8. NMR spectra of **3** and **3-THF-*d₈***. (a) reference spectra of **3** synthesized in THF. (b) spectra of **3** synthesized from THF-d_8. (c) ^{2}H NMR spectra of **3** synthesized from THF-d_8. * pentane.

Independent formation of radical anion (bipy)Mg

In the glovebox, bipyridine (20 mg, 0.13 mmol) was added to a 4 mL vial with Mg powder (70 mg, 2.88 mmol). A stir bar was added, and the vial was charged with 2 mL of THF turning the and let stir for 3 hours. After 3 hours the red suspension was filtered through a celite plug and a red solution was collected. The solvent was then removed to afford a red solid and redissolved in 2 ml of THF. The solution was then diluted by half, transferred to an EPR tube, frozen in liq N_2 and analyzed by EPR spectroscopy at -196 °C. The EPR signal observed is consistent with the formation of a bipyridine radical anion.

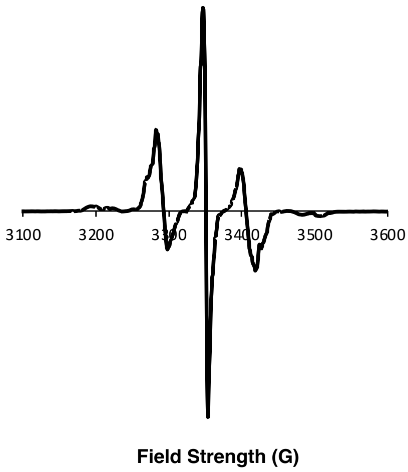

Figure S9. EPR spectra of the bipy-Mg radical anion generated insitu.

S4. IR, UV-VIS, and Cyclic Voltammetry

Infrared Spectroscopy of 3 and 5

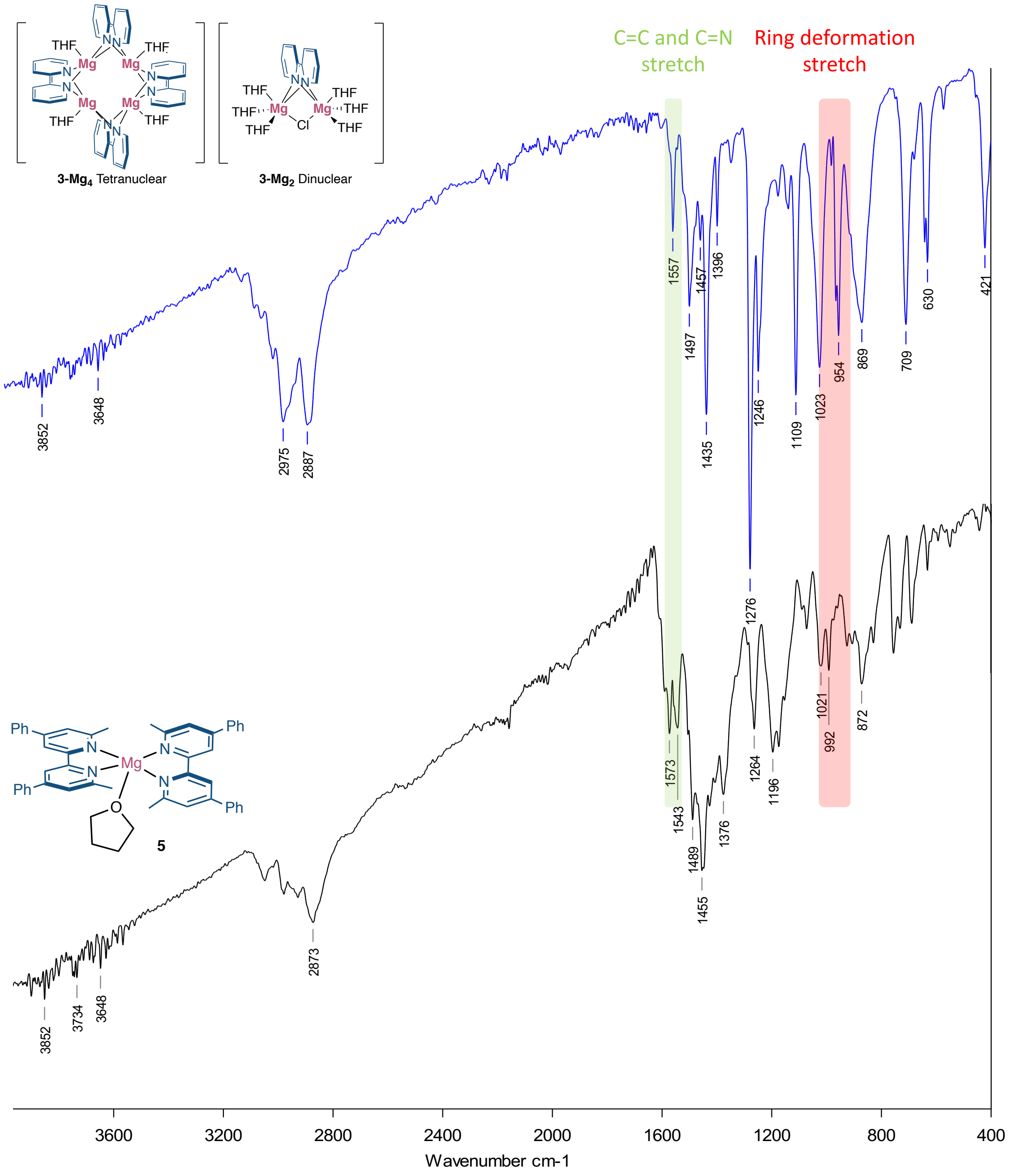

Figure S10. Stack IR spectra of **3** and **5**.

UV-VIS Spectroscopy of 3 and 5

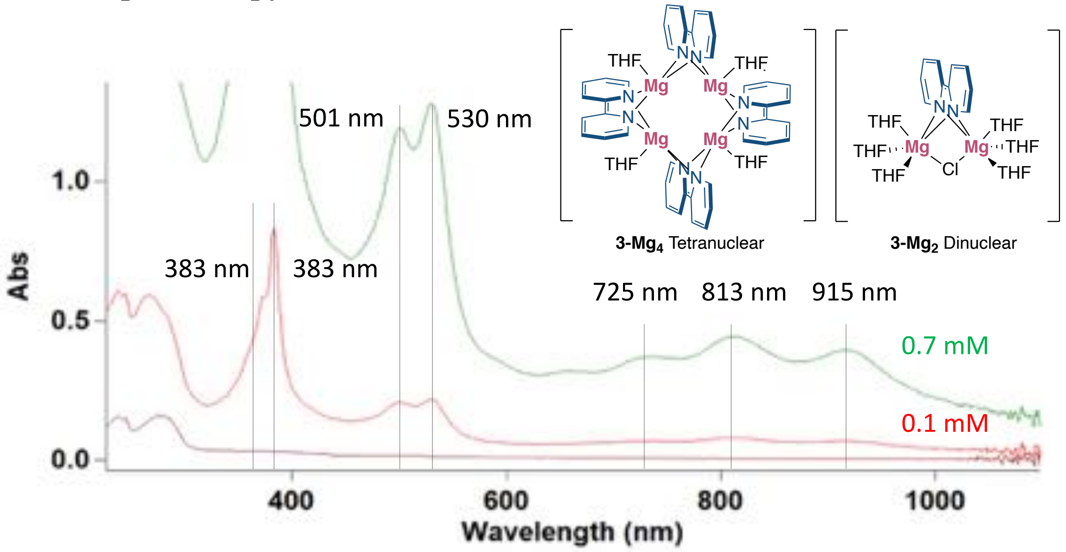

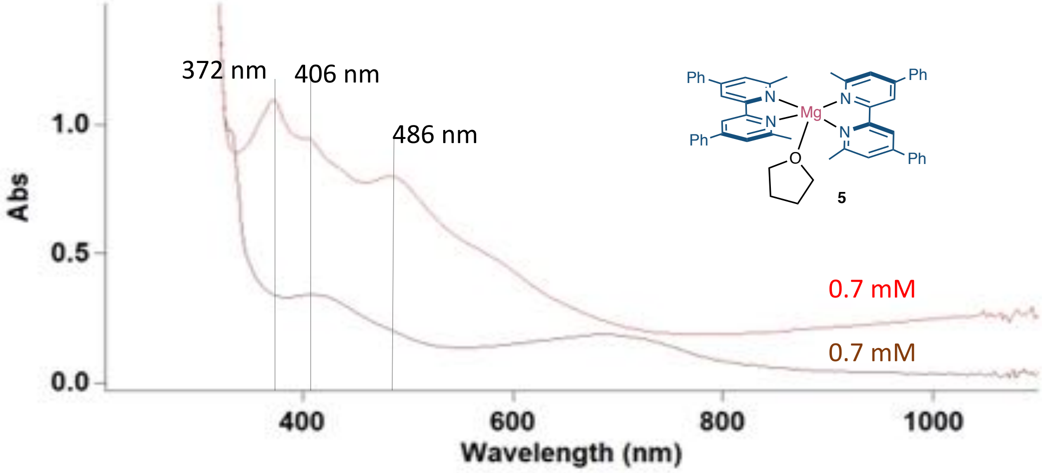

Figure S11. UV-VIS spectra of **3** and **5**. **5** showed instability with the initial scan (red) decomposing to the brown trace.

Cyclic Voltammetry of 1, 3 and 5

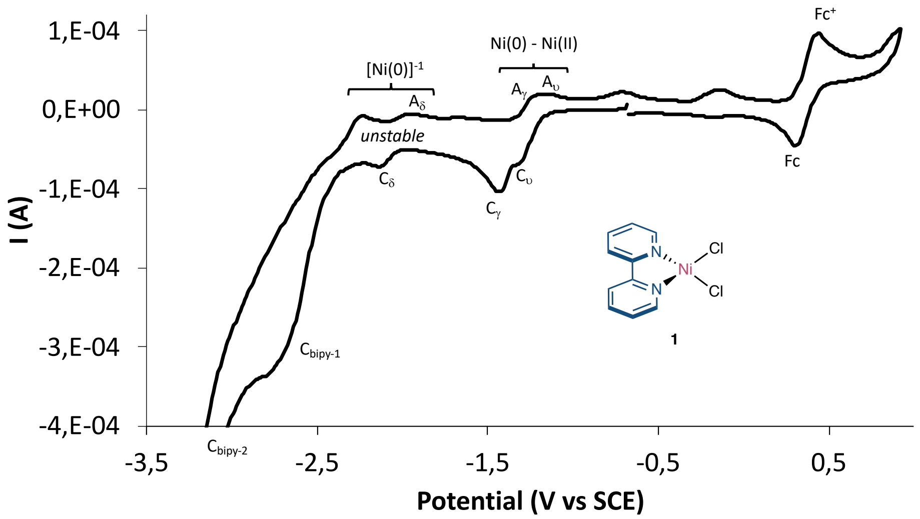

Figure S12. Cyclic voltammogram of (bipy)$NiCl_2$ **1**. Voltammograms were taken using a glassy carbon working electrode in a 0.1 M [$^nBu_4N$][PF_6] supporting electrolyte DMF solution with a 50 mV/s scan rate and 0.01 M of sample referenced to Fc (+0.380 V vs SCE). Scans were started at the open-circuit potential and scanned in the anode direction first; the second cycle is shown here. $E_{1/2}$ values for **1** are Ni^{2+}/Ni^0 = –1.33 V, $[Ni^0]^{1-}$ = –2.09 V and ligand reduction at –2.61 V. **Literature reports** CV of $(bipy)_2Ni$ or $(bipy)_3Ni(ClO_4)_2$ MeCN (0.2 M, TEAP)[5]; Ni^{2+}/Ni^0 = –1.27 V, $[Ni^0]^{1-}$ = –1.97 V, bipyridine radical anion reduction –2.40 V and dianion reduction –2.62 V among other.[1,9]

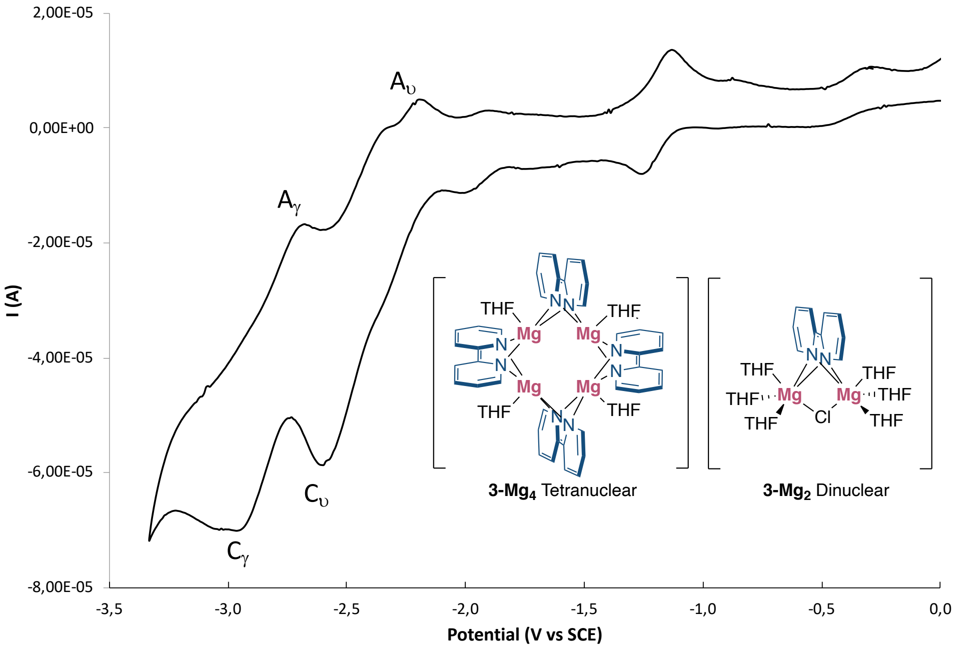

Figure S13. Cyclic voltammogram of **3**. Voltammograms were taken using a glassy carbon working electrode in a 0.1 M [$^nBu_4N$][PF_6] supporting electrolyte THF solution with a 50 mV/s scan rate and 0.01 M of sample referenced to Fc (+0.380 V vs SCE). Scans were started at the open-circuit potential and scanned in the anode direction first; the second cycle is shown here. $E_{1/2}$ values for **3** are Mg^{1-}/Mg^0 = –2.36 V, Mg^{-2}/Mg^{-1} = –2.79 V.

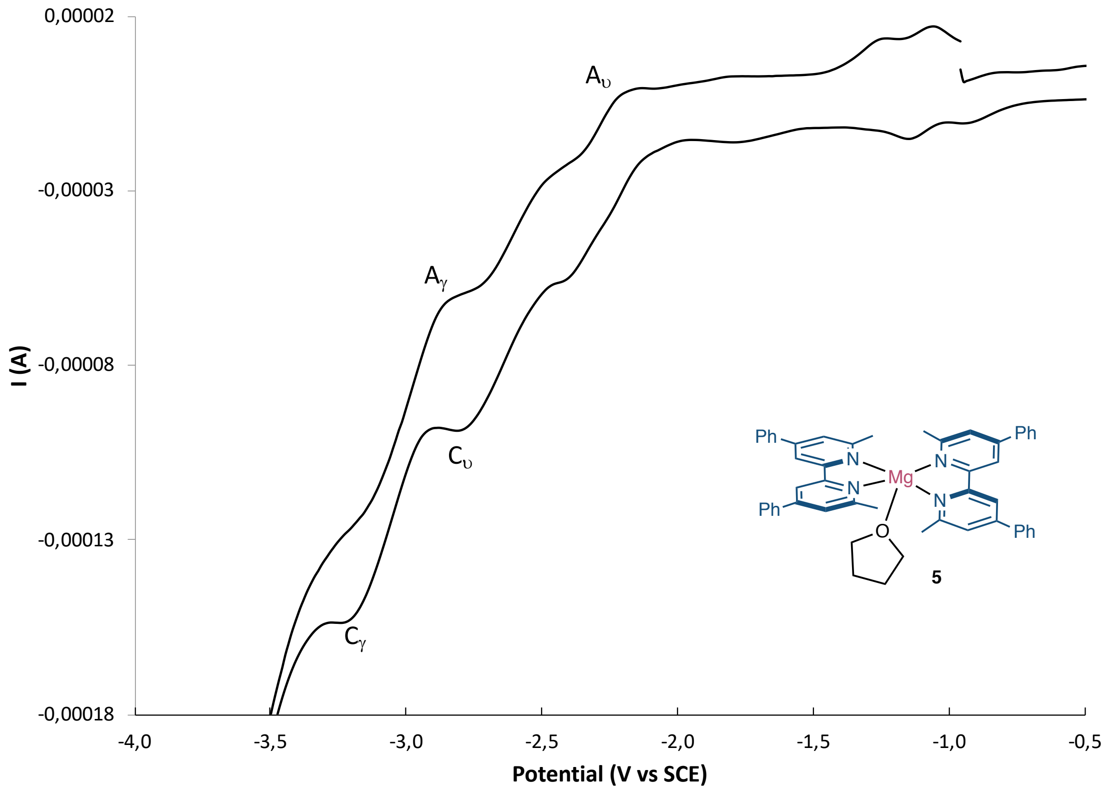

Figure S14. Cyclic voltammogram of **5**. Voltammograms were taken using a glassy carbon working electrode in a 0.1 M [$^{n}Bu_4N$][PF_6] supporting electrolyte THF solution with a 25 mV/s scan rate and 0.01 M of sample referenced to Fc (+0.380 V vs SCE). Scans were started at the open-circuit potential and scanned in the anode direction first; the second cycle is shown here. $E_{1/2}$ values for **5** are Mg^{0}/Mg^{1-} = –2.29 V, $Mg^{1-}/Mg^{1-/1-}$ = –2.60 V $Mg^{1-/1-}$/ Mg^{2-} = –3.04 V.

S5. NMR and EPR Spectra of Synthesized Complexes

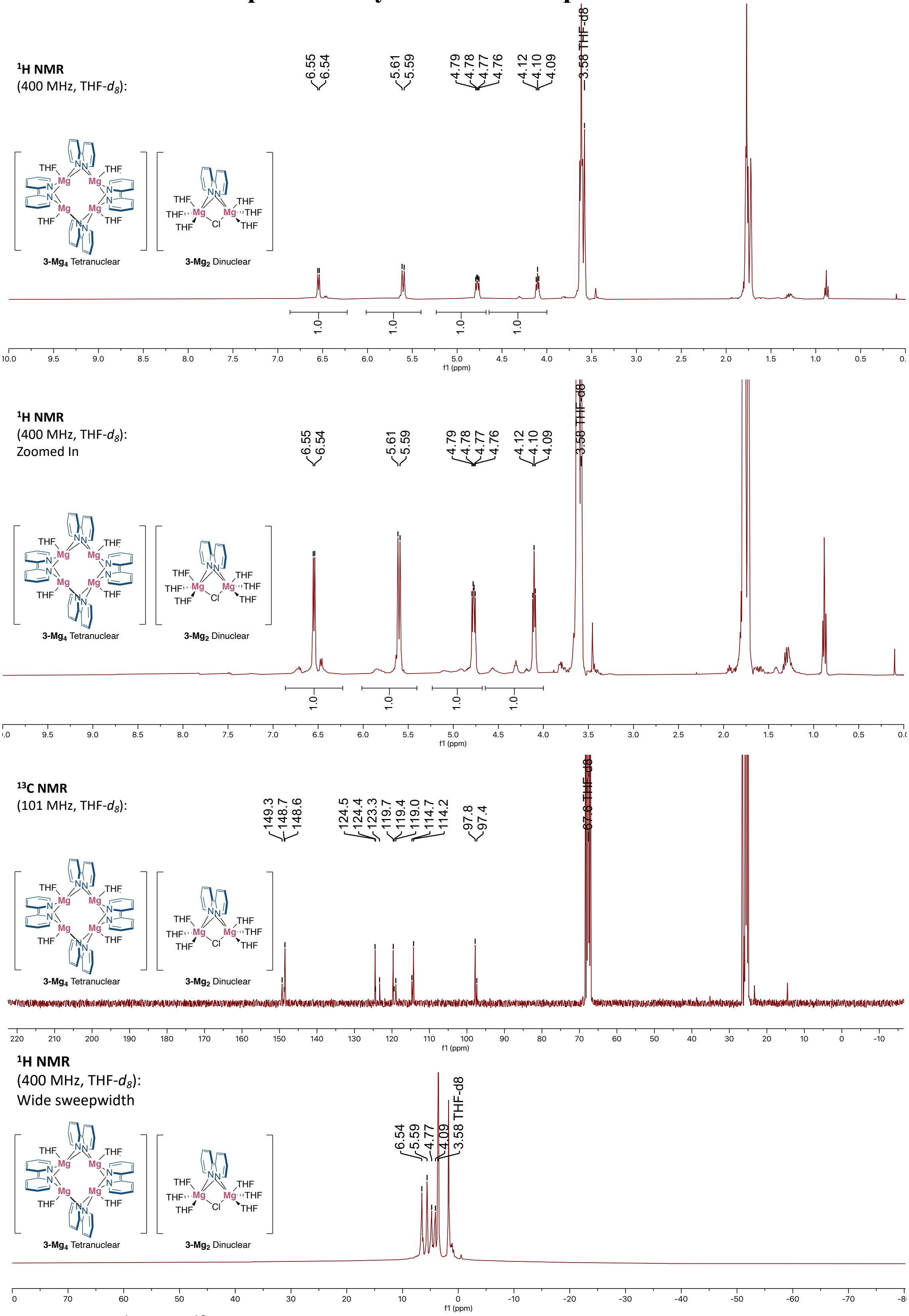

Figure S15. ^{1}H and ^{13}C NMR spectra (THF-d_8, 400/101 MHz) of **3**.

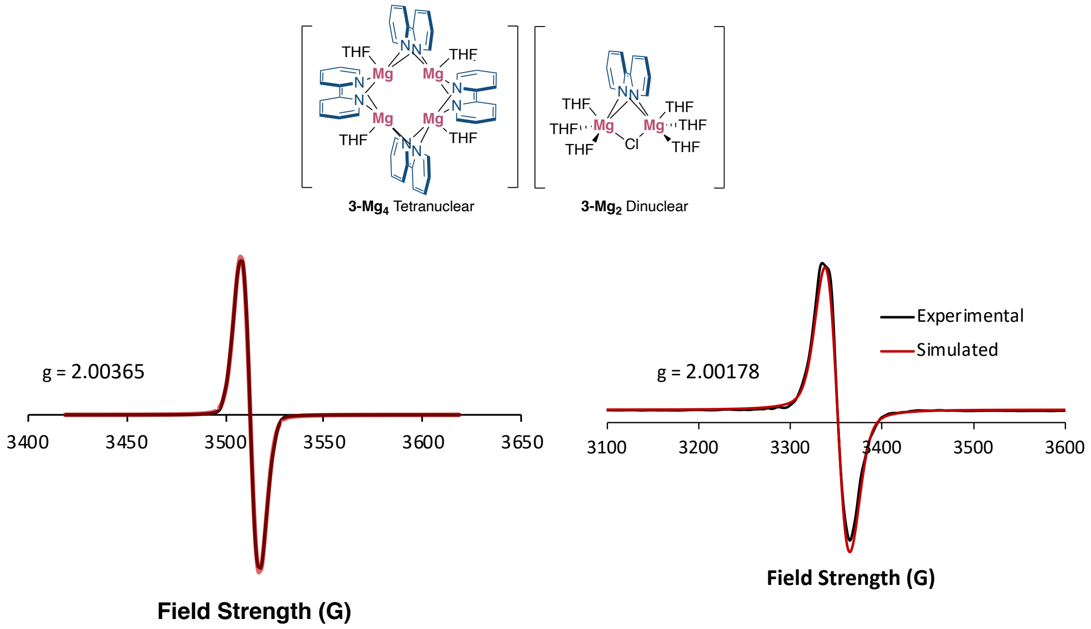

Figure S16. EPR spectrum of **3** (293K, THF, left), g = 2.00365, lineshape = 0.915 and **3** (77 K, THF, right), g = 2.00178, lineshape = 0.767.

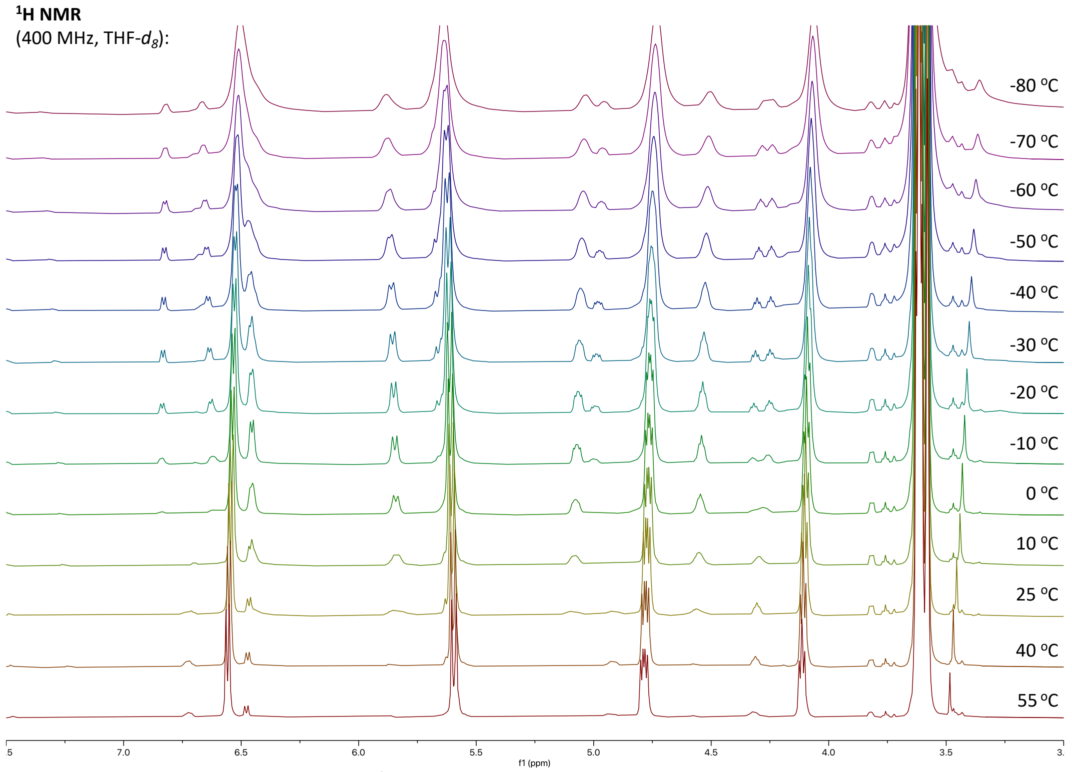

Figure S17. Variable temperature ^{1}H NMR spectra (THF-d_8, 400 MHz) of **3**.

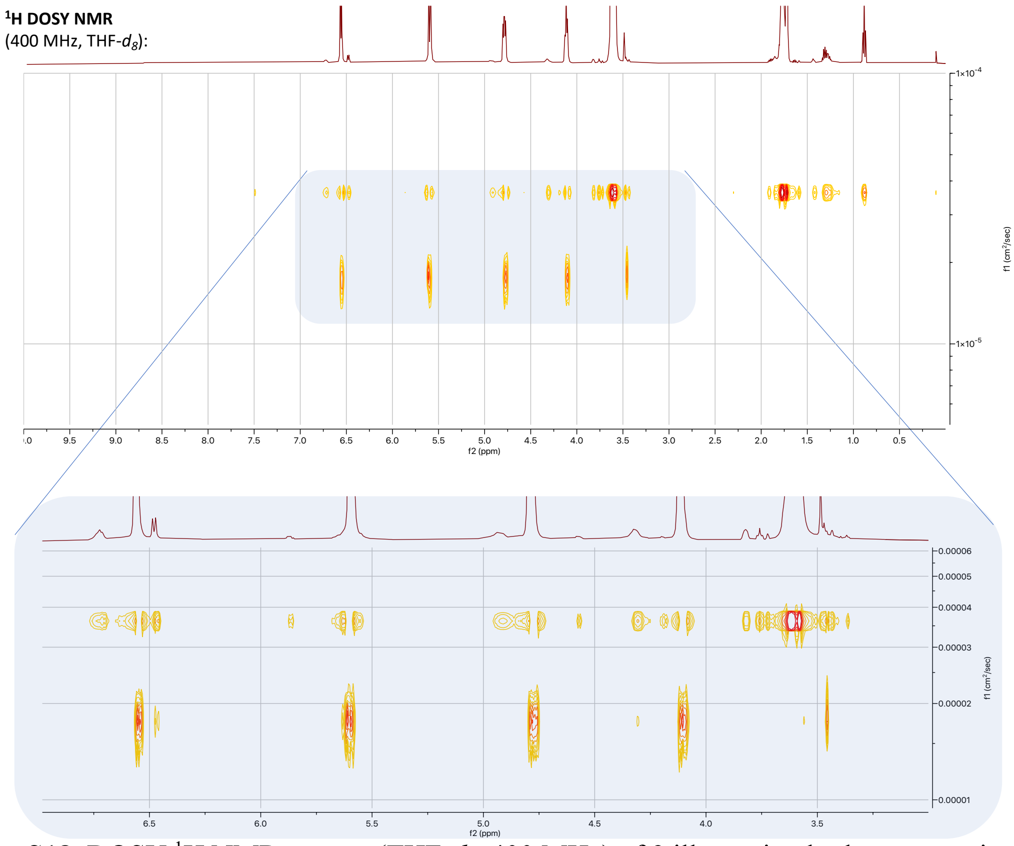

Figure S18. DOSY ^{1}H NMR spectra (THF-d_8, 400 MHz) of **3** illustrating both monomeric and higher order speciation exist in solution.

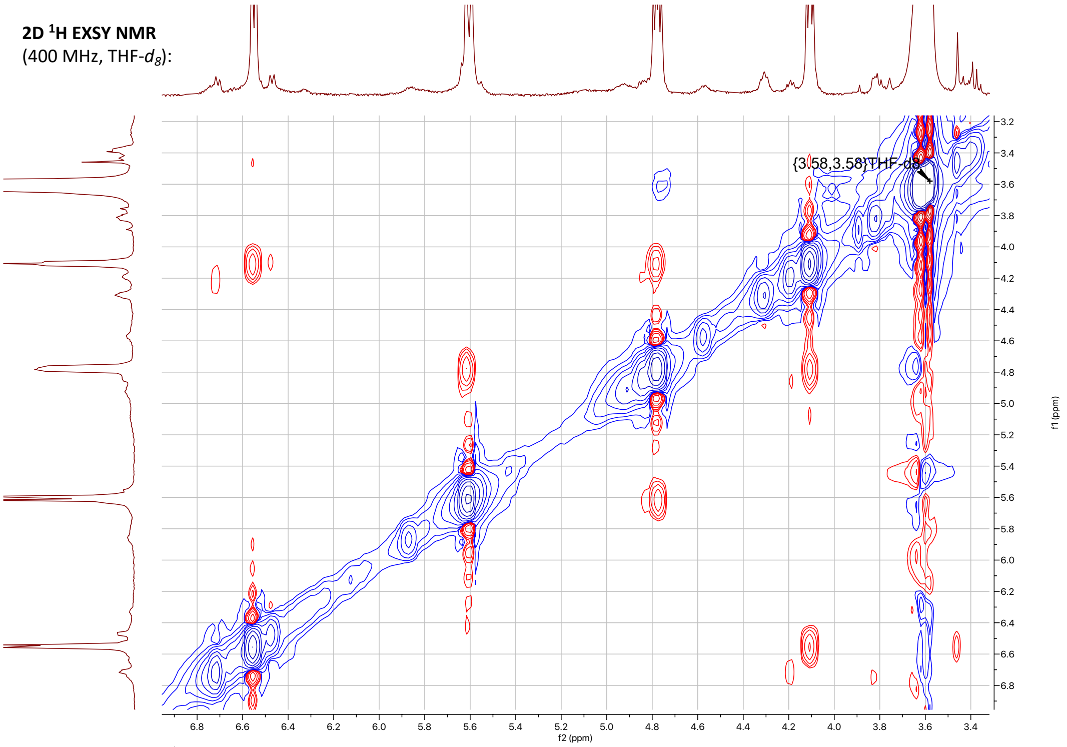

Figure S19. 2D ^{1}H EXSY NMR spectra (THF-d_8, 400 MHz) of **3** demonstrating fluxionality between the bipyridine ligands and THF. Mixing time 0.3 s. Exchange between bipy ligands does not readily occur on the NMR timescale suggesting fluxionality observed during variable temperature analysis originates from fluxional THF ligation or Mg atoms exchange.

EPR quantification of 3

Two solutions of **3** in THF were prepared (1 and 3 mM) and analyzed at 77K, and quantified against a calibration curve of Cu(II) at 77K. **Note:** this approach assumes **3** contains an electron spin ½. The quantification of **3** should be taken as an estimate of the spin concentration as the reference Cu(II) having a different spin system than **3**. This can be seen by the reference, Cu(II), not only has a signal intensity/width that strongly depends on its coordination, but also has a spectral width of ca. 2.5 GHz, while the spectrum of **3** is only ca. 75 MHz wide. In addition, **3** is dissolved in THF, which has very different dielectric losses and hence produces a different EPR signal intensity (depending on resonator and sample geometry). In the measurements, a minor concentration dependence was observed consistent with complex speciation, with slightly over 100 % (105 – 112 %, excluding a nonlinear calibration point) quantification of the unpaired electron of **3** was determined. We attribute this higher quantification to experimental error described in the note above.

Calibration curve[1]		EPR quantification of 3					
c (mM)	Area[2]	Entry	Area[2]	Prepared c (mM)	EPR obtained c (mM)	3 (%)	Comment - Calibration curve:
0,9	17994,4	1	29033,677	1,0	1,3	122	Considering all the points
1,8	52139,8	2			1,2	112	Excluding 3rd point
2,7	57194,4	3	106316,29	3,0	3,5	115	Considering all the points
3,5	119341,9	4			3,2	105	Excluding 3rd point

[1]Calibration curve of $CuSO_4$ in H_2O/Ethylene glycol 1:1. [2]Area calculated in G=2450-3550

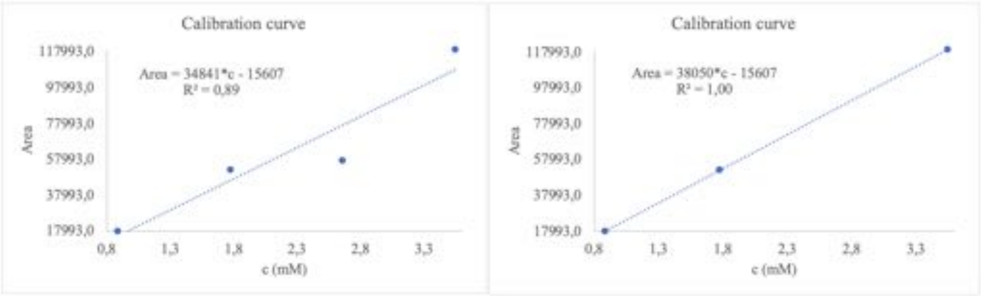

Figure S20. Calibration curve against Cu(II) and EPR quantification of **3** at 77 K.

^{1}H NMR quantification of 3

Quantitative ^{1}H NMR experiments of **3** in THF-d_8 were conducted (1 and 3 mM solutions), using analytically pure [^{n}Bu$_4$][PF$_6$] as calibration standard. In both cases, 100±2 % of **3** was determined. [^{n}Bu$_4$][PF$_6$] was chosen as the internal standard since it is inert and **3** and cannot undergo redox reactions with it.

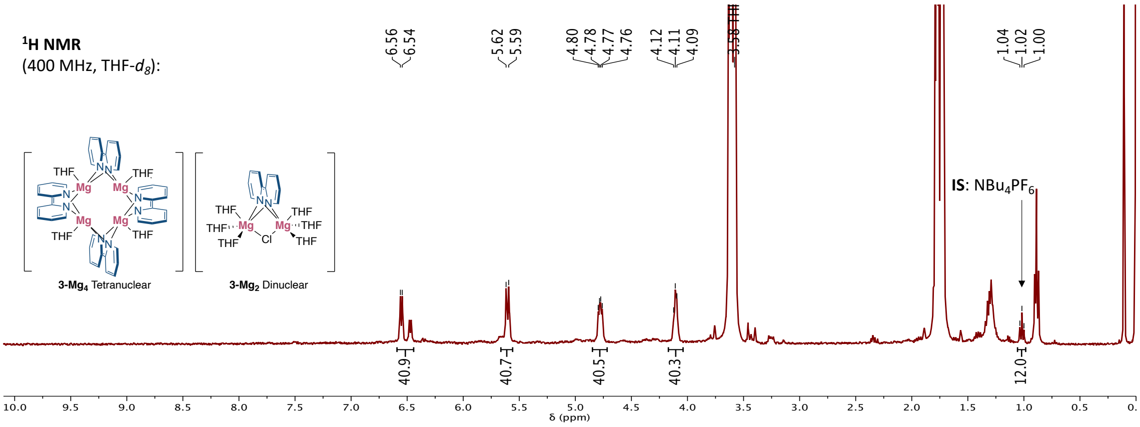

Figure S21. Quantitative ^{1}H NMR spectrum (THF-d_8, 400 MHz) of 3 mM solutions of **3**.

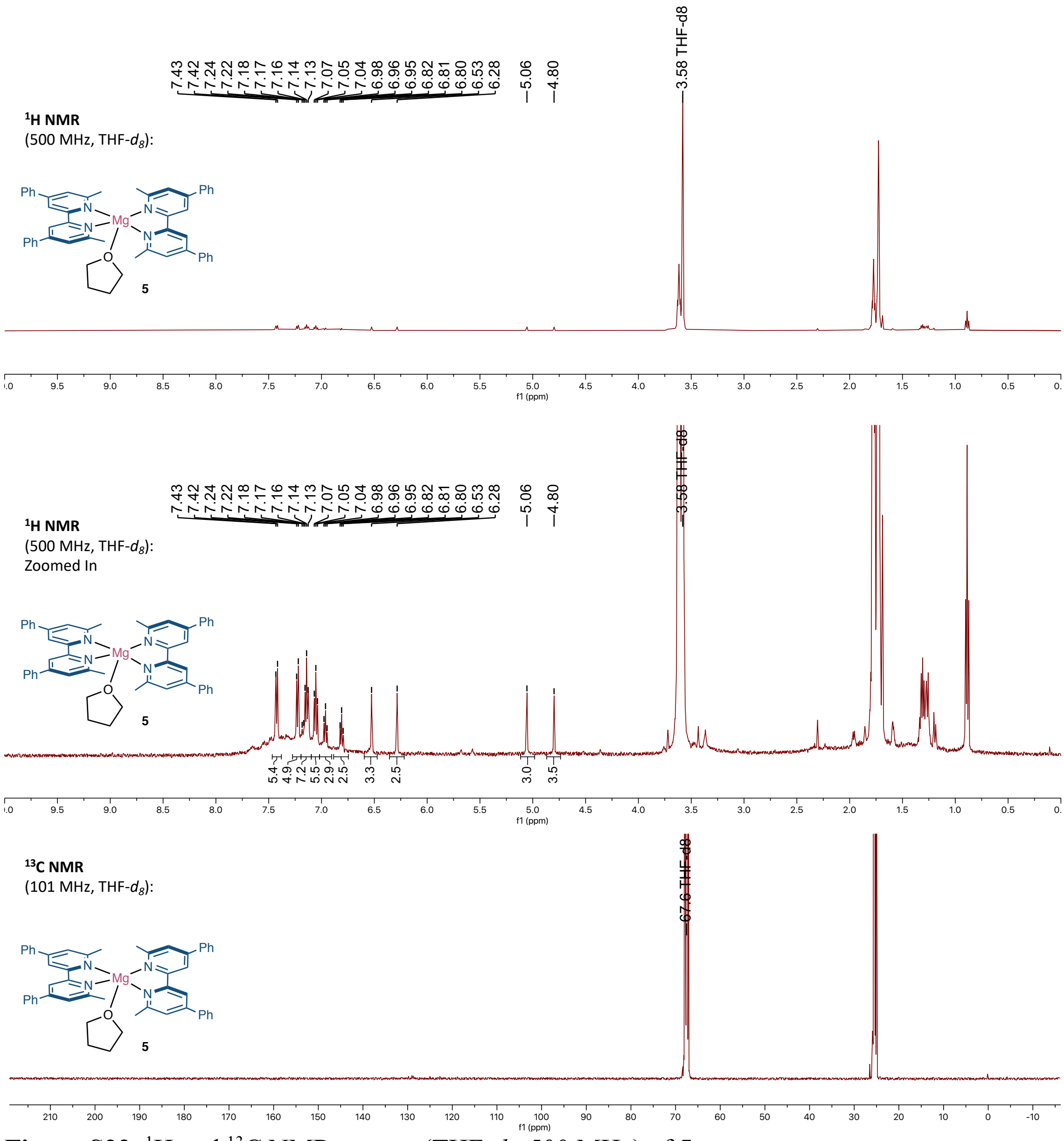

Figure S22. ^{1}H and ^{13}C NMR spectra (THF-d_8, 500 MHz) of **5**.

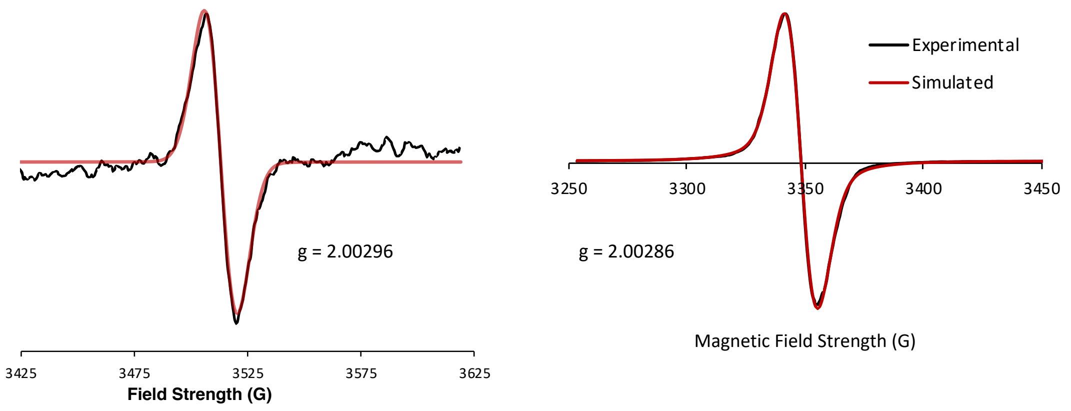

Figure S23. EPR spectrum of **5** (293K, THF), g = 2.00296, lineshape = 0.988 and **5** (77 K, THF, right), g = 2.00286, lineshape = 0.677.

.

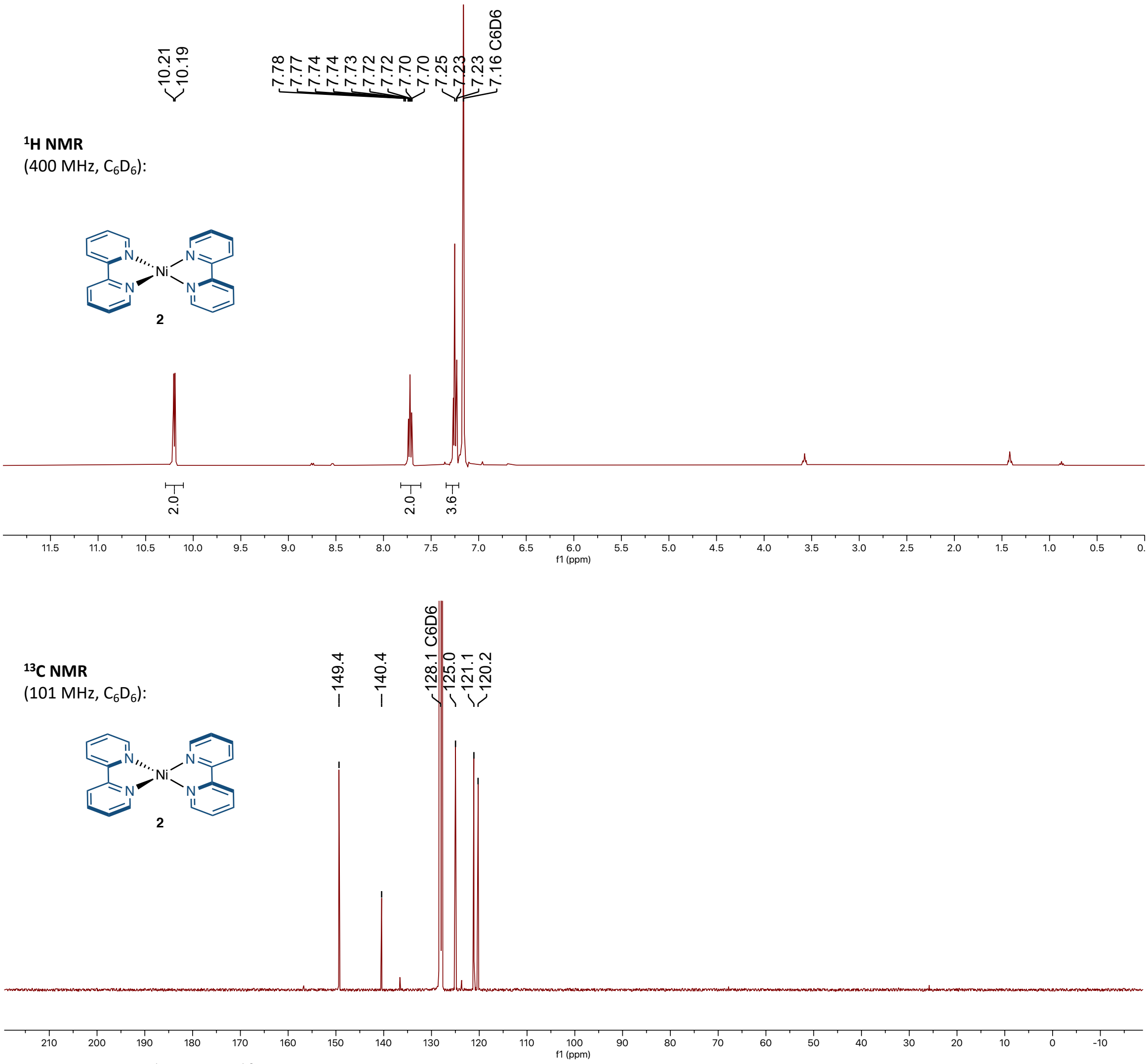

Figure S24. ^{1}H and ^{13}C NMR spectra (C_6D_6, 400 MHz) of $(bipy)_2Ni$ **2**.

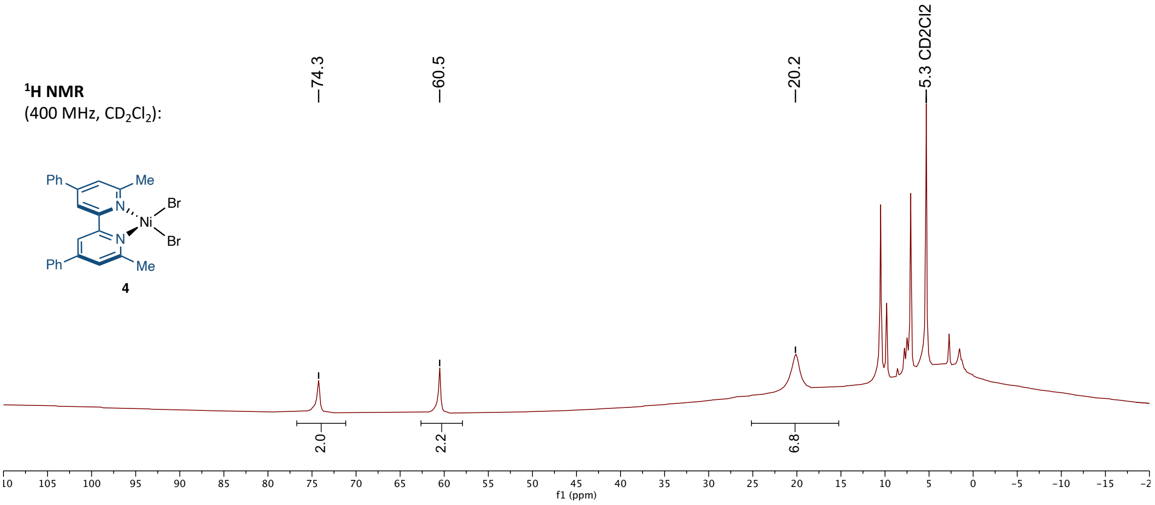

Figure S25. Paramagnetic ^{1}H NMR spectrum (CD_2Cl_2, 400 MHz) of (**L2**)$NiBr_2$ **4**.

S6. Crystallographic Data

Data collection: The measured crystals were prepared under inert conditions immersed in perfluoropolyether as protecting oil for manipulation.

Crystal structure determination for compound **(bipy)$_2$Mg(THF)$_2$** was carried out using a Rigaku diffractometer equipped with a Pilatus 200K area detector, a Rigaku MicroMax-007HF microfocus rotating anode with MoK_α radiation, Confocal Max Flux optics and an Oxford Cryosystems low temperature device Cryostream 700 plus (T = -173 °C). Full-sphere data collection was used with ω and φ scans. *Programs used:* Data collection data reduction with CrysAlisPro[14] and absorption correction with Scale3 Abspack scaling algorithm.[15]

Crystal structure determination for **3** (crystal type C) was carried out using an Apex DUO Kappa 4-axis goniometer equipped with an APPEX 2 4K CCD area detector, a Microfocus Source E025 IuS using CuK_α radiation, Quazar MX multilayer Optics as monochromator and an Oxford Cryosystems low temperature device Cryostream 700 plus (T = -173 °C). Full-sphere data collection was used with ω and φ scans. *Programs used:* Data collection APEX II,[15] data reduction Bruker Saint[16] V/.60A and absorption correction TWINABS.[17]

Crystal structure determination for **2**, **5**, **6** and **L2** was carried out using an Apex DUO Kappa 4-axis goniometer equipped with an APEX II 4K CCD area detector, a Microfocus Source E025 IuS using MoK_α radiation, Quazar MX multilayer Optics as monochromator and an Oxford Cryosystems low temperature device Cryostream 700 plus (T = -173 °C). Full-sphere data collection was used with ω and φ scans. *Programs used:* Data collection APEX II,[16] data reduction Bruker Saint[17] V/.60A and absorption correction SADABS.[18]

Structure Solution and Refinement: Crystal structure solution was achieved using the computer program SHELXT.[19] Visualization was performed with the program SHELXle.[20] Missing atoms were subsequently located from difference Fourier synthesis and added to the atom list. Least-squares refinement on F^2 using all measured intensities was carried out using the program SHELXL 2015.[21] All non-hydrogen atoms were refined including anisotropic displacement parameters.

Comments for the structures:

Compound 3: Due to the difficulties in obtaining high quality single crystals for **3** and in order to confirm unambiguously this structure and specially the oxidation status of the metal atoms, several crystallizations and measurements were performed. While as a powder, compound **3** is stable in a nitrogen glovebox, in the crystalline form is extremely sensitive to solvent loss (THF) and readily oxidizes under aerobic atmosphere (crystallizations were performed in the glovebox freezer), various methods of crystal preparation were performed (e.g chilling with dry CO_2, a N_2 stream at the microscope, etc.). Additionally, the preparation was further complicated as the colour of the crystals are very dark and opaque with a very dark mother liquor. The general tendence of this compound to also crystallize as multicomponent crystals or twins make this a very challenging compound to work with. Finally, 16 crystals were measured. Different cells in one crystal corresponded to different indexations of the same dataset (27 indexed datasets) containing the **3-Mg$_4$/3-Mg$_2$** couple. A summary of the dataset collected is shown in Table S1.

Table S1. Table of 16 different measured crystals, corresponding to each crystal code. Different cells correspond to different indexations of the same dataset (27 indexations). The dark grey marked line corresponds to the best dataset measured.

Crystal	Crystal code	Cell	Space group	a	b	c	α	β	γ	R1 (%)	Ratio reflect /par	Comments
A	mo_CSD0 533_frag	monoclinic	*P*2/*c*	40.00	22.60	23.72	90.00	95.70	90.00	14.63	6.27	Initial structure obtained; highly disordered
		monoclinic	*C*2/*c*	23.70	22.60	37.40	90.00	90.06	90.00	25.10		TWIN 1 0 0 0 -1 0 0 0 -1
		monoclinic	*C*2/*c*	44.20	22.60	23.70	90.00	122.41	90.00	26.60		TWIN 1 0 2 0 1 0 0 0 -1
		orthorhombic	*P*bca	22.65	37.46	23.75	90.00	90.00	90.00	30.60		
		orthorhombic	*P*bcn	37.46	22.65	23.75	90.00	90.00	90.00	29.23		
		monoclinic	*C*2/m	23.75	22.64	37.36	90.00	90.06	90.00	24.92		
		orthorhombic	*I*bam	37.54	22.66	23.80	90.00	90.00	90.00	37.60		
A	58d-21 COJ311	orthorhombic	Pbcn	23,58	37,07	22,45	90,00	90,00	90,00	14,28	2,00	Extremly small crystal, not enough data
		orthorhombic	Pca2(1)	23,58	37,06	22,45	90,00	90,00	90,00	15,55	2,00	
D	58e-21 COJ311	triclinic	P1	17,93	18,43	18,98	97,83	92,07	118,07	14,76	4,80	Extremly disordered
D		triclinic	P-1	17,93	18,43	18,98	97,83	92,07	118,07	17,04	4,47	
D	58f-21 COJ311	triclinic	P-1	17,94	18,35	18,92	98,04	92,42	117,93			bad crystal
D	58ff-21 COJ311	triclinic	P-1	17,92	18,28	18,92	98,18	92,12	117,80			bad crystal
D	58ffff-21 COJ311	triclinic	P1	17,92	18,28	18,92	98,18	92,12	117,80	15,35	6,20	
D		triclinic	*P*-1	17.92	18.28	18.92	98.18	92.12	117.80	15.64	6.20	
D	247a-21 CSD08Mg Cluster	triclinic	P-1	18,00	18,41	18,96	98,33	92,13	117,73	20,00	3,50	
D	247b-21 CSD08Mg Cluster	triclinic	P-1	17,97	18,41	18,95	98,15	92,08	117,81	14,29	9,00	
C	58g-21 COJ311	monoclinic	C2/c	44,88	22,58	23,72	90,00	110,28	90,00	10,58	10,78	Multicomponent crystal with two domains
	58gg-21 COJ311	monoclinic	P21/c	42,99	22,64	23,81	90,00	101,05	90,00	24,08	9,69	
	58h-21 COJ311	triclinic	P1 / P2/m	22,64	23,81	43,06	79,31	90,04	89,97			bad crystal
C	58hh-21 COJ311	monoclinic	C2/c	45,00	22,67	23,76	90,00	110,26	90,00	24,45	3,08	twin5
C	58j-21 COJ311	monoclinic	C2/c	44,97	22,69	23,76	90,00	110,26	90,00	22,41	27,41	twin? Comprovar amb g
B	58i-21 COJ311	monoclinic	P2/c	39,92	22,61	23,70	90,00	90,80	90,00	19,66		TWIN 1 0 0 0 -1 0 0 0 -1
		monoclinic	P21/c	42,38	22,77	23.97	90,00	90,40	90,00	30,00		TWIN 1 0 0 0 -1 0 0 0 -1
B		orthorhombic	*P*bcn	42,34	22,71	23,88	90,00	90,00	90,00	13,38	5,51	
	58ii-21 COJ311	monoclinic	P2/c	39,98	22,64	23,76	90,00	95,87	90,00	24,00	3,70	
C	279a-21 CSD08Mg cluster	monoclinic	C2/c	45,10	22,64	23,80	90,00	110,20	90,00	7,15	13,78	TWIN 5 BASF 0.38

In general, it was observed that the crystal form measured depends on the crystallization conditions and on the speed by mounting the crystals. Generally, crystallizations of one same batch contained the same crystalline phase. Crystals mounted in a time below 5 seconds and directly form the suspension in the mother liquor under nitrogen, gave the best diffracting datasets but were difficult to clean up in relation of different crystalline domains. Crystals prepared first in the microscope under oil coating, presented different crystalline phases than the previous ones and showed a much higher mosaicity. In contrast, these were easier to select and clean up. In general, the loss of THF in the crystal lead to changes in the crystal phase until it decomposed.

In the series of performed measurements a total of four different crystal structures (A, B, C and D) could be solved and refined which contained always the same organometallic core (**3-Mg_4/3-**

Mg_2 couple, see below); with a tremendous effort to obtain the best description of this new structure. For the description of this new compound in this paper the best dataset refined was selected (Structure type C). Structures with different unit cells displayed varied amounts of residual electron density which ranged from 0.6 - 3.2 e/$Å^3$ in the center of Mg_4, supporting that this density should be considered as a qualitative guide. The best dataset displayed a residual density of 1.9 e/$Å^3$. We further note that parameters such as modifying the integration areas of peaks, or changing the settings of absorber strength can change this electron density by around 1 e/$Å^3$ which supports the qualitative nature of these measurements. The complete description of all different structures obtained for this compound will be published elsewhere in a specialized journal.

Compound 3, crystal C:

A black crystal of compound **3** (crystal type C) was mounted directly extracted from the N_2-protected THF suspension without manipulation into the diffractometer at 100 K. The crystal was coated in perfluoropolyether as protecting oil and mounted in a time below 5 seconds. It resulted to be a multicomponent crystal from which two crystals could be indexed successfully. To reduce reflection overlapping the measurement was performed using $Cu_{K\alpha}$-radiation and the sample was processed with TWINABS[17] used for scaling and absorption correction of multicomponent crystals. The ratio of the two crystals after refinement (BASF value) achieved a value of 62:38. The structure obtained was of good quality (R1-value of 7.15 %) and for both crystals refined in the monoclinic space group *C*2/c an observed reflections to parameter ratio of 13,78 was reached. The asymmetric unit contains one salt unit formed by a square Mg_4-cluster anion (3-Mg_4) and Mg_2Cl_1 cationic metal-complex molecule (3-Mg_2), and 6 ½ non-coordinated THF molecules. The 3-Mg_4 anion corresponds to a square Mg cluster coordinated to four bipyridine molecules and four THF molecules. The 3-Mg_2 cation corresponds to a bimetallic Mg complex with a chloro-bridge which is coordinated to one bipyridine molecule and six THF molecules. The 6.5 non-coordinated THF molecules are disordered in 11 different locations. In the organometallic core most of the coordinated THF molecules are disordered in two orientations. Noteworthy a large residual electron density was located in the center of the four membered Mg-cluster which corresponds to a concentration of negative charge in the anionic part of the Mg-salt formed (1.9 e$Å^{-3}$). We suspect the greater than 1 e$Å^{-3}$ observed is due to the inherent challenges of quantitatively measuring electron density and the Mg atoms adjacent to the electride which may contribute to the observed electron density. This electron density is localized reproducibly in structures refined for crystal types A, B, C and D and is consistent with performed calculations.

Compound 2: The measured sample was formed by several crystals from which three could be indexed. Two minor crystals were ignored and only the larger crystal was used for integration. All samples tested were formed by several crystals and no better data could be collected. Although the structure is already known, the measured crystal corresponds to an unpublished polymorph of this compound. This structure unambiguously confirmed our assignment of **2** in its synthesis, which was lacking ^{1}H and ^{13}C NMR spectra in the literature. The refined crystal crystallized in the monoclinic space group *C*2/c as a real twin and the matrix -1 0 0 0 -1 0 1 0 1 (the minor twin component contribution was of 9 %; basf: 0.09) was applied. The asymmetric unit contains two half independent molecules of the metal complex which have *C*2 symmetry. Unusual residual densities and low bond precision were attributed to the presence of additional crystals in the

sample measured and effects of twinning. The structure was considered of enough quality to be published.

Compound 5: Crystals measured for this sample were black and extremely difficult to select from the suspension. Mounted crystals were always formed by several single crystals (multi-component crystal or twin). The selected sample was also formed from several crystals in which five single crystals could be indexed (Crystals AA, BB, CC, DD and EE). Three different cells were identified. Crystal AA: a: 11.88 Å, b: 7.5 Å, c: 20.20 Å, α: 90º, β: 90º γ: 90º; Orthorhombic P. Crystal BB: a: 13.12 Å, b: 13.38 Å, c: 14.67 Å, α: 67.1º, β: 85.9º γ: 69.4º; Triclinic P. Crystal CC: a: 13.1 Å, b: 13.5 Å, c: 14.5 Å, α: 67.0º, β: 85.7º γ: 69.1º; Triclinic P. Crystal DD: a: 11.05 Å, b: 7.36 Å, c: 11.84 Å, α: 90º, β: 117.3º γ: 90º; Monoclinic P. Crystal EE: a: 11.07 Å, b: 7.38 Å, c: 11.86 Å, α: 90º, β: 117.0º γ: 90º; Monoclinic P. Crystals AA and EE were integrated, solved and refined. Both (AA and EE) corresponded to the structure of the ligand in two polymorphic forms (This ligand was used for forming the metal-complex structure; ligand: $C_{24}H_{22}N_2$). The structure corresponding to crystal EE was not added to this paper. Crystal AA is described as Compound L2. Crystal BB was also integrated, solved and refined and corresponded to the expected structure. The asymmetric unit of crystal BB contains one molecule of the Mg metal complex and one non-coordinated THF molecule. Low bond precision on C-C bonds (0.00429) and deviations for the K values in the analysis of variance (C-alerts) were considered an effect of the presence of several crystals in the sample. Despite of the presence of the other crystals in the sample, the structure of crystal BB was of good quality and considered suitable for publication. The integration was performed using only the orientation matrix for crystal BB ignoring possible overlapping reflections of the other crystals present.

Compound L2: Compound L2 corresponds to crystal AA to the previous described multi-component crystal sample. The asymmetric unit contains half a molecule of the ligand which show *C*i-symmetry.

Compound 6: The two THF molecules attached to the Magnesium atom are disordered in two orientations (approximate ratio: 50:50).

Compound (Bipy)$_2$Mg(THF)$_2$: This compound crystallizes in the tetragonal space group P-42$_1$c. The asymmetric unit contains half molecule of the Mg metal complex.

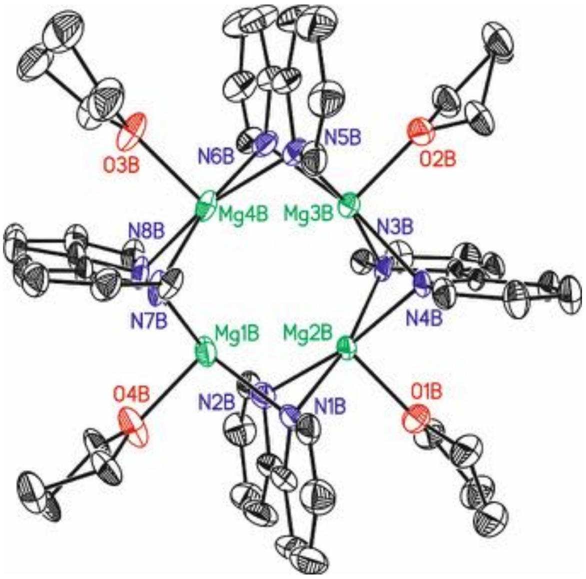

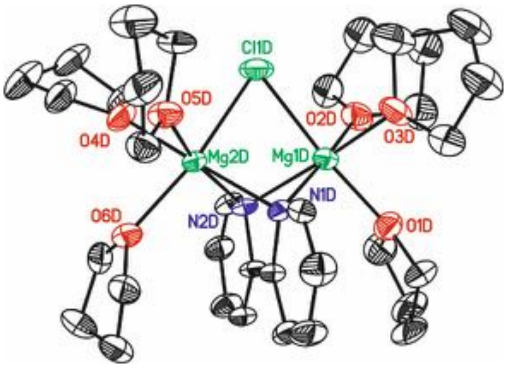

Figure S26. ORTEP drawing (50 %) showing one of the tetranuclear (**3-Mg$_4$**) and one of the binuclear groups (**3-Mg$_2$**) present in the structure of [(THF)$_4$Mg$_4$(μ^2-bipy)$_4$][(THF)$_6$Mg$_2$(μ^2-bipy)(Cl)] **3**. Non-coordinated solvent molecules (THF), disordered parts and hydrogen atoms have been omitted in the sake of clarity. CCDC deposition numbers **CCDC-2151121**.

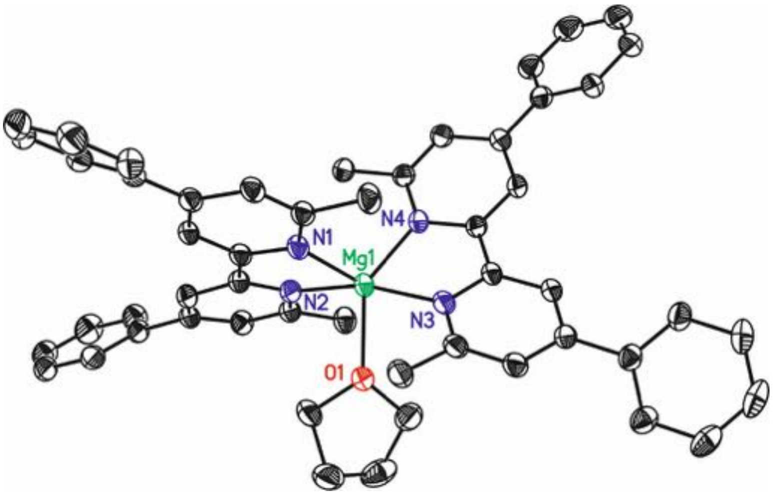

Figure S27. ORTEP drawing (50 %) showing Mg(4,4-phenyl-2,2-methyl-bipyridine)$_2$(THF) **5**. A non-coordinated THF molecule and hydrogen atoms have been omitted in the sake of clarity. CCDC deposition number **CCDC-2060212.**

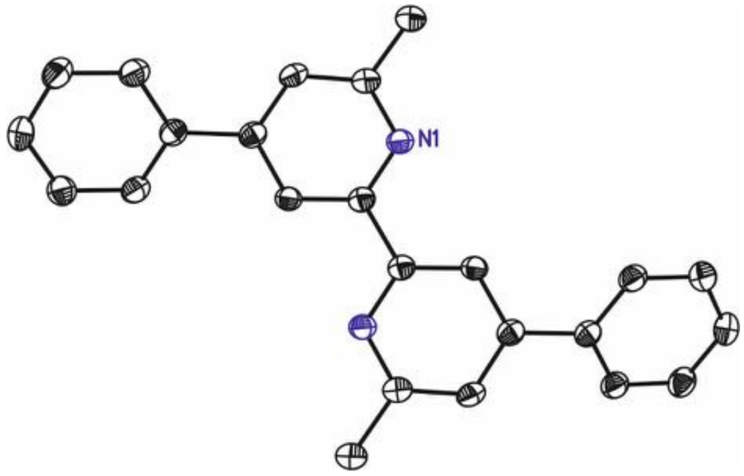

Figure S28. ORTEP drawing (50 %) showing 4,4-phenyl-2,2-methyl-bipyridine **L2**. Hydrogen atoms have been omitted in the sake of clarity. CCDC deposition number **CCDC-2060213.**

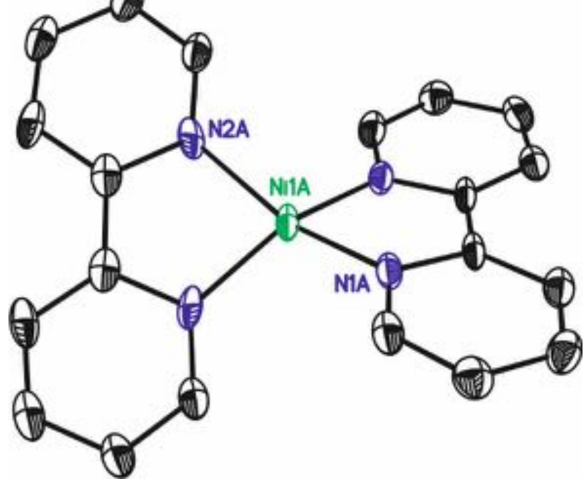

Figure S29. ORTEP drawing (50 %) showing one of the independent molecules contained in the structure of (bipy)$_2$Ni **2**. Hydrogen atoms have been omitted in the sake of clarity. CCDC deposition number **CCDC-2060211.**

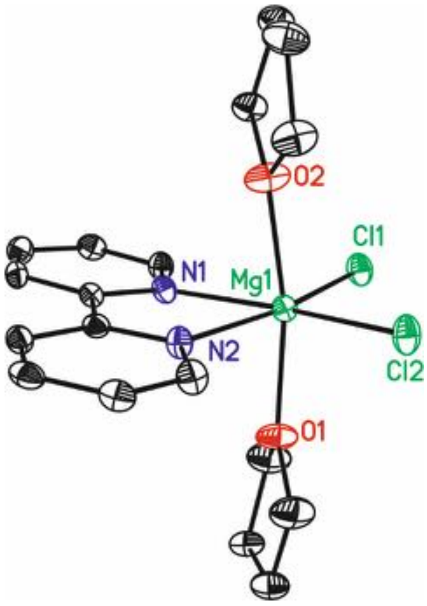

Figure S30. ORTEP drawing (50 %) showing (bipy)MgCl$_2$(THF)$_2$ **6**. Hydrogen atoms and disordered parts have been omitted in the sake of clarity. CCDC deposition number **CCDC-2060214.**

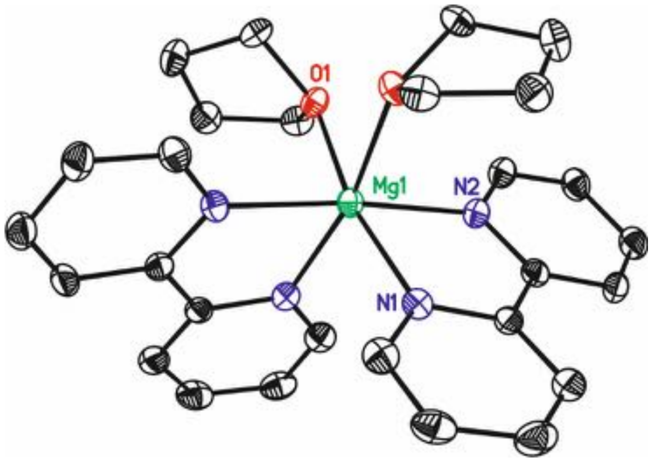

Figure S31. ORTEP drawing (50 %) showing (bipy)$_2$Mg(THF)$_2$. Hydrogen atoms have been omitted in the sake of clarity. CCDC deposition number **CCDC-2060210.**

Table S2. Crystallographic Data

	**3 crystal type C**	**6**	**(bipy)$_2$Mg(THF)$_2$**
**Formula**	$C_{115}H_{170}Cl_1Mg_6N_{10}O_{16.25}$	$C_{18}H_{24}Cl_2MgN_2O_2$	$C_{56}H_{64}Mg_2N_8O_4$
**Formula weight**	2133.91	395.60	961.77
**T (K)**	100(2)	100(2)	100(2)
**Wavelength (Å)**	1.54178	0.71073	0.71073
**Crystal system**	Monoclinic	Monoclinic	Tetragonal
**Space group**	*C*2/c	$P2_1$/n	*P*-4 2_1c
a **(Å)**	45.107(5)	9.1946(9)	12.8507(4)
b **(Å)**	22.640(2)	15.3698(13)	12.8507(4)
c **(Å)**	23.805(3)	13.4637(12)	14.7557(10)
**α (deg)**	90	90	90
**β (deg)**	110.211(3)	97.066(3)	90
**γ (deg)**	90	90	90
**V (Å^3)**	22813(4)	1888.2(3)	2436.8(2)
**Z**	8	4	2
**Density (calc.) (Mg/m^3)**	1.243	1.392	1.311
**μ (mm^{-1})**	1.158	0.391	0.107
**F(000)**	9192	832	1024
**Crystal size (mm^3)**	0.50 x 0.20 x 0.20	0.080 x 0.040 x 0.010	0.200 x 0.020 x 0.020
**Theta range for data collection (deg)**	2.087 to 68.154	2.020 to 27.521	2.761 to 29.273
**Index ranges**	$-54 \le h \le 50$, $0 \le k \le 26$, $0 \le l \le 28$	$-11 \le h \le 11$, $-19 \le k \le 11$, $-17 \le l \le 17$	$-11 \le h \le 17$, $-17 \le k \le 10$, $-19 \le l \le 20$
**Reflections collected**	93425	16728	7037
**Independent reflections**	30367[R(int) = 0.0566]	4278[R(int) = 0.0889]	2741[R(int) = 0.0482]
**Completeness to theta**	96.1 % 68.154°	98.7 % 27.521°	93.0% 29.273°
**Absorption correction**	Multi-scan	Multi-scan	Multi-scan
**Max. and min. transmission**	0.75 and 0.51	0.74 and 0.67	1.00 and 0.59
**Refinement method**	Full-matrix least-squares on F^2	Full-matrix least-squares on F^2	Full-matrix least-squares on F^2
**Data / restraints / parameters**	30367/ 3441/ 1980	4278/ 318/ 480	2741/ 0/ 159
**Goodness-of-fit on F^2**	1.039	1.013	1.012
**Final R indices [I>2sigma(I)]**	R1 = 0.0715, wR2 = 0.1988	R1 = 0.0519, wR2 = 0.0914	R1 = 0.0429, wR2 = 0.0749
**R indices (all data)**	R1 = 0.0791, wR2 = 0.2080	R1 = 0.1175, wR2 = 0.1131	R1 = 0.0738, wR2 = 0.0831
**Largest diff. peak and hole**	1.897 and -0.642 e.Å^{-3}	0.420 and -0.342 e.Å^{-3}	0.210 and -0.220 e.Å^{-3}

Table S3. Crystallographic Data

	**5**	**L2**	**2**
**Formula**	$C_{56}H_{56}MgN_4O_2$	$C_{48}H_{40}N_4$	$C_{20}H_{16}N_4Ni$
**Formula weight**	841.35	672.84	371.08
**T (K)**	100(2)	100(2)	100(2)
**Wavelength (Å)**	0.71073	0.71073	0.71073
**Crystal system**	Triclinic	Orthorhombic	Monoclinic
**Space group**	*P*-1	*P*bca	*C*2/c
***a* (Å)**	13.082(3)	11.858(2)	10.837(2)
***b* (Å)**	13.520(3)	7.4875(14)	15.072(3)
***c* (Å)**	14.564(3)	20.191(4)	20.492(4)
**α (deg)**	66.980(4)	90	90
**β (deg)**	85.856(4)	90	105.33
**γ (deg)**	69.474(4)	90	90
**V ($Å^3$)**	2214.0(8)	1792.7(6)	3227.9(11)
**Z**	2	2	8
**Density (calc.) (Mg/m^3)**	1.262	1.246	1.527
**μ (mm^{-1})**	0.089	0.073	1.212
**F(000)**	896	712	1536
**Crystal size (mm^3)**	0.080 x 0.080 x 0.060	0.080 x 0.080 x 0.060	0.100 x 0.100 x 0.040
**Theta range for data collection (deg)**	1.667 to 29.064	2.017 to 26.429	1.030 to 26.884
**Index ranges**	$-17 \leq h \leq 17$, $-18 \leq k \leq 18$, $-19 \leq l \leq 19$	$-14 \leq h \leq 14$, $-9 \leq k \leq 9$, $-25 \leq l \leq 25$	$-13 \leq h \leq 13$, $-19 \leq k \leq 18$, $-25 \leq l \leq 25$
**Reflections collected**	33259	23556	46223
**Independent reflections**	11764[R(int) = 0.0934]	1841[R(int) = 0.0936]	3444[R(int) = 0.0993]
**Completeness to theta**	99.4 % 29.064 °	99.9 % 26.429 °	98.5 % 26.884 °
**Absorption correction**	Multi-scan	Multi-scan	Multi-scan
**Max. and min. transmission**	0.74 and 0.39	0.74 and 0.43	0.74 and 0.54
**Refinement method**	Full-matrix least-squares on F^2	Full-matrix least-squares on F^2	Full-matrix least-squares on F^2
**Data / restraints / parameters**	11764/ 0/ 572	1841/ 0/ 119	3444/ 0/ 228
**Goodness-of-fit on F^2**	1.032	1.083	1.080
**Final R indices [I>2sigma(I)]**	R1 = 0.0753, wR2 = 0.1857	R1 = 0.0469, wR2 = 0.1137	R1 = 0.0715, wR2 = 0.2199
**R indices (all data)**	R1 = 0.1247, wR2 = 0.2191	R1 = 0.0647, wR2 = 0.1257	R1 = 0.0874, wR2 = 0.2347
**Largest diff. peak and hole**	0.535 and -0.366 e.$Å^{-3}$	0.190 and -0.286 e.$Å^{-3}$	2.558 and -0.732 e.$Å^{-3}$

Table S4. Key Bond Lengths and angles

**Cpy-Cpy Bonds 3**			
C5B-C6B	1.374(5)	C25B-C26B	1.369(5)
C15B-C16B	1.380(4)	C35B-C36B	1.373(5)
C5A-C6A	1.382(5)		
**Mg-Mg Bonds 3**			
Mg1A-Mg2A	2.9418(17)	Mg1B-Mg4B	2.8332(18)
Mg1B-Mg2B	2.8246(16)	Mg2B-Mg3B	2.8288(18)
Mg3B-Mg4B	2.8379(17)		
**Mg-bipy-Mg angles 3**			
Mg1B-Bipy-Mg2B	103.4(2)	Mg4B-Bipy-Mg3B	103.5(2)
Mg3B-Bipy-Mg4B	104.0(2)	Mg2B-Bipy-Mg3B	102.9(2)
Mg1A-Bipy-Mg2A	108.8 (2)		
**Mg-Mg-Mg angles 3**			
Mg4B-Mg1B-Mg2B	90.0(2)	Mg2B-Mg3B-Mg4B	89.9(2)
Mg1B-Mg2B-Mg3B	90.1(2)	Mg3B-Mg4B-Mg1B	90.0(2)
**Cpy-Cpy Bonds 5**			
C5-C6	1.443(3)	C29-C30	1.442(3)
**Cpy-Cpy Bonds L2**			
C1-C1	1.496(3)		
**Cpy-Cpy Bonds 6**			
C5-C6	1.490(4)		
**Cpy-Cpy Bonds 2**			
C5B-C6B	1.443(9)	C1A-C1A	1.436(13)
C10A-C10A	1.425(14)		
**Cpy-Cpy Bonds (bipy)$_2$Mg(THF)$_2$**			
C5-C6	1.422(3)		

S7. Computational details

The molecular models were computed without truncations or symmetry constraints. Calculations were run using the Gaussian 16 program, Revision B.01.[22] Geometries were fully optimized (no imaginary frequencies) at the PBE0/6-31G+(d,p) level,[23-26] including the D3BJ empirical dispersion correction from Grimme[27] and the IEFPCM[28-29] solvent model with the parameters for tetrahydrofuran (THF), unless stated otherwise. All energies are given at 298.15 K. NMR spectra were calculated at the same level of theory, using the GIAO method[30] with tetramethylsilane as reference.
For the orbital pictures (made with ChemCraft version 1.8 build 610b),[31] the isovalue was set to 0.03 for all LUMO/HOMO/SOMO orbitals and to 0.004 for all spin densities.

<u>Dinuclear 3-Mg_2</u>

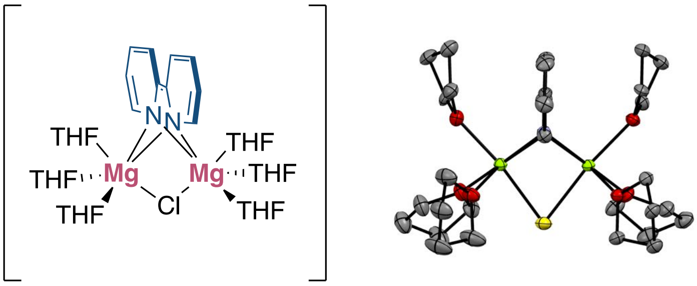

Figure S32. ChemDraw drawing (left) and X-ray structure (right) of dinuclear **3-Mg_2**

The dinuclear **3-Mg_2** species is comprised of one bipy ligand, one chloride, two Mg atoms and six THF molecules. If neutral, this amounts to a total of 101 atoms and 363 electrons and the system could *a priori* be doublet (one unpaired electron) or a quartet (three unpaired electrons). The complex was computed as a neutral species (doublet and quartet spin states), as a cation (singlet, assuming one electron was donated to **3-Mg_4**) and as an anion (singlet, assuming one electron was accepted from **3-Mg_4**). The starting geometry was taken from the experimental X-ray structure.

Charge	Spin state	Mg-N-Mg angle (average)	Mg-Mg distance (Å)	C(py)-C(py) distance (Å)	ΔG (kcal/mol)
0	Doublet	83.8	2.90	1.39	0.0
0	Quartet	83.7	2.91	1.43	13.8
-1	Singlet	82.7	2.94	1.38	-
+1	Singlet	83.2	2.95	1.38	-
**X-ray structure**		83.1	2.94	1.38	-

Table S5. Comparison of geometric parameters of the neutral (doublet/quartet), anionic and cationic **3-Mg_2**.

Table S5 shows that in terms of geometry, both charged forms of **3-Mg$_2$** have a Mg-Mg distance that is closer to the X-ray structure (2.94 Å, Table S4) than the computed neutral form.

Tetranuclear 3-Mg$_4$

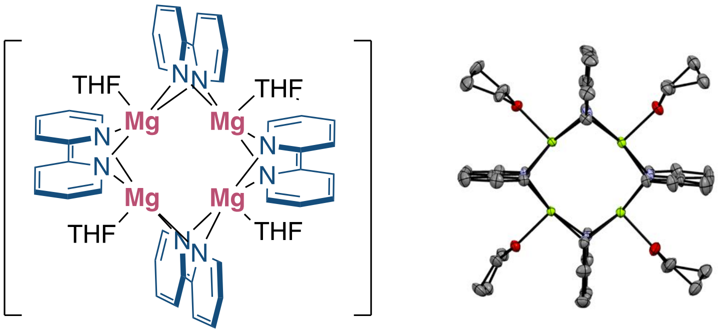

Figure S33. ChemDraw drawing (left) and X-ray structure (right) of the tetranuclear **3-Mg$_4$**

The full **3-Mg$_4$** structure was selected for computations, consisting of four bipy ligands, four Mg atoms, and four THF molecules. If neutral, this amounts to a total of 136 atoms and 536 electrons. Depending on the orbital distribution of the electrons, the system could be singlet (no unpaired electrons) or triplet (two unpaired electrons). The quintet (four unpaired electrons) and higher states were not considered in our calculations.
Similarly to the dinuclear **3-Mg$_4$**, the anionic and cationic **3-Mg$_4$** were also computed (using the X-ray structure as starting geometry) and compared to the neutral one.

Charge	Multiplicity	Mg-Mg (Å)	C(py)-C(py) (Å)	dG (kcal/mol)
**0**	Singlet	3.04	1.38	0.0
**0**	Triplet	2.83	1.39	+12.5
**-1**	Doublet	2.82	1.38	-
**+1**	Doublet	3.05	1.39	-
**X-ray structure**		2.83	1.37	-

Table S6. Comparison between the neutral (singlet state), anionic and cationic **3-Mg$_4$**

There is a clear difference in Mg-Mg distance between the cationic and anionic forms of **3-Mg$_4$**. In the crystal structure, that distance is around 2.83 Å (Table S4), which is closer to the anion than the cation or neutral species.

In order to establish the preferred oxidation states of the **3-Mg$_2$** and **3-Mg$_4$** fragments, the Gibbs free energies were analyzed at the PBE0-D3BJ/6-31+G(d,p) level as follows:

$$[3 - Mg_2]^0 + [3 - Mg_4]^0 \rightarrow [3 - Mg_2]^+ + [3 - Mg_4]^- \qquad \Delta G = -11.7\ kcal/mol$$
$$[3 - Mg_2]^0 + [3 - Mg_4]^0 \rightarrow [3 - Mg_2]^- + [3 - Mg_4]^+ \qquad \Delta G = +67.1\ kcal/mol$$

These energies support that the **3-Mg$_2$/3-Mg$_4$** system most likely is an ion pair where **3-Mg$_2$** is the cation and **3-Mg$_4$** is the anion ([**3-Mg$_2$**]$^+$/ [**3-Mg$_4$**]$^-$).

The energy of the divalent ion pair [**3-Mg$_2$**]$^{2+}$/ [**3-Mg$_4$**]$^{2-}$ relative to the mono-valent ion pair [**3-Mg$_2$**]$^+$/ [**3-Mg$_4$**]$^-$ was also investigated. The structures were optimized with the same computational protocol as above, but adding the "Surface=SAS" additional input for the solvation model (this was necessary to achieve convergence for [**3-Mg$_4$**]$^{2-}$), and the Gibbs free energy difference was as follows:

$$[3 - Mg_2]^+ + [3 - Mg_4]^- \rightarrow [3 - Mg_2]^{2+} + [3 - Mg_4]^{2-} \qquad \Delta G = +86.2\ kcal/mol$$

This supports the hypothesis that the two complexes prefer to be singly charged rather than doubly charged.

The hypothesis that [**3-Mg4**]$^-$ could contain a hydride rather than be an electride was also investigated. A structure containing a hydride at the center of the square formed by the 4 Mg atoms was successfully optimized (Figure S34) and compared in structure to both the X-ray and electride structures (Table S7). There is no significant difference between the geometries of the hydride and the electride.

Structure	Mg-Mg (Å)	C(py)-C(py) (Å)
**[3-Mg$_4$]$^-$ (electride)**	2.82	1.38
**[3-Mg$_4$-H]$^-$ (hydride)**	2.82	1.38
**X-ray structure**	2.83	1.37

Table S7. Comparison between the electride (doublet state) [**3-Mg$_4$**]$^-$ and hydride (singlet state) [**3-Mg$_4$-H**]$^-$

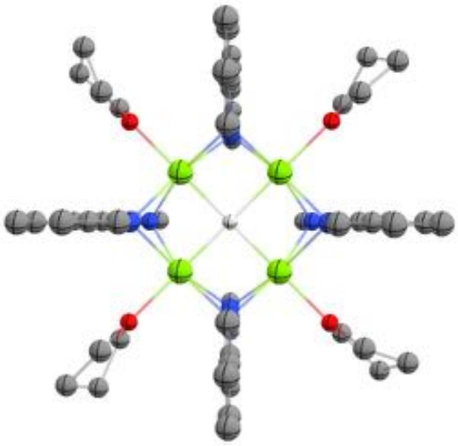

Figure S34. Computed structure of the hydride [**3-Mg$_4$-H**]$^-$.

The NMR spectra of both the hydride and electride were also computed, and were consistent within ±0.5 ppm accuracy with the experimental spectrum, which is within acceptable range for such calculations.[32] From comparing these computed spectra to the experimental spectra, we were not able to identify a signal that would be characteristic for a hydride.

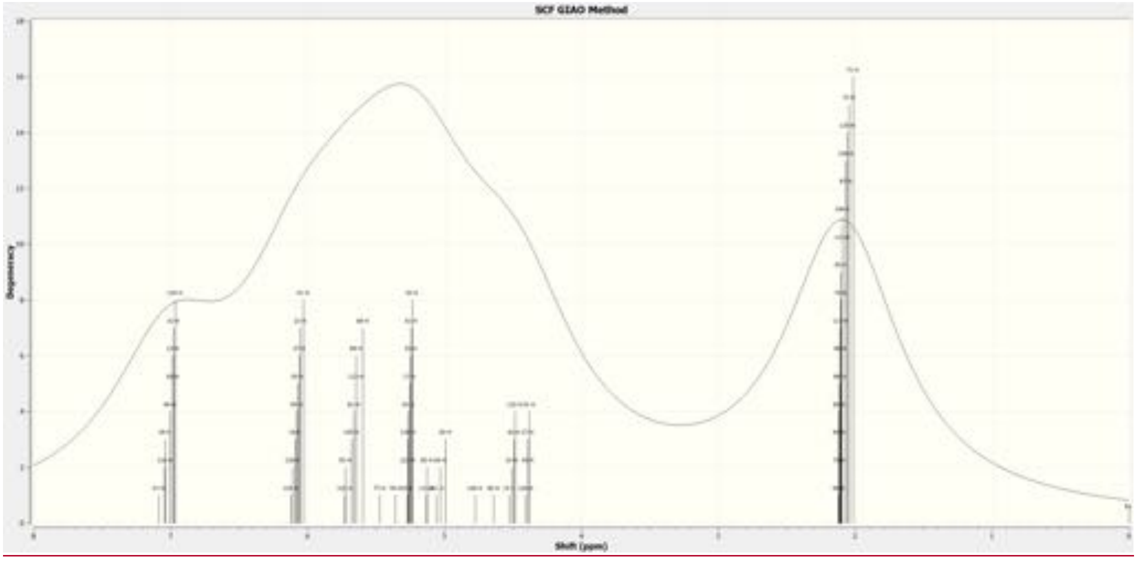

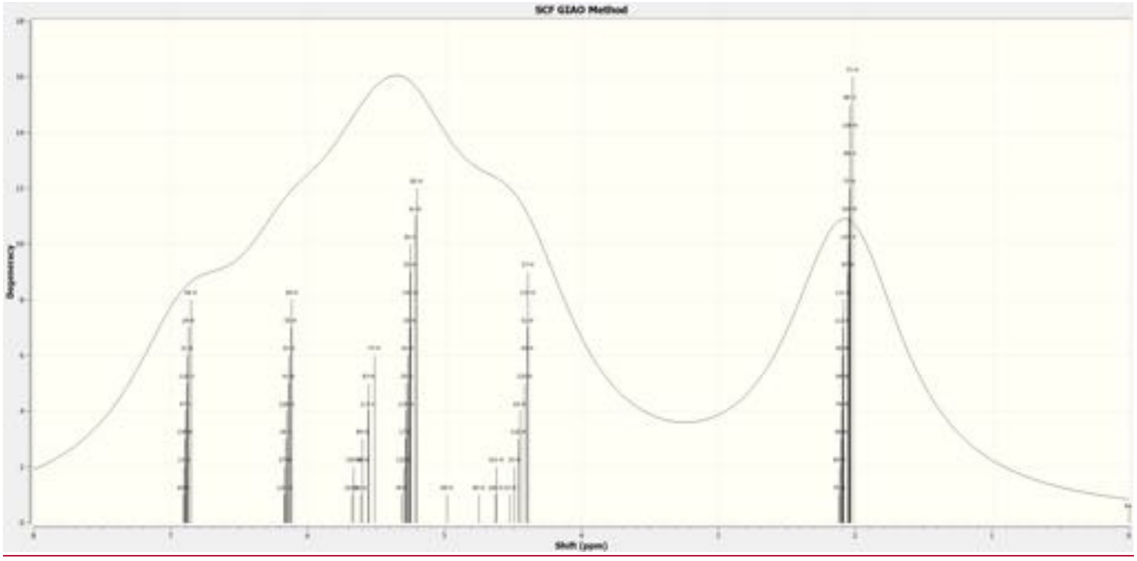

Figure S35. Computed NMR spectra for the electride [**3-Mg₄**]⁻ (top) and the hydride [**3-Mg₄-H**]⁻ (bottom).

<u>Bipy ligand</u>

Given the propensity for the bipy ligand to act as a neutral, anionic (-1) or dianionic (-2) species depending on the system,[33-36] we analyzed the bipy ligands in both Mg clusters to determine their nature. The free bipy ligand was first computed separately with different oxidation states and then compared to the computed and experimental bipy ligand in the clusters.

Species	Charge	C(py)-C(py)
**Bipy**	0	1.49
	-1	1.44
	-2	1.39
**[3-Mg$_2$]$^+$ (computed)**	+1	1.38
**[3-Mg$_4$]$^-$ (computed)**	-1	1.38
**[3-Mg$_2$] (X-ray)**		1.38
**[3-Mg$_4$] (X-ray)**		1.37

Table S8. Calculated C(py)-C(py) distances in the isolated bipy ligand (neutral, anionic and dianionic) and the optimized [**3-Mg$_2$**]$^+$ and [**3-Mg$_4$**]$^-$ structures, as well as the crystal structure. For [**3-Mg$_4$**]$^-$ and the X-ray [**3-Mg$_4$**], the average C(py)-C(py) distance is reported.

The C(py)-C(py) distance varies significantly depending on the charge of the bipy, as shown in Table S8. The calculated C(py)-C(py) distances in [**3-Mg$_2$**]$^+$ and [**3-Mg$_4$**]$^-$ correlate well with the value for the free dianionic bipy, as well as the distance measured in the X-ray structure. This is consistent with an assignment where all Mg atoms in **3-Mg$_2$** and **3-Mg$_4$** are Mg(II).

Orbital analysis of **3-Mg_2** and **3-Mg_4**

For [**3-Mg_2**]$^+$, the HOMO and LUMO orbitals are nr. 181 and 182 respectively. The HOMO is localized on both sides of the bipy ligand, and so is the LUMO.

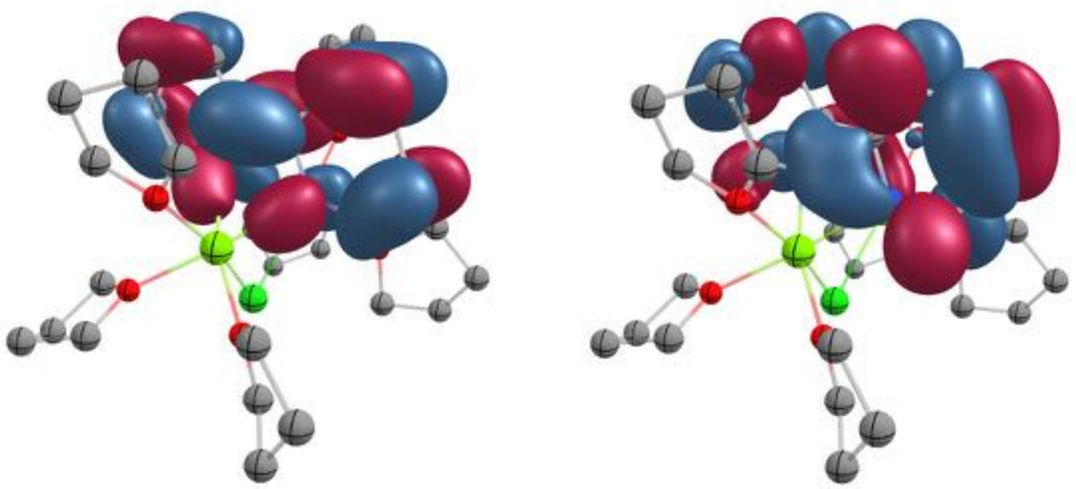

Figure S36. HOMO (left) and LUMO (right) of [**3-Mg_2**]$^+$

For [**3-Mg_4**]$^-$, the HOMO and LUMO were determined to be orbitals 269 and 270, respectively. Interestingly, the SOMO of [**3-Mg_4**]$^-$ is not the HOMO, but rather the α-HOMO-4 orbital. The α-HOMO-4 is localized in the middle of the square created by the four Mg atoms, in agreement with the assignment of the electride electron to this orbital. The same SOMO orbital was observed with other DFT functionals (ωB97XD, BP86), which supports that this result is not an artifact. The SOMO-HOMO inversion indicates that [**3-Mg_4**]$^-$ does not follow the Aufbau principle, however, we note that the electride electron is supposed to be free (not bound by an atom or molecule) and hence it should not enter into the molecular orbitals of 3-Mg_4. Therefore, one may view [**3-Mg_4**]$^-$ as two species, a neutral [**3-Mg_4**] part (which follows the Aufbau principle) and a free electron.

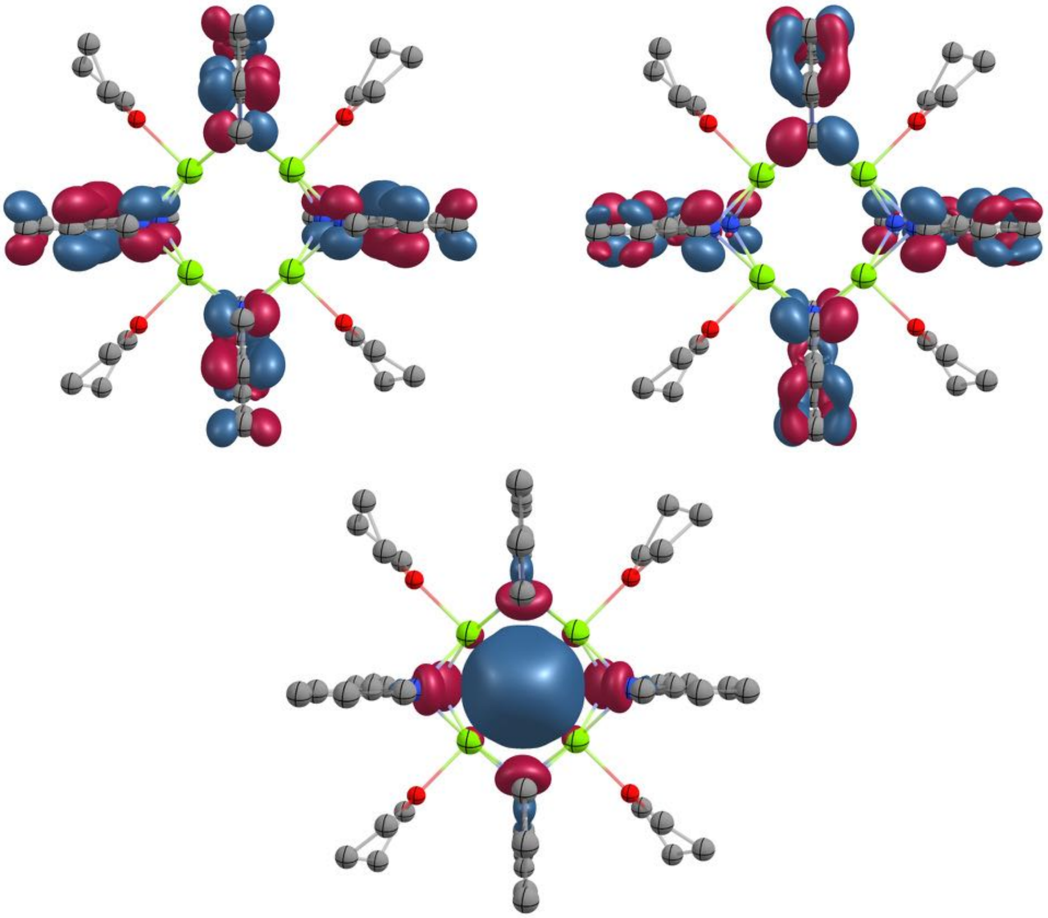

Figure S37. HOMO (top left), LUMO (top right) and SOMO (bottom) of [**3-Mg**$_4$]$^-$.

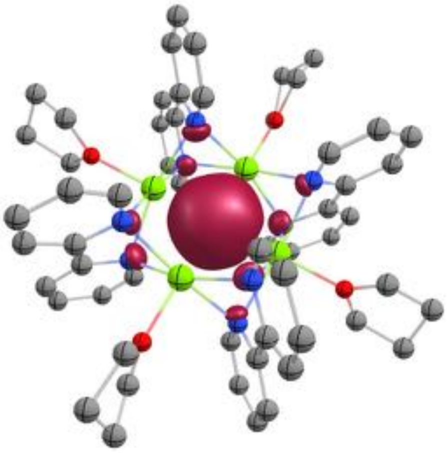

Figure S38. Spin density of [**3-Mg**$_4$]$^-$.

Structure **5**

The structure was optimized with the same computational protocol used for [**3-Mg**$_2$]$^+$ and [**3-Mg**$_4$]$^-$, in the closed-shell singlet (S = 0) and triplet (S = 1) state. The triplet is 13.7 kcal/mol more stable than the closed-shell singlet, indicating that **5** is a biradical. Orbital

analysis of the triplet system shows that the HOMO and LUMO orbitals are nr. 205 and 206, respectively. Similarly to [**3-Mg₂**]$^+$, the HOMO is located on the bipy ligands.

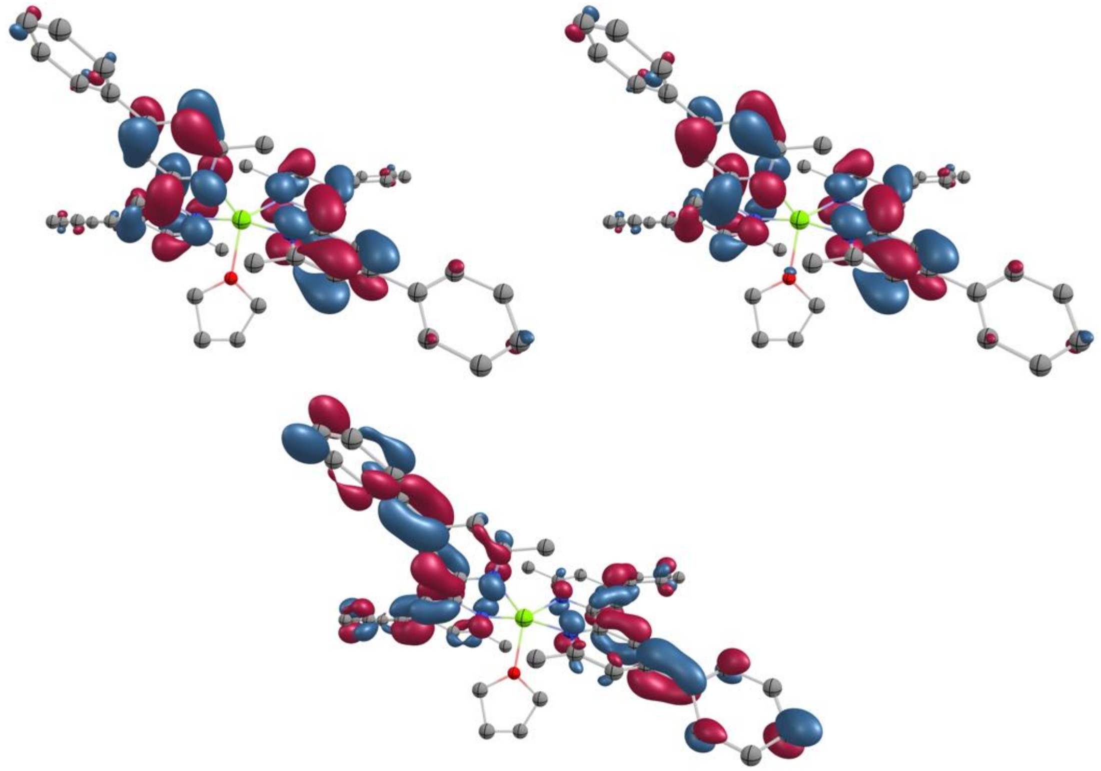

Figure S39. SOMO (orbital 204 top left, 205 top right) and LUMO (bottom) of structure **5** (triplet state)

The two SOMOs for this structure are orbitals 204 and 205, which follows the Aufbau principle. Both SOMOs have the same shape, but the main difference between the two is the inversion of phase on one of the bipy ligands.

Considering that the two spin sites in **5** are far away from each other (with one unpaired electron on each anionic bipy ligand), we also evaluated if the structure could be an open-shell singlet rather than a triplet. A broken symmetry singlet calculation was run using the triplet state geometry and wavefunction as starting guess, and the resulting optimized ($M_S = 0$) structure was only 0.7 kcal/mol higher in energy than the triplet ($S = 1$) structure. Analysis of the spin density shows that in the triplet state, each bipy ligand holds one α-spin, whereas in the broken symmetry singlet, one bipy has an α-spin and the other one has a β-spin (see Figure S40 below).

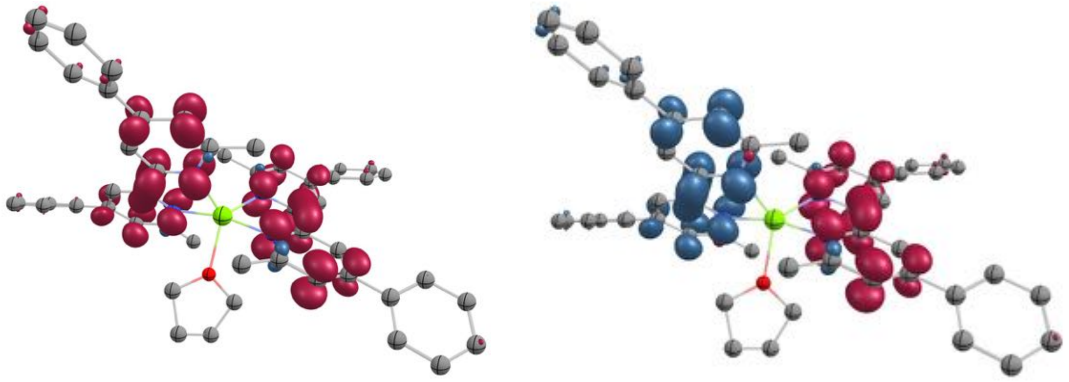

Figure S40. Comparison between the spin densities of structure **5** in the triplet state (left, S = 1) and in the broken symmetry singlet state (right, $M_S = 0$).

Given the small differences in energy between the triplet and the broken symmetry singlet state of **5**, it can be expected that both spin states would be thermally populated, which could make **5** a spin-crossover complex.

QTAIM analysis of [**3-Mg$_4$**]

The wave function of the molecules was extracted from the formatted checkpoint files of the PBE0/6-31G+(d,p) computations and was further analyzed within the framework of the quantum theory of atoms in molecules, QTAIM,[37] by AIMAll suite of programs.[38] The electron density, Laplacian of the electron density, and interatomic (zero-flux) surfaces were plotted by AIMStudio module, Figures S40, S41, and S42. The bonding between the Mg atoms in [**3-Mg$_4$**]$^-$ was examined by checking the electron delocalization index within the framework of QTAIM. The delocalization index values were found to be less than 0.01 atomic units (au, 1 au = 1 electron) that is not even characteristic of a noncovalent interaction.

To assess aromatic/antiaromatic character of the bipyridine dianion, [**3-Mg$_2$**]$^+$ was selected and GIAO-NMR[39] computations at the PBE0/6-31G+(d,p) level were performed. The ring current intensity was measured as the integral of the current density passing through the interatomic surfaces between adjacent carbon atoms of the bipyridine ligands. The current density was assessed to be -2.6 nA.T$^-$, which is characteristic of a weakly antiaromatic system.

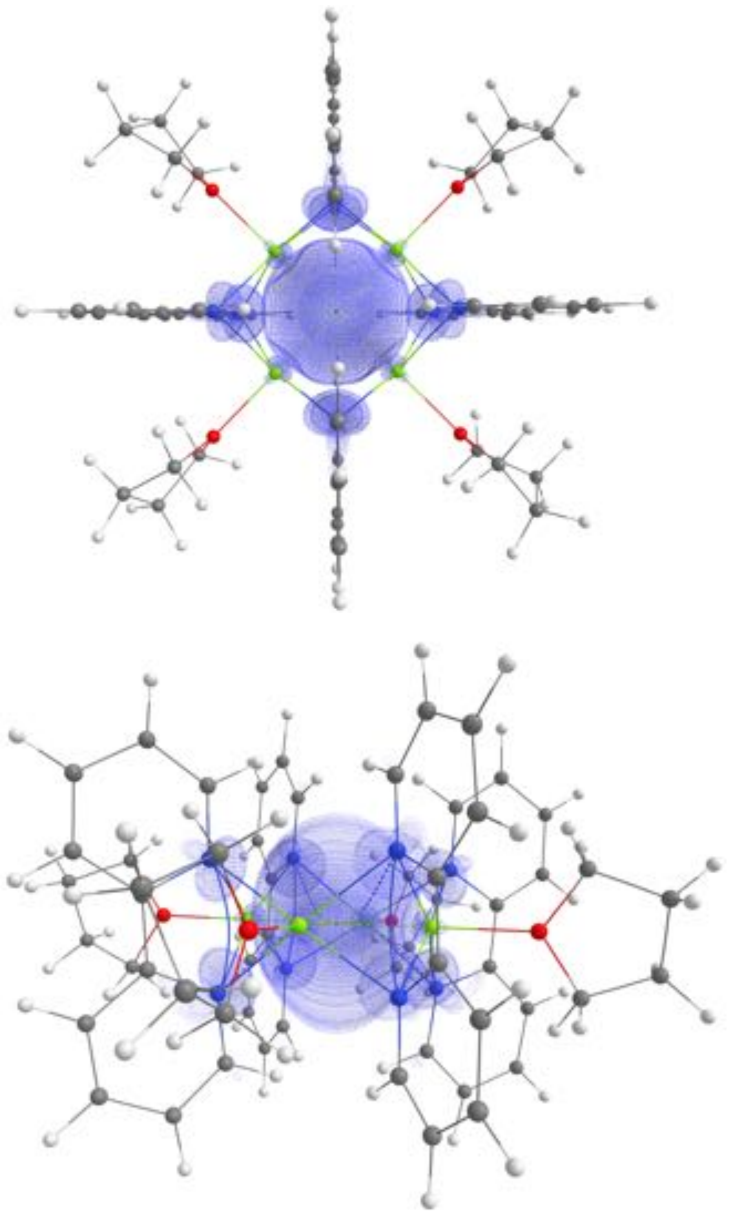

Figure S41. The spin density plots at 0.05 atomic units (au) isosurface.

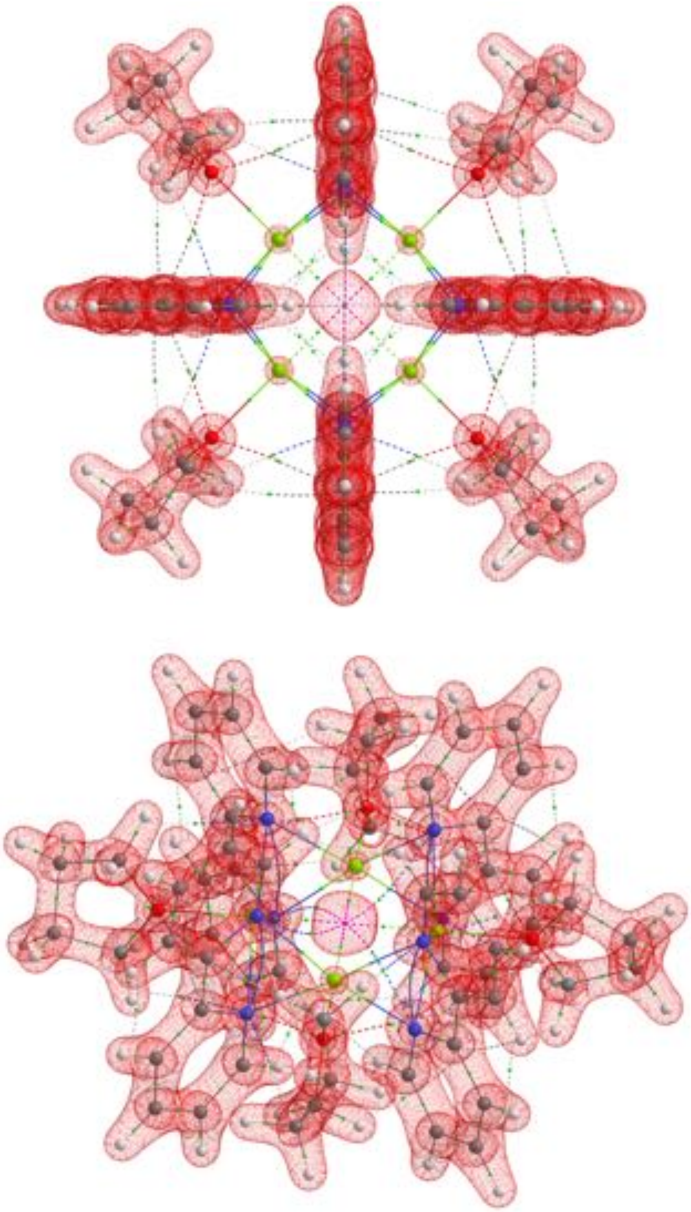

Figure S42. The 3D-isosurface of the Laplacian of the electron density at -0.005 atomic units (au) value from two directions. The central electron concentration corresponding to the electride electron is evident in both figures.

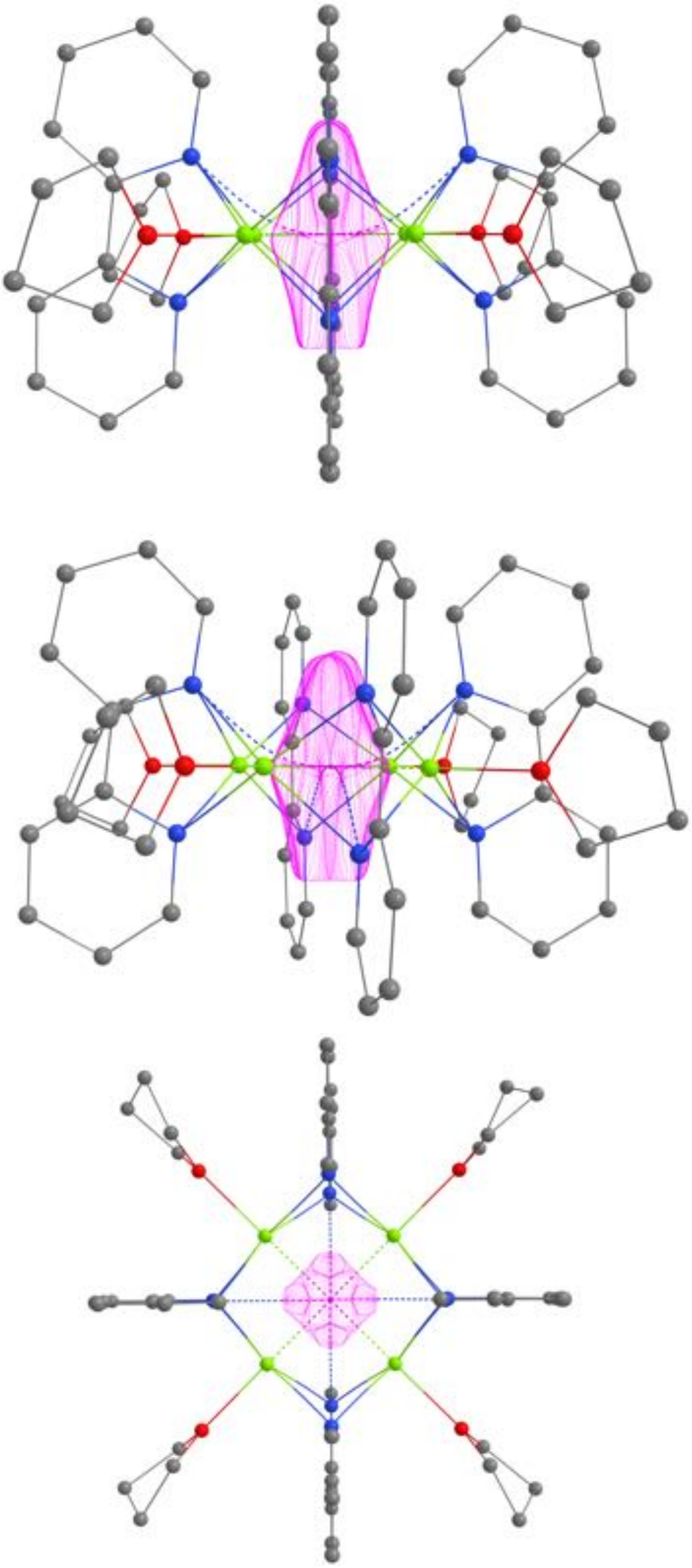

Figure S43. The atomic basin of the NNA enclosed in the interatomic zeroflux surfaces from different angles.

Atoms	Spin Density	Atoms	Spin Density	Atoms	Spin Density	Atoms	Spin Density
Mg1	0.0486	C36	0.0009	H71	0.0000	H106	0.0000
Mg2	0.0458	H37	0.0006	H72	0.0000	C107	0.0000
Mg3	0.0483	C38	0.0011	C73	0.0000	H108	0.0000
Mg4	0.0459	C39	0.0028	H74	0.0000	H109	0.0000
N5	0.0528	C40	0.0004	H75	0.0000	C110	0.0000
O6	-0.0001	H41	0.0007	C76	0.0000	H111	0.0000
N7	0.0387	C42	0.0010	H77	0.0000	H112	0.0000
N8	0.0383	H43	0.0000	H78	0.0000	H113	0.0000
N9	0.0526	C44	-0.0013	O79	-0.0001	H114	0.0000
N10	0.0530	H45	0.0004	C80	0.0000	N115	0.0532
N11	0.0385	C46	0.0033	H81	0.0000	N116	0.0382
C12	0.0012	H47	0.0048	H82	0.0000	C117	0.0034
H13	0.0019	C48	0.0034	C83	0.0000	H118	0.0049
C14	-0.0010	H49	0.0047	H84	0.0000	C119	-0.0013
H15	0.0002	C50	-0.0014	H85	0.0000	H120	0.0004
C16	-0.0005	H51	0.0004	C86	0.0000	C121	0.0010
H17	0.0000	C52	0.0010	H87	0.0000	H122	0.0000
C18	0.0009	H53	0.0000	C88	0.0000	C123	0.0004
H19	0.0006	C54	0.0004	H89	0.0000	H124	0.0008
C20	0.0011	H55	0.0007	H90	0.0000	C125	0.0029
C21	0.0029	C56	0.0029	C91	0.0000	C126	0.0011
C22	0.0004	C57	0.0011	H92	0.0000	C127	0.0009
H23	0.0007	C58	0.0009	H93	0.0000	H128	0.0006
C24	0.0010	H59	0.0006	C94	0.0000	C129	-0.0005
H25	0.0000	C60	-0.0005	H95	0.0000	H130	0.0000
C26	-0.0013	H61	0.0000	H96	0.0000	C131	-0.0009
H27	0.0004	C62	-0.0010	C97	0.0000	H132	0.0002
C28	0.0034	H63	0.0002	H98	0.0000	C133	0.0012
H29	0.0047	C64	0.0012	H99	0.0000	H134	0.0018
C30	0.0012	H65	0.0018	C100	0.0000	H135	0.0000
H31	0.0018	O66	-0.0001	H101	0.0000	C136	0.0000
C32	-0.0009	C67	0.0000	H102	0.0000	NNA137	0.3803
H33	0.0002	H68	0.0000	O103	-0.0001		
C34	-0.0005	H69	0.0000	C104	0.0000		
H35	0.0000	C70	0.0000	H105	0.0000		

Table S9. Atoms and their spin density. The atom numbering is according to the numbers given in the cartesian coordinate of the optimized structure.

S8. References

1. Yakhvarov, D. G.; Hey-Hawkins, E.; Kagirov, R. M.; Budnikova, Y. H.; Ganushevich, Y. S.; Sinyashin, O. G. Electrocatalytic reduction of aryldichlorophosphines with the (2,2′-bipyridine)nickel complexes. *Russ. Chem. Bull.* **2007,** *56* (5), 935-942.
2. Powers, D. C.; Anderson, B. L.; Nocera, D. G. Two-Electron HCl to H_2 Photocycle Promoted by Ni(II) Polypyridyl Halide Complexes. *J. Am. Chem. Soc.* **2013,** *135* (50), 18876-18883.
3. Tortajada, A.; Duan, Y.; Sahoo, B.; Cong, F.; Toupalas, G.; Sallustrau, A.; Loreau, O.; Audisio, D.; Martin, R. Catalytic Decarboxylation/Carboxylation Platform for Accessing Isotopically Labeled Carboxylic Acids. *ACS Catal.* **2019,** *9* (7), 5897-5901.
4. Creutz, C. Bipyridine Radical Ions. *Comments Inorg. Chem.* **1982,** *1* (5), 293-311.
5. Henne, B. J.; Bartak, D. E. Metal-vapor synthesis and electrochemistry of bis(bipyridyl)nickel(0). *Inorg. Chem.* **1984,** *23* (3), 369-373.
6. Wang, M.; England, J.; Weyhermüller, T.; Wieghardt, K. Electronic Structures of "Low-Valent" Neutral Complexes $[NiL_2]^0$ (S = 0; L = bpy, phen, tpy) – An Experimental and DFT Computational Study. *Eur. J. Inorg. Chem.* **2015,** *2015* (9), 1511-1523.
7. Behrens, H.; Müller, A. Zur Kenntnis der Chemie der Metallcarbonyle und der Cyanokomplexe in flüssigem Ammoniak, XIII. Zur Kenntnis der Reaktionsweisen der Cyanometallate(O) K6[Cr(CN)6] und K4[Ni(CN)4]. *Z. Anorg. Allg. Chem.* **1965,** *341* (3-4), 124-136.
8. Schunn, R. A.; Ittel, S. D.; Cushing, M. A.; Baker, R.; Gilbert, R. J.; Madden, D. P., Bis(1,5-Cyclooctadiene)Nickel(0). In *Inorg. Synth.*, 1990; pp 94-98.
9. Akira, M.; Yasuzo, U.; Takamichi, Y.; Hironori, K. The Electrochemical Behavior of the Low-valent Transititon-metal Complexes. I. Nickel-2,2′-Dipyridyl Complexes. *Bull. Chem. Soc. Jpn.* **1972,** *45* (5), 1438-1442.
10. Mahmut, A.; Takakazu, Y. Kinetic Study of Ligand Exchange Reactions of Bis(1,5-cyclooctadiene)nickel(0) with 2,2′-Bipyridine, 4,4′-Dimethyl-2,2′-bipyridine, and 4,4′,5,5′-Tetramethyl-2,2′-bipyridine. *Bull. Chem. Soc. Jpn.* **1999,** *72* (6), 1255-1261.
11. Dinjus, E.; Walther, D.; Kaiser, J.; Sieler, J.; Ngoc Thanh, N. 2,2′-dipyridyl-1,5-cyclooctadiennickel(0): kristall-und molekülstruktur. *J. Organomet. Chem.* **1982,** *236* (1), 123-130.
12. Davies, J.; Janssen-Müller, D.; Zimin, D. P.; Day, C. S.; Yanagi, T.; Elfert, J.; Martin, R. Ni-Catalyzed Carboxylation of Aziridines en Route to β-Amino Acids. *J. Am. Chem. Soc.* **2021,** *143* (13), 4949-4954.
13. Buchammagari, H.; Toda, Y.; Hirano, M.; Hosono, H.; Takeuchi, D.; Osakada, K. Room Temperature-Stable Electride as a Synthetic Organic Reagent: Application to Pinacol Coupling Reaction in Aqueous Media. *Org. Lett.* **2007,** *9* (21), 4287-4289.
14. Data reduction with CrysAlisPro 1.171.40.35 (Rigaku OD, 2018).
15. Empirical absorption correction using spherical harmonics implemented in Scale3 Abspack scaling algorithm, CrysAlisPro 1.171.40.35 (Rigaku OD, 2018).
16. Data collection with APEX II version v2013.4-1. Bruker (2007). Bruker AXS Inc., Madison, Wisconsin, USA.
17. Data reduction with Bruker SAINT version V8.30c. Bruker (2007). Bruker AXS Inc., Madison, Wisconsin, USA.
18. SADABS: V2012/1 Bruker (2001). Bruker AXS Inc., Madison, Wisconsin, USA. Blessing, Acta Cryst. 1995, A51, 33-38.
19. Sheldrick, G. M. SHELXT– Integrated space-group and crystal-structure determination. *Acta Crystallographica Section A Foundations and Advances* **2015,** *71* (1), 3-8.
20. Hübschle, C. B.; Sheldrick, G. M.; Dittrich, B. ShelXle: a Qt graphical user interface forSHELXL. *J. Appl. Crystallogr.* **2011,** *44* (6), 1281-1284.

21. Sheldrick, G. M. Crystal structure refinement withSHELXL. *Acta Crystallographica Section C Structural Chemistry* **2015,** *71* (1), 3-8.
22. Frisch, M. J.; Trucks, G. W.; Schlegel, H. B.; Scuseria, G. E.; Robb, M. A.; Cheeseman, J. R.; Scalmani, G.; Barone, V.; Petersson, G. A.; Nakatsuji, H.; Li, X.; Caricato, M.; Marenich, A. V.; Bloino, J.; Janesko, B. G.; Gomperts, R.; Mennucci, B.; Hratchian, H. P.; Ortiz, J. V.; Izmaylov, A. F.; Sonnenberg, J. L.; Williams; Ding, F.; Lipparini, F.; Egidi, F.; Goings, J.; Peng, B.; Petrone, A.; Henderson, T.; Ranasinghe, D.; Zakrzewski, V. G.; Gao, J.; Rega, N.; Zheng, G.; Liang, W.; Hada, M.; Ehara, M.; Toyota, K.; Fukuda, R.; Hasegawa, J.; Ishida, M.; Nakajima, T.; Honda, Y.; Kitao, O.; Nakai, H.; Vreven, T.; Throssell, K.; Montgomery Jr., J. A.; Peralta, J. E.; Ogliaro, F.; Bearpark, M. J.; Heyd, J. J.; Brothers, E. N.; Kudin, K. N.; Staroverov, V. N.; Keith, T. A.; Kobayashi, R.; Normand, J.; Raghavachari, K.; Rendell, A. P.; Burant, J. C.; Iyengar, S. S.; Tomasi, J.; Cossi, M.; Millam, J. M.; Klene, M.; Adamo, C.; Cammi, R.; Ochterski, J. W.; Martin, R. L.; Morokuma, K.; Farkas, O.; Foresman, J. B.; Fox, D. J. *Gaussian 16 Rev. C.01*, Wallingford, CT, 2016.
23. Adamo, C.; Barone, V. Toward reliable density functional methods without adjustable parameters: The PBE0 model. *J. Chem. Phys.* **1999,** *110*, 6158-6170.
24. Petersson, G. A.; Bennett, A.; Tensfeldt, T. G.; Al-Laham, M. A.; Shirley, W. A.; Mantzaris, J. A complete basis set model chemistry. I. The total energies of closed-shell atoms and hydrides of the first-row elements. *J. Chem. Phys.* **1988,** *89* (4), 2193-2218.
25. Clark, T.; Chandrasekhar, J.; Spitznagel, G. W.; Schleyer, P. V. R. Efficient diffuse function-augmented basis sets for anion calculations. III. The 3-21+G basis set for first-row elements, Li–F. *J. Comput. Chem.* **1983,** *4* (3), 294-301.
26. Petersson, G. A.; Al-Laham, M. A. A complete basis set model chemistry. II. Open-shell systems and the total energies of the first-row atoms. *J. Chem. Phys.* **1991,** *94* (9), 6081-6090.
27. Grimme, S.; Ehrlich, S.; Goerigk, L. Effect of the damping function in dispersion corrected density functional theory. *J. Comput. Chem.* **2011,** *32* (7), 1456-1465.
28. Cossi, M.; Barone, V.; Cammi, R.; Tomasi, J. Ab initio study of solvated molecules: a new implementation of the polarizable continuum model. *Chem. Phys. Lett.* **1996,** *255* (4), 327-335.
29. Lipparini, F.; Scalmani, G.; Mennucci, B.; Cancès, E.; Caricato, M.; Frisch, M. J. A variational formulation of the polarizable continuum model. *J. Chem. Phys.* **2010,** *133* (1), 014106.
30. Wolinski, K.; Hinton, J. F.; Pulay, P. Efficient implementation of the gauge-independent atomic orbital method for NMR chemical shift calculations. *J. Am. Chem. Soc.* **1990,** *112* (23), 8251-8260.
31. Zhurko, G. A. *Chemcraft - graphical program for visualization of quantum chemistry computations.*, Ivanovo, Russia, 2005.
32. Beran, G. J. O., Calculating Nuclear Magnetic Resonance Chemical Shifts from Density Functional Theory: A Primer. In *eMagRes*, pp 215-226.
33. Bock, H.; Lehn, J.-M.; Pauls, J.; Holl, S.; Krenzel, V. Sodium Salts of the Bipyridine Dianion: Polymer $[(bpy)^{2-}\{Na^{+}(dme)\}_2]_\infty$, Cluster $[(Na_8O)^{6+}Na^{+}_6(bpy)_6^{2-}(tmeda)_6]$, and Monomer $[(bpy)^{2-}\{Na^{+}(pmdta)\}_2]$. *Angew. Chem. Int. Ed.* **1999,** *38* (7), 952-955.
34. Gore-Randall, E.; Irwin, M.; Denning, M. S.; Goicoechea, J. M. Synthesis and Characterization of Alkali-Metal Salts of 2,2′- and 2,4′-Bipyridyl Radicals and Dianions. *Inorg. Chem.* **2009,** *48* (17), 8304-8316.
35. Chisholm, M. H.; Huffman, J. C.; Rothwell, I. P.; Bradley, P. G.; Kress, N.; Woodruff, W. H. Bis(2,2'-bipyridyl)diisopropoxymolybdenum(II). Structural and spectroscopic evidence for molybdenum-to-bipyridyl π* bonding. *J. Am. Chem. Soc.* **1981,** *103* (16), 4945-4947.

36. Fedushkin, I. L.; Petrovskaya, T. V.; Girgsdies, F.; Köhn, R. D.; Bochkarev, M. N.; Schumann, H. Synthesis and Structure of the First Lanthanide Complex with the Bridging, Antiaromatic 2,2′-Bipyridine Dianion: $[\{Yb(\mu_2\text{-}N_2C_{10}H_8)(thf)_2\}_3]$. *Angew. Chem. Int. Ed.* **1999,** *38* (15), 2262-2264.
37. Bader, R. F. W., *Atoms in Molecules: A Quantum Theory.* Clarendon Press: Oxford, 1990.
38. Keith, T. A. *AIMAll*, Version 19.10.12; TK Gristmill Software: Overland Park KS, USA, 2019.
39. London, F. Théorie quantique des courants interatomiques dans les combinaisons aromatiques. *J. Phys. Radium* **1937,** *8* (10), 397-409.
